# Novel Azaborine-Based Inhibitors of Histone Deacetylases (HDACs)

**DOI:** 10.3390/molecules30194017

**Published:** 2025-10-08

**Authors:** Martin Behringer, Markus Schweipert, Enna E. Peters, Aleksandra Kopranovic, Franz-Josef Meyer-Almes

**Affiliations:** Department of Chemical Engineering and Biotechnology, University of Applied Sciences Darmstadt, Haardtring 100, 64295 Darmstadt, Germany; martin.behringer@h-da.de (M.B.); markus.schweipert@h-da.de (M.S.); ennaemilia.peters@stud.h-da.de (E.E.P.); aleksandra.kopranovic@h-da.de (A.K.)

**Keywords:** Histondeacetylase, HDAC inhibitors, azaborine, boron heterocycle, BN-heterocycle, BN-naphthalene, BN-indole, boron chemistry, heteroaromatic compounds, 1,2-azaborine

## Abstract

Aromatic ring systems appear ubiquitously in active pharmaceutical substances, such as FDA-approved histone deacetylase inhibitors. However, these rings reduce the water solubility of the molecules, which is a disadvantage during application. To address this problem, azaborine rings may be substituted for conventional aromatic ring systems. These are obtained by replacing two adjacent carbon atoms with boron and nitrogen. Incorporating B–N analogs in place of aromatic rings not only enhances structural diversity but also provides a strategy to navigate around patent-protected scaffolds. We synthesized azaborines, which are isosteric to naphthalene and indole, and utilized them as capping units for HDAC inhibitors. These molecules were attached to various aliphatic and aromatic linkers with different zinc-binding units, used in established active compounds. Nearly half of the twenty-four molecules tested exhibited inhibitory activity against at least one of the enzymes HDAC1, HDAC4, or HDAC8, with three compounds displaying IC_50_ values in the nanomolar range. We have therefore demonstrated that azaborine building blocks can be successfully incorporated into HDACis, resulting in a highly active profile. Consequently, it should be feasible to develop active substances containing azaborine rings against other targets.

## 1. Introduction

Histone deacetylases (HDACs) are enzymes that catalyze the removal of *N*-acetyl groups from lysine residues in histone proteins, resulting in a tighter chromatin configuration and typically a suppression of gene transcription [1]. HDACs have emerged as a promising target for therapeutic intervention in cancer and various other diseases [2]. HDACs are classified into four main classes [3]. Class I (HDAC1, 2, 3, 8) features zinc-dependent enzymes, mainly localized in the nucleus and primarily involved in transcriptional repression [4]. Class II is subdivided into IIa (HDAC4, 5, 7, 9) and IIb (HDAC6, 10). Class IIa enzymes shuttle between the cell nucleus and cytoplasm to control tissue-specific gene expression, while IIb enzymes act mainly in the cytoplasm, for example in microtubule dynamics [5]. Class III enzymes include the NAD^+^-dependent sirtuins (SIRT1–7). These serve multiple purposes in the nucleus, cytoplasm, and mitochondria, influencing aging, metabolism, and stress response [6]. Class IV (HDAC11) combines features of both classes I and II and regulates immunity and lipid metabolism [7].

Currently, five histone deacetylase inhibitors (HDAC inhibitors) are approved by the U.S. Food and Drug Administration (FDA): Vorinostat (Zolinza^®^) for the treatment of cutaneous T-cell lymphoma (CTCL) [8], Romidepsin (Istodax^®^) approved for CTCL and peripheral T-cell lymphoma (PTCL) [9,10], Belinostat (Beleodaq^®^) for peripheral T-cell lymphoma (PTCL), Panobinostat (Farydak^®^) for multiple myeloma [11,12], and Givinostat (Duvyzat^®^), which received marketing authorization for the treatment of muscular dystrophy and polycythemia vera in 2024 [13]. Tucidinostat (also known as Chidamide) is approved in China and Japan but is not yet FDA-approved in the United States [14].

**Figure 1:**
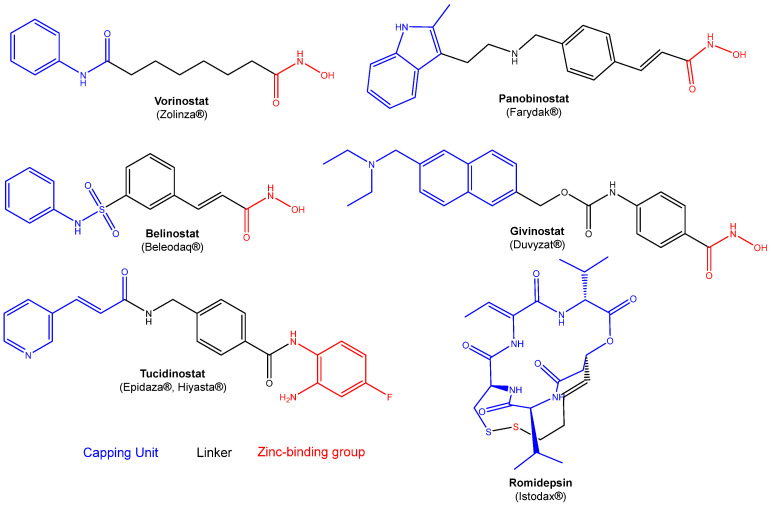
Regulatorily approved HDAC inhibitors with their structural features. The capping unit (blue) is connected to the zinc-binding group (red) via the carbon linker (black).

Inhibitors of HDACs usually consist of a capping unit, a linker of suitable length and steric requirements and a zinc-binding group (ZBG) to chelate a Zn^2+^ ion in the active site of the histone deacetylases. The capping group is mainly used to increase selectivity to target specific HDAC isoenzymes and consists of aromatic ring systems. However, the structure of Romidepsin differs from the previously mentioned inhibitors. It is a cyclic depsipeptide containing a disulfide bridge, which, upon reduction, releases a thiol that blocks the active site of HDAC enzymes. Figure 1 presents the structures of all approved HDAC inhibitors.

The presence of aromatic ring systems, common in many established drugs and bioactive compounds like HDAC inhibitors, can be disadvantageous to application as it reduces the aqueous solubility of the substance. Approximately 40% of approved drugs and almost 90% of drug candidates are poorly water-soluble, making it a significant challenge in pharmaceutical development, affecting drug efficacy and bioavailability [15]. Aromatic ring systems increase lipophilicity, which can negatively affect various ADME properties and toxicity, thereby reducing drugability [16]. In contrast, lipophilic efficiency shows the strongest correlation with drug quality [17].

**Figure 2:**
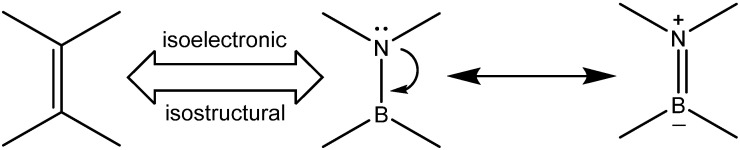
B−N isosteric substitution for C=C bonds.

The addition of hydrophilic groups such as hydroxyl, carboxyl, or polyethylene glycol groups (PEG linker) can increase the solubility in water of hydrophobic substances [18]. However, this also alters the chemical structure, which can have a negative effect on efficacy. Another interesting approach is the replacement of aromatic systems with 1,2-azaborines, where two adjacent carbon atoms are replaced by one nitrogen atom and one boron atom. Azaborines are isoelectronic and isostructural with their carbonaceous counterparts since the electron count and the planar ring structure remain similar (Figure 2). Incorporation of the 1,2-azaborine motif increases the hydrophilicity of the otherwise hydrophobic biphenyl motif due to the difference in electronegativity of boron and nitrogen, leading to a dipole moment of 2.1 D [19]. This implies that the nitrogen atom can serve as a hydrogen-bond donor via its free electron pair, a property absent in the carbon analog. The structural alteration may contribute both to improved solubility and to the establishment of specific hydrogen-bonding interactions with the target enzyme.

Azaborines are a promising type of compound that are not only interesting for creating diversity in organic molecules and basic research but also offer significant potential in the field of catalysis, material science and drug development [20].

**Figure 3:**
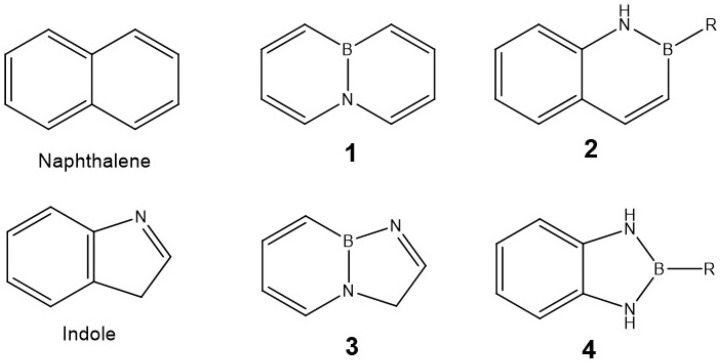
BN−naphthalene and BN-indole isosteres.

In the case of naphthalene, two isomers of 1,2-azaborine, the “internal” azaborine **1** and the “external” azaborine **2**, exist [21,22]. Given that synthetic building block **2** has already been employed in other molecules of medicinal relevance, we selected this scaffold as basis for the design of potential HDAC inhibitors [23,24]. To date, two azaborine isosteres of indole are known: the “fused” B–N indole **3**, where the adjacent bond within the bicyclic framework is substituted, and the 1,3,2-benzodiazaborole **4**, in which the C2–C3 double bond is replaced by a B–N bond (Figure 3) [25,26].

The indole ring system constitutes a privileged heterocyclic framework occurring in numerous natural and pharmacologically relevant molecules [27]. Similarly, naphthalene has been established as a versatile platform in medicinal chemistry [28].

The first example of biologically active monocyclic 1,2-azaborines was a CDK2 inhibitor, which showed improved biological activity, likely from additional H-bonding interaction. Notably, the BN-based molecule featured better in vivo oral bioavailability compared to the carbonaceous compound [29]. Furthermore, BN-naphthalenes were also synthesized and subsequently subjected to pharmacological investigation. For example, the benzazaborinine analogs of propranolol showed similar efficacy, physicochemical properties and ADME-Tox profiles to propranolol. Besides their good bioavailability these molecules were also able to penetrate the blood–brain barrier well after subcutaneous administration in rats. The results suggest that aromatic azaborine could be used as a substitute for naphthalene in drug development [23]. Bioactive BN-indoles, despite their potential significance in medicinal chemistry, remain largely unexplored, having neither been synthesized nor subjected to systematic investigation.

To assess whether azaborine-based HDAC inhibitors display activity comparable to that of approved compounds, BN-naphthalenes and BN-indoles were first synthesized, from which molecules were subsequently generated, that resemble the core framework of the approved inhibitors. Functionalization with zinc-binding groups was accomplished using diverse aliphatic and aromatic linkers. The chelating moieties included hydroxamic acids, as found in Vorinostat, Belinostat, Panobinostat, and Givinostat. Additionally, molecules featuring (2-Amino)-4-fluorobenzamides—a zinc-binding unit present in Tucidinostat—were synthesized. Subsequently, the IC_50_ values of these compounds were determined.

## 2. Results

### 2.1. Synthesis

#### 2.1.1. Synthesis and Functionalization of BN-Naphthalene Azaborine Building Block

**Scheme 1:**
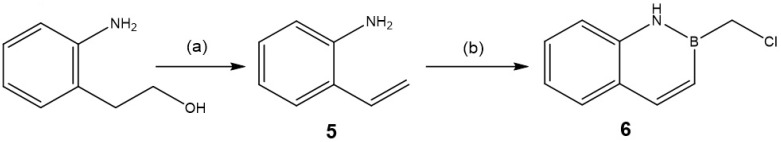
Synthesis of the azaborine building block 2-(chloromethyl)-1,2-dihydrobenzo[*e*][1,2]azaborinine **6**. (**a**) KOH, 180 °C, vacuum; (**b**) Potassium(chloromethyl)trifluoroborate; Et_3_N, SiCl_4_, CPME, 40 °C.

Since direct functionalization at the boron atom is often difficult, we synthesized 2-chloromethyl-2,1-borazaronaphthalene **6** starting with 2-(2-Aminophenyl)ethanol and KOH at high temperature and under vacuum [30]. The resulting 2-aminostyrene **5** was subsequently converted with potassium trifluoroborate as described by Molander et al. (Scheme 1) [31].

**Scheme 2:**
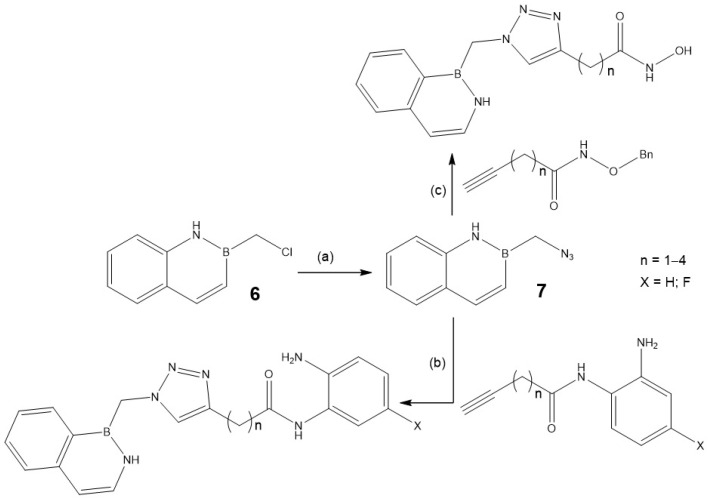
Further functionalization of 2-chloromethyl-2,1-borazaronaphthalene **6**, as Azaboine-N_3_ and following CuAAC. Alkyne linkers of different length were used, attached to protected hydroxamic acid or (para-fluorinated) 1,2-Phenylendiamine. (**a**) NaN_3_, 70 °C, -NaCl; (**b**) CuAAC; (**c**) CuAAC, deprotection (Pd/C, H_2_).

Azaborine **6** was converted into azide **7** using NaN_3_. This azide then enabled a copper(I)-catalyzed azide–alkyne cycloaddition (CuAAC) with an alkyne linker (Scheme 2). For linker synthesis, standard peptide coupling reactions involving HBTU and alkynoic acids were performed. The detailed synthesis procedures, along with NMR and LC-MS data, are provided in the Supplementary Materials.

Table 1 lists all compounds that were synthesized via the CuAAC reaction.

**Scheme 3:**
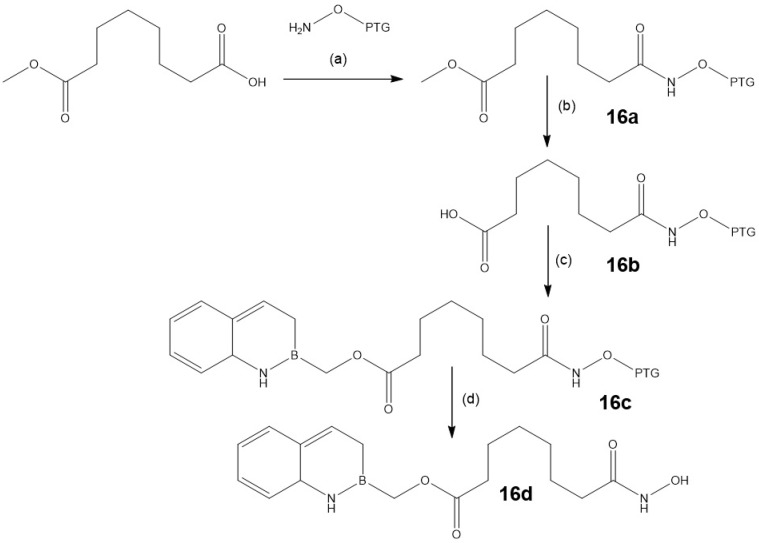
Representative synthesis route for Azaborine-based HDAC inhibitors with hydroxamic acid. (**a**) Coupling of the protected hydroxylamine (HBTU, DIPEA, CH_3_CN); (**b**) deprotection of the carboxylic acid (1. NaOH, 2. HCl); (**c**) reaction with Azaborine building block **6** (Cs_2_CO_3_; CH_3_CN; 70 °C; (**d**) deprotection: Pd/C, H_2_, PTG = Bn.

Dual linkers, featuring a protected carboxylic acid at one terminus and a zinc-binding moiety at the other, were also synthesized under standard coupling conditions. Hydroxamic acids were employed with either THP or benzyl protection. Following saponification and subsequent acidification to liberate carboxylic acid, the resulting free acid was directly coupled to azaborine building block **6**. Finally, the THP or benzyl protecting groups were removed, affording the target active compounds (Scheme 3).

Table 2 summarizes all compounds synthesized according to the procedure outlined in Scheme 3.

Besides this coupling strategy the azaborine building block **6** could also undergo reactions with amines. Using this synthetic method, a series of derivatives were prepared (Table 3).

#### 2.1.2. Synthesis and Functionalization of BN-Indole Azaborine Building Block

**Scheme 4:**
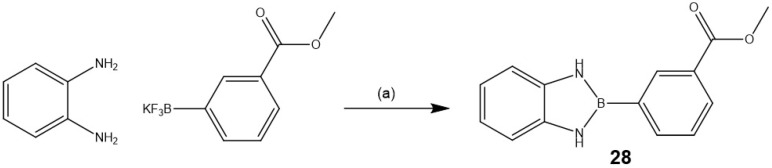
Synthesis route of BN-indoles. (**a**) BF_3_ NH_2_Et (3 eq); toluene/CPME (1:1) 0.5 M; 80 °C; 16 h.

For the synthesis of BN-indoles, we built upon the work of Davies and Molander, who reported a synthetic route enabling the preparation of these molecules from organotrifluoroborates and 1,2-phenylenediamines (Scheme 4) [32].

**Scheme 5:**
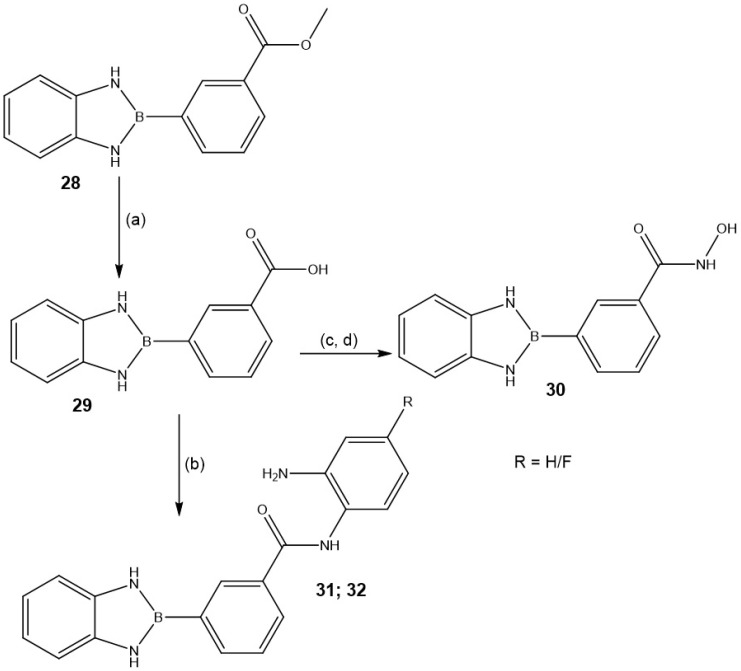
Further functionalization of BN-indoles (**a**) 1. NaOH, 2. HCl; (**b**) (4F)-1,2-Phenylendiamine, HBTU, DIPEA; (**c**) *O*-(Tetrahydropyran-2-yl)-hydroxylamine, HBTU, DIPEA; (**d**) HCl.

Saponification of the ester **28** followed by acidification afforded the free carboxylic acid **29**, which was subsequently converted into the corresponding hydroxamic acid **30** via coupling chemistry and deprotection (Scheme 5), as described in Section 2.1.1.

**Scheme 6:**
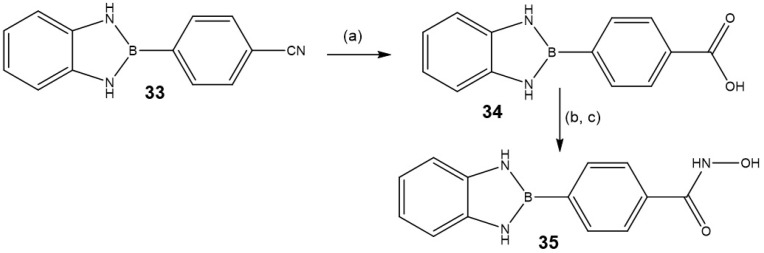
Nitrile hydrolysis with subsequent conversion to the hydroxamic acid (**a**) 1. NaOH, 2. HCl; (**b**) *O*-(Tetrahydropyran-2-yl)-hydroxylamine, HBTU, DIPEA; (**c**) HCl.

The nitrile **33** was successfully prepared following the procedure depicted in Scheme 4. Subsequent basic hydrolysis yielded the carboxylic acid **34**, which was further converted to the hydroxamic acid **35** (Scheme 6) according to the method described above.

All BN-indole-based molecules used for IC_50_ measurements are listed in Table 4.

### 2.2. Assessment of Inhibitory Activity

#### 2.2.1. IC_50_ Measurement of BN-Naphthalene-Based Compounds

The final compounds were evaluated in standard enzymatic assays against HDAC1, HDAC4, and HDAC8, and their IC_50_ values are listed in Table 5.

For compounds **8**, **11**, **14**, **15**, **19**, **20**, **21**, **22**, **26**, and **27**, only IC_50_ values > 35 µM were obtained for HDAC1, HDAC4, and HDAC8.

#### 2.2.2. IC_50_ Measurement of BN-Indole-Based Compounds

BN-indole-based HDAC inhibitors were evaluated using the same enzymatic assay conditions, and the corresponding IC_50_ values are given in Table 6.

For the determination of IC_50_ values, 50 mM DMSO stock solutions of the compounds were prepared and subsequently diluted with aqueous buffer. Triplicate measurements revealed no significant deviations over the course of several hours, suggesting that the compounds exhibit hydrolytic stability for at least 7 h.

## 3. Discussion

BN-naphthalenes and BN-indoles offer a promising strategy to introduce structural diversity into organic molecules. These innovative isosteric building blocks may possess biological activity comparable to or even surpassing that of their carbon analogs. Using HDAC inhibitors as a model system, we therefore sought to evaluate whether azaborine-based molecules can exert biological effects. Therefore, BN-naphthalenes and BN-indoles were synthesized starting from vinylaniline or 1,2-phenylenediamines and potassium trifluoroborate salts and were subsequently coupled with various linkers. These linkers were either aliphatic, as in Vorinostat, or aromatic, as exemplified by Belinostat and Panobinostat, which are all approved for clinical use. The zinc-binding moieties were also selected based on established and previously reported compounds, employing hydroxamic acids and (2-Amino)-4-fluorobenzamides [37,38]. In summary, we have efficiently synthesized a series of 24 potential HDAC inhibitors, bearing a BN-naphthalene or a BN-indole respectively as the capping unit.

Eleven of these compounds exhibited below 30% residual activity. These compounds were further evaluated via IC_50_ measurements, showing inhibitory effects on at least one HDAC enzyme. Notably, the three compounds **17**, **23** and **30**, were active against all tested HDACs, with HDAC8 consistently exhibiting the highest affinity. Compound **30** emerged as the most potent HDAC8 inhibitor, displaying an IC_50_ of only 0.20 µM. It also showed the strongest inhibition among all tested compounds on HDAC1 and HDAC4, with IC_50_ values of 0.975 µM and 1.35 µM, respectively. Substances **11**, **12**, and **13** are hydroxamic acids featuring a triazole ring. Substances **13** (IC_50_ = 23.1 µM and 3.3 µM for HDAC4 and HDAC8, respectively) and **12** (IC_50_ = 13.8 µM and 6.2 µM) exhibit markedly higher activity than **11** (IC_50_ > 35µM), indicating that the ethyl linker in **11** is too short.

Although 2-aminobenzamides can serve as zinc-binding units and have demonstrated acceptable IC_50_ values in similar studies [39], BN-indole derivatives containing 2-aminobenzamides, for example, displayed no detectable inhibitory activity (IC_50_ > 35 µM). Similarly, a series of BN-naphthalene-based compounds, including **14**, **15, 20**, **21**, and **26**, exhibit this behavior. In these cases, triazoles and phenyl rings were employed as linkers, along with aliphatic chains of varying lengths.

In contrast, several synthesized hydroxamic acids, beyond the previously mentioned **30**, also display acceptable IC_50_ values; for instance, **18** exhibits an IC_50_ of 0.52 µM against HDAC8. Among the four synthesized indoles, only the hydroxamic acid **30** showed favorable IC_50_ values. Notably, **30** is an isomer of **35**, with the hydroxamic acid group in the meta position relative to the boron atom, compared to the para position in **35**. This indicates that the position of the hydroxamic acid exerts a significant influence on inhibitory activity.

Further modification of the capping unit, as in Givinostat, combined with linker-length optimization guided by the promising candidates **23** and **30**, could subsequently enhance the inhibitory activity of these compounds.

## 4. Materials and Methods

^1^H, ^13^C, and ^11^B NMR spectra were acquired at room temperature on a Bruker DRX 500 spectrometer (Bruker BioSpin GmbH, Ettlingen, Germany) at the NMR Department, Technical University of Darmstadt. Proton chemical shifts are expressed as parts per million (ppm, δ scale) and are referenced to residual solvent (1H: CDCl_3_, δ = 7.26; DMSO-d_6_, δ = 2.50; MeOD, δ = 3.31; ^13^C: CDCl_3_, δ = 77.16; DMSO-d_6_, δ = 39.52; MeOD, δ = 49.00). ^11^B NMR spectra were recorded at 160 MHz. Coupling constants (J) are given in Hertz (Hz). Compound purity and reaction progress were monitored by TLC on aluminum-backed silica gel 60 F_254_ plates (Sigma Aldrich, Steinheim, Germany).

Electrospray ionization mass spectrometry (ESI-MS) and HPLC spectra were obtained from a 6100 Agilent Single Quad LC/MS System (Agilent Technologies, Santa Clara, CA, USA), equipped with a Sunfire-RP (C-18, 2.1 × 100 mm, 3–5 μm) column. Eluent A comprising 0.1% (*v*/*v*) aq. trifluoroacetic acid (LC-MS-grade, Sigma Aldrich, Steinheim, Germany) and eluent B comprising MeCN (LC-MS-grade, Thermo Fisher Scientific, Dreieich, Germany) formed the eluent system.

Enzymatic activity assays for compound screening and dose–response analysis were performed as previously described [40]. In brief, compounds were screened at 35 µM using 1 nM HDAC4 and 10 nM HDAC8, respectively. For dose–response curves, serial inhibitor dilutions were incubated with 40 nM HDAC1 (BPS Bioscience, San Diego, CA, USA), 1 nM HDAC4, or 10 nM HDAC8. Reactions were initiated with 20 µM Boc-Lys(Tfa)-AMC (HDAC4/8) or 50 µM Boc-Lys(Ac)-AMC (HDAC1) and terminated with 0.4 mg/mL trypsin (Roth, Karlsruhe, Germany) and 1.7 µM SATFMK (HDAC4/8) or 4.2 µM SAHA (HDAC1). All IC_50_ determinations were performed in triplicate, and the results are expressed as mean values ± standard deviation (SD). HDAC4 and HDAC8 were produced according to previously reported protocols [40,41]. HDAC1 was purchased from BPS Bioscience (San Diego, CA, USA). Boc-Lys(Tfa)-AMC and trypsin were purchased from Bachem (Bubendorf, Switzerland) and AppliChem (Darmstadt, Germany), respectively.

## Figures and Tables

**Table 1:** BN-naphthalene-derived HDAC inhibitors bearing a 1,2,3-triazole unit.

Entry	Product	Yield (%)
**8**	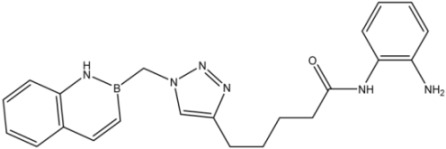	20
**9**	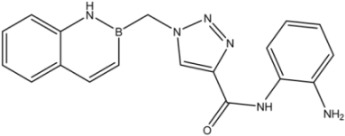	56
**10**	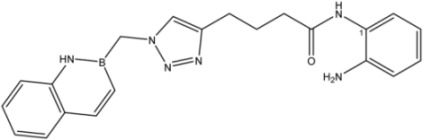	45
**11**	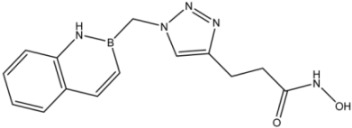	27
**12**	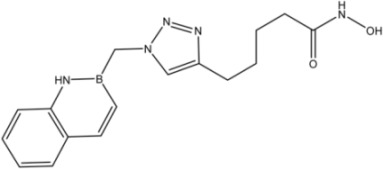	39
**13**	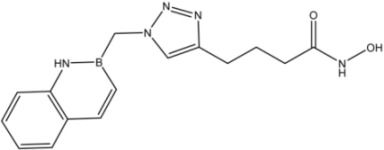	48
**14**	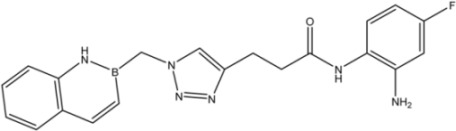	9
**15**	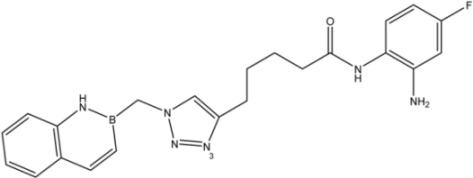	34

**Table 2:** Synthesized compounds with a 2-Carboxylatomethyl-2,1-borazaronaphthalene unit.

Entry	Product	Yield (%)
**16d**	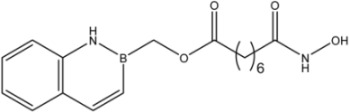	40
**17**	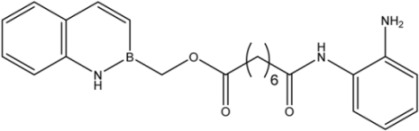	20
**18**	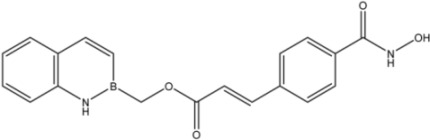	27
**19**	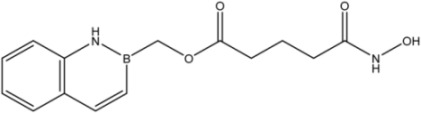	9
**20**	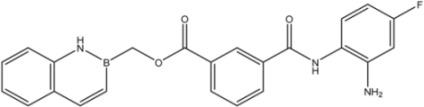	35
**21**	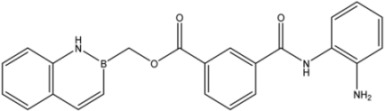	56
**22**	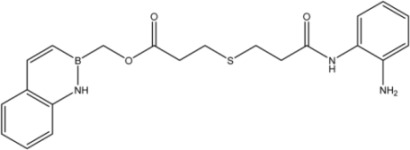	23
**23**	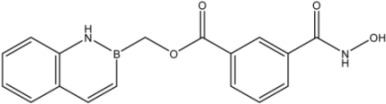	21

**Table 3:** Products obtained from reactions of azabrine building block **6** with amines.

Entry	Product	Yield (%)
**24**	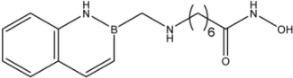	35
**25**	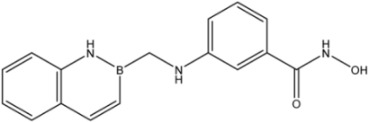	76
**26**	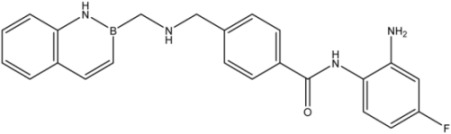	56
**27**	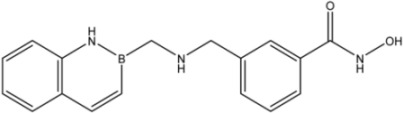	14

**Table 4:** BN-indole-based compounds.

Entry	Product	Yield (%)
**30**	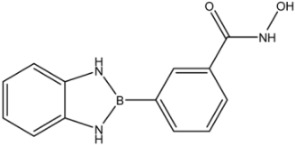	61
**31; 32**	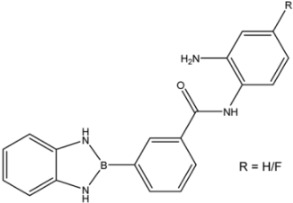	68 (R = H) 60 (R = F)
**35**	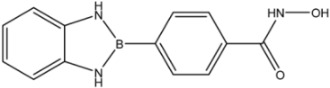	52

**Table 5:** IC_50_ values [µM] of BN-naphthalene-based HDAC inhibitors in enzymatic assays.

Entry	Structure	IC_50_ (HDAC1)	IC_50_ (HDAC4)	IC_50_ (HDAC8)
	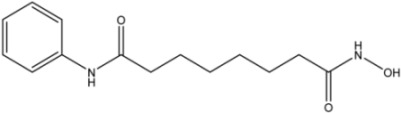 Vorinostat	0.39 [33]	>10	0.66
	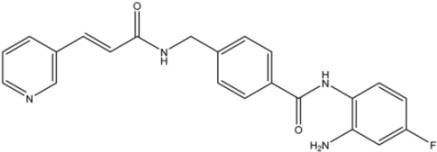 Tucidinostat	0.095 [34]	-	-
	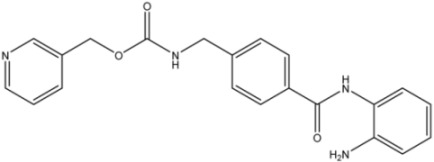 Entinostat	0.46 [35]	-	>100 [36]
**9**	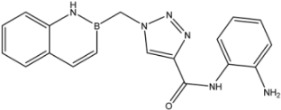	>35	16.8 ± 1.6	20 ± 3
**10**	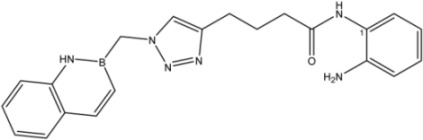	>35	8.08 ± 1.59	19 ± 5
**12**	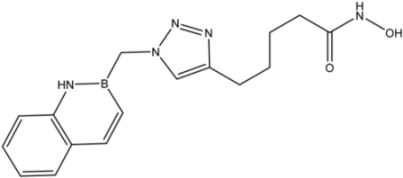	>35	13.8 ± 4.3	6.2 ± 3.4
**13**	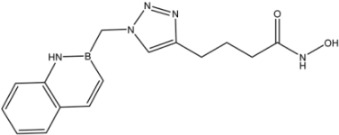	>35	23.1 ± 4.9	3.3 ± 1.7
**16d**	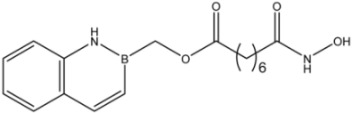	>35	14.7 ± 1.7	>35
**17**	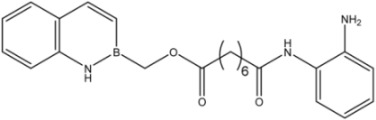	8.2 ± 2.5	16.5 ± 1.0	15 ± 3
**18**	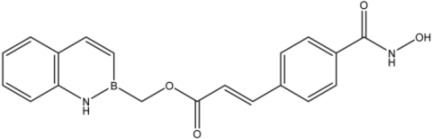	8.2 ± 1.8	>35	0.52 ± 0.11
**23**	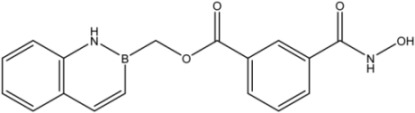	1.88 ± 0.11	7.3 ± 1.7	0.44 ± 0.07
**24**	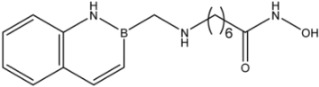	9.3 ± 2.3	>35	>35
**25**	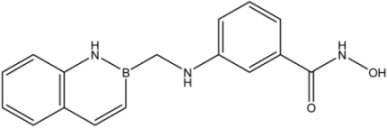	>35	17 ± 3	>35

**Table 6:** IC_50_ values [µM] of BN-indole-based HDAC inhibitors in enzymatic assays.

Entry	Structure	IC_50_ (HDAC1)	IC_50_ (HDAC4)	IC_50_ (HDAC8)
**30**	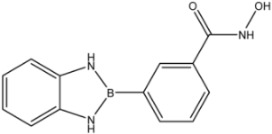	0.975 ± 0.394	1.35 ± 0.18	0.20 ± 0.02
**31; 32**	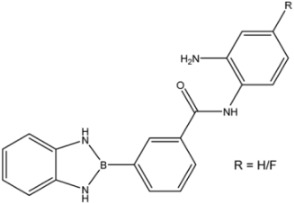	>35	>35	>35
**35**	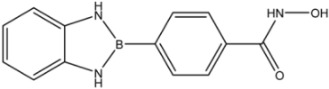	>35	>35	>35

## Data Availability

The original contributions presented in this study are included in the article/Supplementary Materials. Further inquiries can be directed to the corresponding author.

## Supplementary Materials

The supporting information can be downloaded at https://www.mdpi.com/article/10.3390/molecules30194017/s1. Experimental Part: General procedure for linker synthesis; General procedure for the copper(I)-catalyzed azide-alkyne cycloaddition (CuAAC); General procedure for deprotection of benzyl-protecting group; General procedure for the coupling of carboxylic acids with azaborine 6; General procedure for coupling of 6 with amines; General procedure for the synthesis of BN-indoles; NMR-Spectra; Dose-response curves.
