# Supplementary material for: Novel Azaborine-Based Inhibitors of Histone Deacetylases (HDACs)
Source: Molecules. 2025 Oct 8;30(19):4017. doi: 10.3390/molecules30194017 (PMC12525784; doi:10.3390/molecules30194017)
Supplement: Supplementary file 1 [file molecules-30-04017-s001.zip › molecules-3887493-supplementary.pdf]

Supporting Information

Novel Azaborine-Based Inhibitors of Histone Deacetylases (HDACs)

Martin Behringer, Markus Schweipert, Enna E. Peters, Aleksandra Kopranovic and Franz-Josef Meyer-Almes *

Department of Chemical Engineering and Biotechnology, University of Applied Sciences Darmstadt,
Haardtring 100, 64295 Darmstadt, Germany; martin.behringer@h-da.de (M.B.); markus.schweipert@h-da.de (M.S.); ennaemilia.peters@stud.h-da.de (E.E.P.); aleksandra.kopranovic@h-da.de (A.K.)
* Correspondence: franz-josef.meyer-almes@h-da.de; Tel.: +49-6151-53368406

Table of contents

1. Experimental Part 2
General procedure for linker synthesis 3
General procedure for the copper(I)-catalyzed azide-alkyne cycloaddition (CuAAC) 3
General procedure for deprotection of benzyl-protecting group 3
General procedure for the coupling of carboxylic acids with azaborine **6** 7
General procedure for coupling of **6** with amines 11
General procedure for the synthesis of BN-indoles 12
2. NMR-Spectra 16
3. Dose-response curves 59

1. Experimental Part

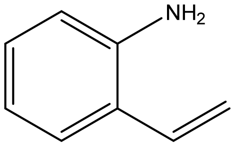

2-Vinylaniline (5). A 100 mL oven-dried Schlenk flask equipped with a magnetic stir bar, a short-path distillation head, and a pre-weighed receiving flask was evacuated and backfilled with argon. 2-Aminophenethyl alcohol (1) (15.0 g, 109 mmol, 1.0 Eq) and potassium hydroxide pellets (6.1 g, 109 mmol, 1 Eq) were then added to the flask. The apparatus was purged and refilled with argon three times, evacuated, and subsequently heated to 180 °C. Upon melting of the potassium hydroxide, the reaction mixture gradually changed in color from brown/purple to green. Continued heating under reduced pressure (1.7 mbar) resulted in the distillation of the target compound **5** as a colorless liquid (83–90 °C) into the pre-weighed receiving flask. The flask was sealed under argon with a septum and stored at –20 °C, affording **5** in 63% yield (6.2 g, 52.0 mmol).

^{1}H-NMR (300 MHz, $CDCl_3$) δ δ 7.37 (dd, *J* = 7.6, 1.6 Hz, 1H), 7.16 (td, *J* = 7.6, 1.6 Hz, 1H), 6.91–6.77 (m, 2H), 6.73 (dd, *J* = 8.0, 1.2 Hz, 1H), 5.70 (dd, *J* = 17.4, 1.5 Hz, 1H), 5.39 (dd, *J* = 11.0, 1.5 Hz, 1H), 3.80 (s, 2H)

^{13}C-NMR (300 MHz, $CDCl_3$) δ 143.8, 132.8, 128.8, 127.3, 124.1, 119.0, 116.2, 115.7

MS (ESI+) m/z calcd. for $C_8H_{10}N$ $[M+H]^+$ 120.07 m/z, found: 120.1

HPLC (5–95% solvent B, 12 min) t_R = 3.864 min

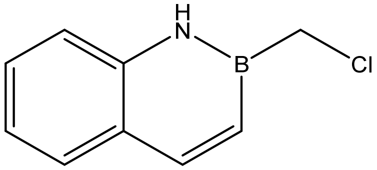

2-(Chloromethyl)-2,1-borazaronaphthalene (6). The compound was obtained as a beige solid. Potassium chloromethyl trifluoroborate (2.50 g, 16 mmol) was placed in an oven-dried Schlenk flask, which was then evacuated and purged with argon three times. Anhydrous cyclopentyl methyl ether (CPME, 10 mL, 0.5 M) and 2-aminostyrene 1 (24 mmol, 1.5 Eq) were added by syringe under argon, followed by triethylamine (2.43 g, 24 mmol) and silicon tetrachloride (849.5 mg, 5 mmol, 1.0 Eq). The resulting mixture was stirred vigorously at 40 °C for 18 h. After completion, the reaction mixture was diluted with n-heptane (50 mL), filtered through a short plug of silica, and washed with *n*-hexane/CH_2Cl_2 (4:1, 80 mL). The solvents were removed under reduced pressure, and the crude product was purified by column chromatography (cyclohexane/ethyl acetate, 5:1) to afford the desired compound as a light yellow solid (56.5%, 1.6 g, 9.0 mmol).

^{1}H NMR (300 MHz, $CDCl_3$) δ 8.07 (d, *J* = 11.5 Hz, 1H), 7.75–7.63 (m, 1H), 7.59–7.46 (m, 1H), 7.37 (d, *J* = 8.1 Hz, 1H), 7.32–7.22 (m, 1H), 6.79 (dd, *J* = 11.5, 2.0 Hz, 1H), 3.73 (s, 2H)

^{13}C NMR (75 MHz, $CDCl_3$) δ 145.5, 145.5, 139.6, 129.5, 128.6, 125.6, 121.4, 118.3

^{11}B NMR (96 MHz, $CDCl_3$) δ 34.59

MS (ESI+) m/z calcd. for $C_9H_{10}BClN$ $[M+H]^+$ 178.05 m/z, found: 178.1

HPLC (5–95% solvent B) t_R = 8.747 min

General procedure for linker synthesis

The following procedure was used to couple suberic acid monomethyl ester, various organic acids, or other substrates such as alkyne acids with THP-protected or Benzyl-protected hydroxamic acid:

The acid (1.0 Eq) was dissolved in acetonitrile (25 mL). *O*-(tetrahydro-2*H*-pyran-2-yl)hydroxylamine (1.2 Eq) or another corresponding amine e.g. (4-Fluoro)-1,2-phenylenediamine, HBTU (1.5 Eq), and DIPEA (2.0 Eq) were added, and the reaction mixture was stirred overnight at room temperature. The crude product was purified by extraction with ethyl acetate, followed by final purification via column chromatography (cyclohexane/ethyl acetate, 5:1 to 1:1).

General procedure for the copper(I)-catalyzed azide-alkyne cycloaddition (CuAAC)

Alkynoic acids were coupled with THP-protected hydroxamic acid to prevent hydrogenation of the triazole ring. Deprotection was achieved by treating the compound with 3.0 eq of 1 M aqueous HCl and stirring overnight. The product was extracted with ethyl acetate and purified by column chromatography using cyclohexane/ethyl acetate or dichloromethane/methanol as the eluent.

Azaborine-N_3 **7** (1.6 Eq) and the corresponding alkyne (1.0 Eq) were stirred with $CuSO_4 \bullet 5H_2O$ (1.6 Eq) and a freshly prepared solution of Na-Ascorbate (3.2 Eq) in a mixture of acetonitrile and water (1:1) at 40°C for 5 h. Upon completion of the reaction, the mixture was extracted with ethyl acetate, and the combined organic phases were dried over anhydrous sodium sulfate. After filtration, the filtrate was concentrated under reduced pressure, and the resulting crude product was purified by column chromatography and preparative thin-layer chromatography (TLC) employing a cyclohexane/ethyl acetate solvent system.

General procedure for deprotection of benzyl-protecting group

The protected molecule was dissolved in MeOH/EtOAc (1:1), 25 mL/mmol and 0.2 Eq Pd/C (10%) was added under argon atmosphere. Hydrogen gas was introduced to the flask for approximately 5 minutes, then the mixture was stirred over hydrogen for 3 h. Afterwards it was filtered, and the solvent was removed under reduced pressure. The compounds were purified via column chromatography using DCM/MeOH (1–10%). In the case of hydroxamic acids, a mixture of DCM/MeOH/acetic acid (90/10/0.1%) was used.

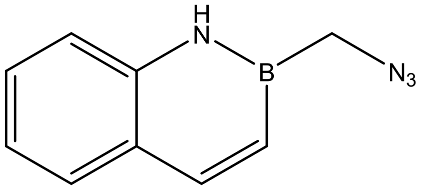

2-(Azidomethyl)-1,2-dihydrobenzo[*e*][1,2]azaborinine (7). To a round bottom flask with stir bar was successively added **1** (600 mg, 2.71 mmol, 1 Eq) and sodium azide (221 mg, 3.25 mmol, 1.2 Eq). Anhyd. Acetonitrile (30 mL) was added, and the reaction was heated to reflux for 10 h. The next day, 110 mg NaN_3 and 30 mL acetonitrile were added and stirred for an additional 3 h. The mixture was filtered over Celite and rinsed with cyclohexane / ethyl acetate (2:1) and evaporated 246 mg (49.3 %,1.3 mmol) of a white powder was obtained as product and stored in a freezer under argon.

^{1}H NMR (500 MHz, DMSO-d_6) δ 8.04 (dd, *J* = 46.9, 11.6 Hz, 1H), 7.83 – 7.54 (m, 1H), 7.49–7.29 (m, 1H), 7.28–7.11 (m, 1H), 7.04 (ddd, *J* = 26.5, 19.7, 7.5 Hz, 1H), 6.94–6.72 (m, 1H), 6.36 (ddd, *J* = 93.7, 11.8, 2.1 Hz, 1H), 4.56 (s, 1H), 3.61 (s, 2H).

^{13}C NMR (75 MHz, $CDCl_3$) δ 148.0, 147.6, 140.8, 129.3, 129.3, 120.0, 117.3, 117.2, 29.7

HPLC (5–95% solvent B) t_R = 6.802 min

^{11}B NMR (160 MHz, $CDCl_3$) δ 28.62

IR (ATR): 3369 cm^{-1}; 2097 cm^{-1}; 1608 cm^{-1}; 1562 cm^{-1}; 1435 cm^{-1}; 1389 cm^{-1}; 1266 cm^{-1}; 1127 cm^{-1}; 1078 cm^{-1}

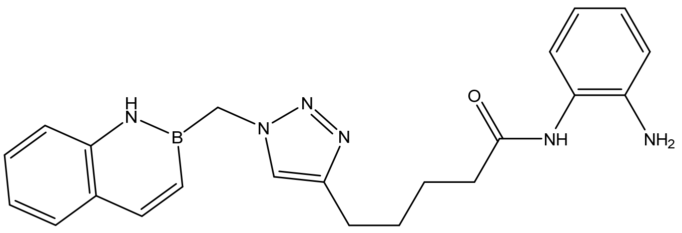

***N*-(2-aminophenyl)-5-(1-(benzo[*e*][1,2]azaborinin-2(1*H*)-ylmethyl)-1*H*-1,2,3-triazol-4yl)-pentanamide (8).** The title compound was obtained as light yellow solid in 20% yield (145 mg, 0.4 mmol).

^{1}H NMR (500 MHz, DMSO-d_6) δ 8.64–8.63 (m, 4H), 8.16–7.57 (m, 4H), 7.48–7.06 (m, 3H), 6.90–6.47 (m, 4H), 6.25 (d, *J* = 11.7 Hz, 1H), 5.62–5.13 (m, 3H), 4.77 (d, 2 H), 2.68 (s, 1H), 2.41 (s, 1H)

^{13}C NMR (126 MHz, DMSO-d_6) δ 146.4, 142.4, 129.1, 128.3, 123.3, 121.3, 120.5, 119.2, 118.9, 117.5, 111.8, 101.4, 57.4, 37.6, 36.0, 34.8, 34.7, 30.6

^{11}B NMR (160 MHz, DMSO-d_6) δ 28.79

MS (ESI+) m/z calcd. for $C_{22}H_{25}BN_6ONa$ $[M+Na]^+$ 423.22, found 423.1

HPLC (5–95% solvent B) t_R = 8.780 min

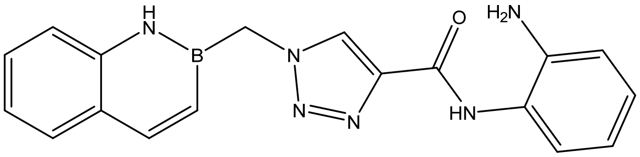

***N*-(2-aminophenyl)-1-(benzo[*e*][1,2]azaborinin-2(1*H*)-ylmethyl)-1*H*-1,2,3-triazole-4-carboxamide (9).** The titled compound was received as a light yellow solid. Yield: 56% (242 mg, 0.7 mmol).

^{1}H NMR (500 MHz, $CDCl_3$) δ 8.03 (d, *J* = 11.8 Hz, 1H), 7.96 (d, *J* = 5.5 Hz, 1H), 7.94–7.87 (m, 1H), 7.59 (dd, *J* = 7.9, 1.5 Hz, 1H), 7.57–7.49 (m, 2H), 7.43–7.37 (m, 1H), 7.35 (dq, *J* = 3.4, 1.8 Hz, 1H), 7.33 (d, *J* = 4.2 Hz, 1H), 6.91–6.85 (m, 1H), 6.62 (dd, *J* = 11.9, 2.2 Hz, 1H), 6.51 (td, *J* = 12.3, 2.3 Hz, 1H), 6.34 (dd, *J* = 11.9, 2.2 Hz, 1H), 5.32 (s, 1H), 4.39 (ddd, *J* = 11.3, 6.0, 3.1 Hz, 1H), 3.85 (s, 1H)

^{13}C NMR (126 MHz, $CDCl_3$) δ 148.0, 147.5, 147.5, 141.0, 130.9, 129.3, 128.8, 128.4, 124.0, 123.7, 123.6, 119.6, 119.5, 117.3, 117.2, 117.1, 117.0, 53.5

MS (ESI+) m/z calcd. for $C_{18}H_{18}BN_6O$ $[M+H]^+$ 345.16, found 345.3

HPLC (5–95% solvent B)) t_R = 6.827 min

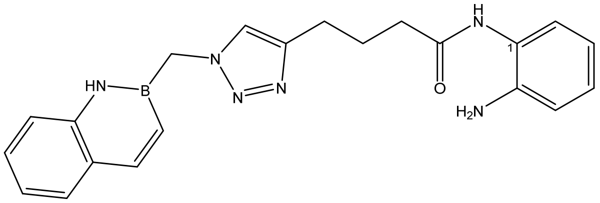

***N*-(2-aminophenyl)-4-(1-(benzo[*c*][1,2]azaborinin-1(2*H*)-ylmethyl)-1*H*-1,2,3-triazol-4-yl)butanamide (10).** The titled compound was received as a white solid in 45% yield (153 mg, 0.4 mmol).

^{1}H NMR (500 MHz, $CDCl_3$) δ 8.02 (d, *J* = 11.9 Hz, 1H), 7.90 (d, *J* = 11.9 Hz, 1H), 7.65–7.49 (m, 2H), 7.37 (dddd, *J* = 21.7, 8.4, 7.2, 1.5 Hz, 2H), 7.17–7.02 (m, 2H), 6.89 (s, 1H), 6.87 (t, *J* = 12.5 Hz, 1H), 6.62 (dd, *J* = 11.8, 2.2 Hz, 1H), 6.33 (dd, *J* = 11.9, 2.1 Hz, 1H), 4.32–4.19 (m, 1H), 4.14 (qd, *J* = 6.9, 4.6 Hz, 1H), 2.91–2.81 (m, 4H), 1.69 (ddt, *J* = 33.5, 13.7, 6.2 Hz, 2H)

^{13}C NMR (126 MHz, $CDCl_3$) δ 148.0, 147.5, 140.8, 129.3, 128.4, 123.8, 120.0, 119.7, 117.3, 117.10, 29.7, 29.4, 14.1

MS (ESI+) m/z calc. for $C_{21}H_{24}BN_6O$ $[M+H]^+$, 387.20 found 387.2

HPLC (5–95% solvent B)) t_R = 6.847 min

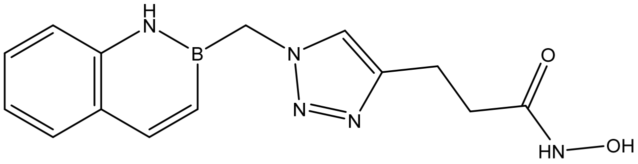

3-(1-(Benzo[*e*][1,2]azaborinin-2(1*H*)-ylmethyl)-1*H*-1,2,3-triazol-4-yl)-*N*-hydroxypropanamide (11). The titled compound was received as a white powder. Yield: 54% (312 mg, 1.1 mmol).

^{1}H NMR (500 MHz, DMSO-d_6) δ 8.64 (s, 1H), 8.00 (s, 1H), 7.76 (d, *J* = 11.8 Hz, 1H), 7.66 (s, 1H), 7.55 (s, 1H), 7.43 (d, *J* = 7.6 Hz, 1H), 7.24 (td, *J* = 8.8, 6.9 Hz, 1H), 6.91 (ddd, *J* = 8.1, 6.3, 1.9 Hz, 1H), 6.53 (s, 1H), 6.36 (d, *J* = 4.7 Hz, 1H), 6.25 (dd, *J* = 12.0, 1.8 Hz, 1H), 2.90 (d, *J* = 7.8 Hz, 2H), 2.75 (t, *J* = 6.1 Hz, 2H)

^{13}C NMR (126 MHz, DMSO-d_6) δ 146.4, 142.4, 129.1, 128.3, 123.3, 118.8, 117.5, 40.6, 40.4, 40.2, 40.1, 39.9, 39.7, 39.6

^{11}B NMR (160 MHz, DMSO-d_6) δ 29.25

MS (ESI+) m/z calcd. for $C_{14}H_{17}BN_5O_2$ $[M+H]^+$ 298.14, found 298.3

HPLC (20–90% solvent B, 12 min) t_R = 5.578 min

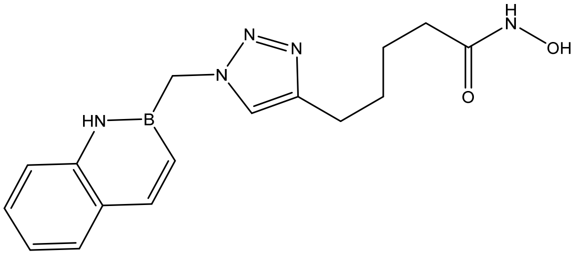

5-(1-(Benzo[*e*][1,2]azaborinin-2(1*H*)-ylmethyl)-1*H*-1,2,3-triazol-4-yl)-*N*-hydroxypentanamide (12). The titled compound was received as a light yellow solid in 39% yield (213 mg, 0.7 mmol).

^{1}H NMR (300 MHz, $CDCl_3$) δ 8.02 (d, *J* = 11.9 Hz, 1H), 7.90 (d, *J* = 11.9 Hz, 1H), 7.63–7.47 (m, 1H), 7.37 (dddd, *J* = 12.1, 8.3, 7.1, 1.6 Hz, 2H), 7.19–7.00 (m, 2H), 6.84 (s, 1H), 6.62 (dd, *J* = 11.9, 2.2 Hz, 2H), 6.34 (td, *J* = 11.4, 2.2 Hz, 1H), 4.01 (s, 1H), 1.29 m, 4H), 0.98 (m, 4H)

^{13}C NMR (75 MHz, $CDCl_3$) δ 140.8, 131.4, 124.0, 130.7, 129.0, 124.2, 123.1, 118.7, 77.5, 77.0, 76.6, 31.3, 29.9

^{11}B NMR (160 MHz, $CDCl_3$) δ 28.27

MS (ESI+) m/z calcd. for $C_{16}H_{21}BN_5O_2$ $[M+H]^+$ 325.17, found 325.2

HPLC (5–100% Eluent B) t_R = 6.693 min

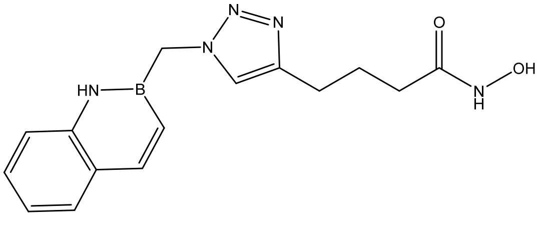

4-(1-(Benzo[*e*][1,2]azaborinin-2(1*H*)-ylmethyl)-1*H*-1,2,3-triazol-4-yl)-*N*-hydroxybutanamide (13). The titled compound was received as a white solid in 48% yield (425 mg, 1.4 mmol).

^{1}H NMR (300 MHz, $CDCl_3$) δ 7.96 (dd, *J* = 36.5, 12.0 Hz, 1H), 7.63–7.47 (m, 1H), 7.37 (dddd, *J* = 11.7, 8.3, 7.1, 1.5 Hz, 1H), 7.19–7.13 (m, 2H), 7.13–7.00 (m, 2H), 6.83 (s, 1H), 6.56 (ddd, *J* = 30.4, 12.0, 2.2 Hz, 2H), 6.32 (dd, *J* = 11.9, 2.2 Hz, 2H), 3.85 (s, 2H), 1.49–1.31 (m, 4H)

^{13}C NMR (75 MHz, $CDCl_3$) δ 140.8, 124.0, 135.5, 139.5, 129.5, 125.8, 132.7, 124.7, 124.0, 118.6, 112.2, 32.1, 31.3, 25.8

^{11}B NMR (160 MHz, $CDCl_3$) δ 28.46

MS (ESI+) m/z calcd. for $C_{15}H_{19}BN_5O_2$ $[M+H]^+$ 312.16, found 312.2

HPLC (5–95% Eluent B) t_R = 6.704 min

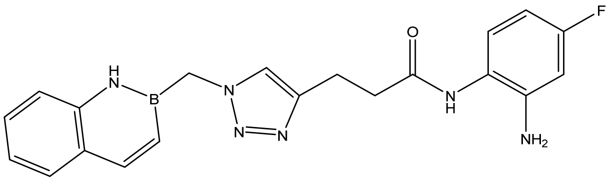

***N*-(2-amino-4-fluorophenyl)-3-(1-(benzo[*e*][1,2]azaborinin-2(1*H*)-ylmethyl)-1*H*-1,2,3-triazol-4-yl)propenamide (14).** The titled compound was received as a light brown solid in 9% yield (88 mg, 0.2 mmol).

^{1}H NMR (500 MHz, DMSO-d_6) δ 9.45–9.41 (m, 1H), 8.68 (s, 1H), 7.99 (d, *J* = 11.9 Hz, 1H), 7.78 (d, *J* = 11.9 Hz, 1H), 7.70 (s, 1H), 7.57 (dd, *J* = 7.8, 1.5 Hz, 1H), 7.43 (dt, *J* = 8.3, 1.6 Hz, 2H), 7.36 (ddd, *J* = 8.2, 6.9, 1.5 Hz, 1H), 7.29–7.22 (m, 2H), 7.04 (td, *J* = 7.5, 1.2 Hz, 1H), 6.92 (ddd, *J* = 8.1, 5.4, 2.9 Hz, 1H), 6.47 (dd, *J* = 11.8, 2.1 Hz, 1H), 6.30 (dd, *J* = 11.8, 2.0 Hz, 1H), 3.43 (t, 2H), 2.51 (t, *J* = 1.8 Hz, 2H)

^{13}C NMR (126 MHz, DMSO-d_6) δ 147.9, 146.5, 142.5, 142.1, 129.4, 129.2, 128.6, 128.3, 123.8, 123.3, 119.8, 118.9, 118.1, 117.56, 40.5, 40.1, 40.0, 39.7, 39.5

^{11}B NMR (160 MHz, DMSO-d$_6$) δ 28.61

MS (ESI+) m/z calcd. for $C_{20}H_{20}BFN_6O$ $[M+Na]^+$ 390.18, found 390.1

HPLC (5–100% Eluent B) t_R = 6.821 min

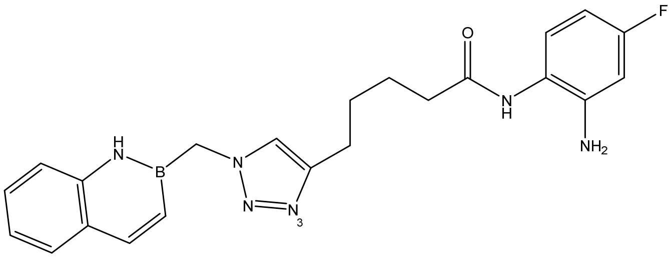

***N*-(2-amino-4-fluorophenyl)-5-(1-(benzo[*e*][1,2]azaborinin-2(1*H*)-ylmethyl)-1*H*-1,2,3-triazol-4-yl)pentanamide (15).** The titled compound was received as a light brown solid in 34% yield (301 mg, 0.7 mmol).

^1H NMR (500 MHz, DMSO-d$_6$) δ 9.41 (s, 1H), 8.65 (s, 1H), 7.99 (dd, *J* = 11.7, 3.0 Hz, 1H), 7.77 (dd, *J* = 11.7, 2.7 Hz, 1H), 7.67 (s, 1H), 7.57 (t, *J* = 5.4 Hz, 1H), 7.42 (dd, *J* = 14.8, 8.0 Hz, 2H), 7.36 (d, *J* = 7.5 Hz, 1H), 7.24 (s, 2H), 7.04 (td, *J* = 7.7, 3.1 Hz, 1H), 6.91 (tt, *J* = 5.3, 2.8 Hz, 1H), 6.45 (d, *J* = 11.8 Hz, 1H), 6.26 (d, *J* = 11.9 Hz, 1H), 1.23 (m, 8H)

^{13}C NMR (126 MHz, DMSO-d$_6$) δ 147.8, 147.2, 146.4, 142.5, 142.1, 129.3, 129.1, 128.6, 128.3, 123.8, 123.3, 119.8, 118.8, 118.1, 117.8, 117.5, 40.3, 40.3, 39.8, 39.7, 39.5

^{11}B NMR (160 MHz, DMSO-d$_6$) δ 28.70

MS (ESI+) m/z calcd. for $C_{22}H_{25}BFN_6O$ $[M+H]^+$ 419.21, found 419.1

HPLC (5–100% Eluent B) t_R = 6.825 min

General procedure for the coupling of carboxylic acids with azaborine **6**

A solution of 2-chloromethyl-2,1-borazaronaphthalene **6** (1.0 Eq) in acetonitrile (1 mL/mmol) was treated with the corresponding carboxylic acid (1.2 Eq), followed by the addition of Cs_2CO_3 (1.0 Eq). The mixture was heated at 70 °C and stirred for approximately 12 h, until complete consumption of **6**, confirmed by TLC. After cooling to room temperature, the reaction mixture was filtered through Celite, and the solvent was removed under reduced pressure. The crude product was purified by column chromatography on silica gel using EtOAc/hexanes as the eluent.

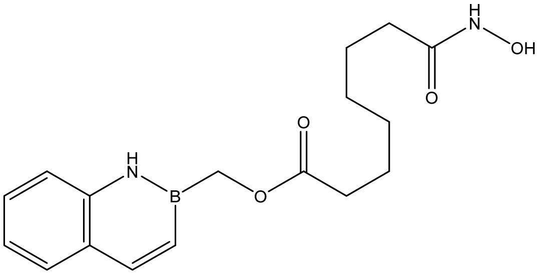

Benzo[*e*][1,2]azaborinin-2(1*H*)-ylmethyl 8-(hydroxyamino)-8-oxooctanoate (16d). The titled compound was received as a light yellow solid in 40% yield (20 mg, 0.1 mmol).

^{1}H NMR (300 MHz, $CDCl_3$) δ 8.05 (d, *J* = 11.6 Hz, 2H), 7.65 (d, *J* = 7.8 Hz, 1H), 7.45 (ddd, *J* = 8.4, 7.0, 1.5 Hz, 1H), 7.32 (d, *J* = 8.1 Hz, 1H), 7.26–7.15 (m, 1H), 6.79 (dd, *J* = 11.5, 1.9 Hz, 1H), 5.32 (s, 1H), 4.46 (s, 2H), 3.52 (s, 1H), 2.49 (t, *J* = 7.5 Hz, 2H), 1.83–1.72 (m, 2H), 1.53–1.42 (m, 2H), 1.28 (s, 4H), 0.89 (q, *J* = 7.3 Hz, 2H)

^{13}C NMR (75 MHz, $CDCl_3$) δ 174.5, 145.4, 139.5, 129.6, 128.4, 125.6, 121.2, 118.1, 53.4, 34.3, 29.7, 28.9, 25.0

MS (ESI+) m/z calcd. for $C_{17}H_{24}BN_2O_4$ $[M+H]^+$ 331.18, found 331.3

HPLC (5–95% Eluent B) t_R = 6.722 min

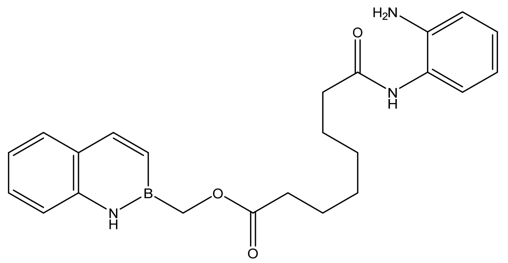

Benzo[*e*][1,2]azaborinin-2(1*H*)-ylmethyl 8-((2-aminophenyl)amino)-8-oxooctanoate (17). The titled compound was received as a light yellow solid in 20% yield (289 mg, 0.7 mmol).

^{1}H NMR (500 MHz, $CDCl_3$) δ 8.02 (d, *J* = 11.9 Hz, 1H), 7.89 (d, *J* = 11.9 Hz, 1H), 7.58 (dd, *J* = 7.9, 1.4 Hz, 1H), 7.51 (dd, *J* = 7.6, 1.5 Hz, 1H), 7.37 (dddd, *J* = 20.6, 8.4, 7.1, 1.5 Hz, 2H), 7.18–7.03 (m, 2H), 6.83 (s, 1H), 6.61 (dd, *J* = 11.8, 2.2 Hz, 1H), 6.32 (dd, *J* = 11.9, 2.2 Hz, 2H), 4.13 (dt, *J* = 10.3, 6.8 Hz, 2H), 2.93–2.80 (m, 2H), 1.65 (p, *J* = 6.8 Hz, 2H), 1.49–1.41 (m, 2H), 1.41–1.22 (m, 2H), 1.07–1.02 (m, 2H), 0.95–0.81 (m, 2H)

^{13}C NMR (126 MHz, $CDCl_3$) δ 148.0, 147.6, 140.8, 140.7, 129.3, 128.7, 128.4, 128.4, 124.0, 123.6, 120.0, 119.7, 117.3, 117.2, 65.0, 34.8, 31.9, 29.3, 28.6, 27.1, 25.9

MS (ESI+) m/z calcd. for $C_{23}H_{29}BN_3O_3$ $[M+H]^+$ 406.22, found 406.3

HPLC (5–100% Eluent B) t_R = 6.821 min

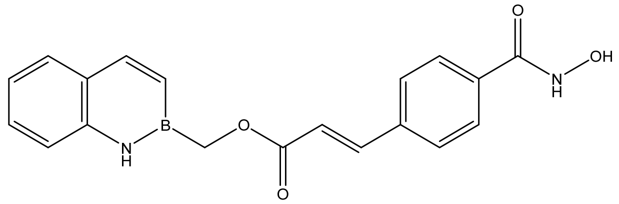

Benzo[*e*][1,2]azaborinin-2(1*H*)-ylmethyl (*E*)-3-(4-(hydroxycarbamoyl)phenyl)acrylate (18). The titled compound was received as a white solid in 27% yield (62 mg, 0.2 mmol).

^{1}H NMR (500 MHz, DMSO-d_6) δ 10.21 (s, 1H), 8.67 (s, 1H), 8.06 (d, J = 11.5 Hz, 1H), 7.84 (s, 2H), 7.83–7.36 (m, 1H), 7.28–7.11 (m, 2H), 7.10–6.84 (m, 2H), 6.78 (dd, J = 11.5, 1.8 Hz, 1H), 6.25 (dd, J = 11.8, 2.1 Hz, 1H), 5.01 (s, 1H), 4.48 (s, 2H).

^{13}C NMR (126 MHz, DMSO-d_6) δ 129.3, 128.4, 119.0, 81.9, 30.6, 26.00, 23.8

MS (ESI+) m/z calcd. for $C_{19}H_{18}BN_2O_4$ $[M+H]^+$ 349.13, found 349.1

HPLC (5–95% Eluent B) t_R = 8.599 min

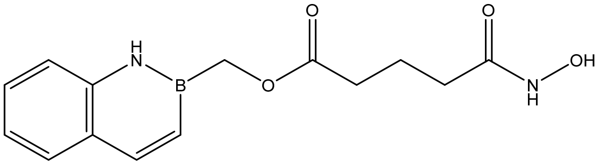

Benzo[*e*][1,2]azaborinin-2(1*H*)-ylmethyl 5-(hydroxyamino)-5-oxopentanoate (19). The titled compound was received as a white solid in 9% yield (46 mg, 0.2 mmol).

^{1}H NMR (500 MHz, DMSO-d$_6$) δ 8.81 (s, 1H), 7.76 (d, J = 11.9 Hz, 1H), 7.43 (d, J = 7.7 Hz, 1H), 7.28–7.17 (m, 1H), 7.00–6.82 (m, 1H), 6.71–6.46 (m, 1H), 6.40–6.03 (m, 1H), 5.73 (dt, J = 12.1, 6.4 Hz, 1H), 4.97 (d, J = 35.9 Hz, 1H), 4.10 (s, 2H), 1.84–1.02 (m, 6H).

^{13}C NMR (126 MHz, DMSO-d$_6$) δ 146.4, 142.4, 132.8, 129.1, 128.3, 118.8, 117.5, 116.9, 115.9, 115.1, 62.6, 58.6

MS (ESI+) m/z calcd. for $C_{14}H_{18}BN_2O_4$ $[M+H]^+$ 289.13, found 289.0

HPLC (5–95% Eluent B) t_R = 6.812 min

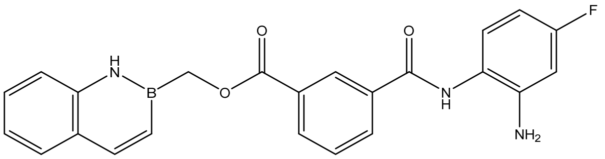

Benzo[*e*][1,2]azaborinin-2(1*H*)-ylmethyl 3-((2-amino-4-fluorophenyl)carbamoyl)-benzoate (20). The titled compound was received as a light brown solid in 35% yield (171 mg, 0.4 mmol).

^{1}H NMR (500 MHz, DMSO-d$_6$) δ 10.30 (s, 2H), 9.85 (s, 1H), 8.69 (s, 1H), 8.65 (s, 1H), 8.28 (d, *J* = 7.7 Hz, 1H), 8.08 (d, *J* = 11.6 Hz, 1H), 7.80–7.72 (m, 1H), 7.68 (q, *J* = 7.4 Hz, 1H), 7.60–7.50 (m, 1H), 7.50–7.37 (m, 1H), 7.28–7.11 (m, 1H), 6.91 (ddd, *J* = 7.9, 6.0, 2.1 Hz, 1H), 6.82 (d, *J* = 11.3 Hz, 1H), 6.57 (dd, *J* = 11.2, 2.9 Hz, 1H), 6.46–6.34 (m, 1H), 6.26 (dd, *J* = 11.9, 1.7 Hz, 1H), 4.66 (s, 1H), 4.62 (d, *J* = 10.6 Hz, 1H)

^{13}C NMR (126 MHz, DMSO-d$_6$) δ 166.8, 165.4, 162.6, 145.5, 142.4, 140.6, 135.5, 132.5, 130.9, 129.7, 129.3, 129.2, 129.1, 128.9, 119.5, 118.8, 117.5, 102.6, 102.0, 101.8, 59.7

MS (ESI+) m/z calcd. for $C_{23}H_{20}BFN_3O_3$ $[M+H]^+$ 416.15, found 416.2

HPLC: (5–95% Eluent B) t_R = 9.531 min

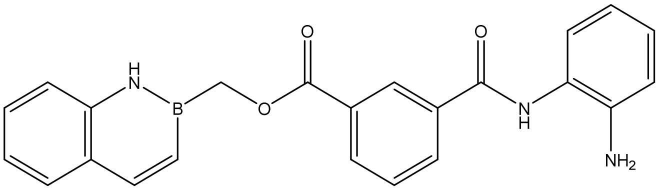

Benzo[*e*][1,2]azaborinin-2(1*H*)-ylmethyl 3-((2-aminophenyl)carbamoyl)benzoate (21). The titled compound was received as a light yellow solid in 56% yield (126 mg, 0.3 mmol).

^{1}H NMR (500 MHz, DMSO-d$_6$) δ 10.30 (s, 1H), 9.91 (s, 1H), 8.74–8.64 (m, 1H), 8.29 (dt, *J* = 7.8, 1.9 Hz, 1H), 8.09 (s, 1H), 8.07 (s, 1H), 7.77 (d, *J* = 11.9 Hz, 1H), 7.74–7.61 (m, 1H), 7.58 (d, *J* = 8.2 Hz, 1H), 7.50–7.40 (m, 1H), 7.40–7.27 (m, 1H), 7.27–7.20 (m, 1H), 7.20–7.14 (m, 1H), 7.12–6.86 (m, 1H), 6.86–6.76 (m,

1H), 6.68–6.58 (m, 1H), 6.26 (dd, J = 11.8, 2.1 Hz, 1H), 4.98 (s, 1H), 4.66 (s, 1H)
13C NMR (126 MHz, DMSO-d6) δ 166.8, 165.1, 146.4, 145.5, 135.6, 132.8, 132.7, 132.5, 130.9, 129.9, 129.7, 129.2, 128.9, 128.3, 127.2, 126.0, 125.4, 123.3, 118.8, 117.5, 116.7, 115.0, 59.8

MS (ESI+) m/z calcd. for C23H21BN3O3 [M+H]+ 398.16, found 398.1

HPLC (5–95% Eluent B) tR = 7.553 min

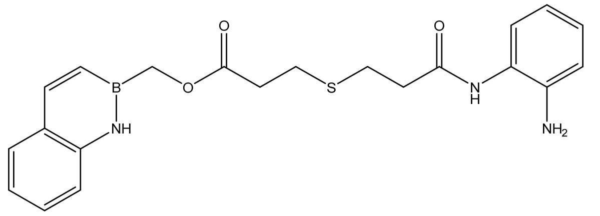

Benzo[*e*][1,2]azaborinin-2(1*H*)-ylmethyl-3-((3-((2-aminophenyl)amino)-3-oxopropyl)-thio)propanoate (22). The titled compound was received as a light brown solid in 23% yield (81 mg, 0.2 mmol).

^{1}H NMR (500 MHz, DMSO-d_6) δ 8.66 (s, 2H), 7.99 (d, *J* = 11.7 Hz, 1H), 7.89 (d, *J* = 12.1 Hz, 1H), 7.77 (d, *J* = 11.9 Hz, 1H), 7.67 (s, 1H), 7.60–7.47 (m, 1H), 7.43 (d, *J* = 7.7 Hz, 1H), 7.37–7.28 (m, 1H), 7.24 (dd, *J* = 6.2, 1.6 Hz, 2H), 7.07–7.01 (m, 1H), 7.01–6.96 (m, 1H), 6.91 (ddd, *J* = 8.1, 6.1, 2.2 Hz, 2H), 6.42 (ddd, *J* = 29.7, 11.8, 2.0 Hz, 1H), 6.26 (dd, *J* = 11.9, 2.0 Hz, 8H)

^{13}C NMR (126 MHz, DMSO-d_6) δ 147.8, 147.2, 146.4, 142.4, 142.0, 129.3, 129.1, 128.6, 128.3, 123.5, 123.2, 119.8, 118.8, 117.5, 114.4, 52.6, 40.2, 39.9, 39.8, 39.7, 39.5

^{11}B NMR (160 MHz, DMSO-d_6) δ 28.81

MS (ESI+) m/z calcd. for $C_{21}H_{25}BN_3O_3S$ $[M+H]^+$ 410.16, found 410.1

HPLC (5–95% Eluent B) t_R = 7.127 min

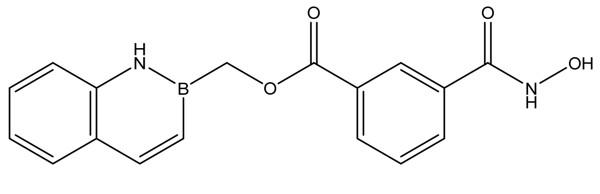

Benzo[*e*][1,2]azaborinin-2(1*H*)-ylmethyl 3-(hydroxycarbamoyl)benzoate (23). This compound was received as a white solid in 21% yield (22 mg, 0.1 mmol).

^{1}H NMR (500 MHz, DMSO-d_6) δ 8.06 (q, J = 10.5 Hz, 1H), 7.76 (d, J = 11.8 Hz, 1H), 7.69 (dt, J = 15.1, 7.6 Hz, 1H), 7.54–7.39 (m, 1H), 7.33 (dd, J = 12.5, 8.3 Hz, 1H), 7.29–7.21 (m, 1H), 7.11–6.85 (m, 1H), 6.55 (ddd, J = 39.6, 16.1, 7.7 Hz, 1H), 6.30 (d, J = 11.7 Hz, 1H), 4.81 (s, 1H), 4.50–4.43 (m, 1H), 4.31 (s, 2H)

^{13}C NMR (126 MHz, $CDCl_3$) δ 144.5, 137.2, 136.3, 133.4, 131.4, 131.3, 129.5, 128.4, 128.7, 126.48, 120.3, 118.8, 117.2, 116.5, 66.0

MS (ESI+) m/z calcd. for $C_{17}H_{16}BN_2O_4$ $[M+H]^+$ 323.11, found 323.0

HPLC (5 – 95% Eluent B) t_R = 7.467 min

General procedure for coupling of **6** with amines

A solution of the corresponding amine (1 mmol) in anhydrous THF (1 mL, 1 M) was stirred at room temperature and treated with 2-chloromethyl-2,1-borazaronaphthalene **6** (1 mmol). Upon completion, water was added, and the aqueous phase was extracted with n-hexane (4 × 5 mL). The combined organic layers were washed with saturated brine, dried over Na_2SO_4, and the solvent was removed under reduced pressure to afford the desired product. Final purification was achieved by column chromatography (cyclohexane/ethyl acetate, 1:1).

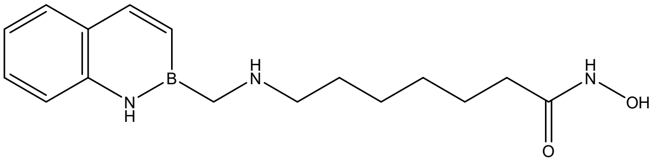

7-((Benzo[*e*][1,2]azaborinin-2(1*H*)-ylmethyl)amino)-*N*-hydroxyheptanamide (24). The compound was received as a light beige solid (Yield: 35%, 131 mg, 0.4 mmol).

^{1}H NMR (300 MHz, DMSO-d$_6$) δ 9.42 (s, 1H), 8.67 (s, 1H), 7.99 (d, *J* = 11.9 Hz, 1H), 7.77 (d, *J* = 11.9 Hz, 1H), 7.68 (s, 1H), 7.58 (d, *J* = 7.8 Hz, 1H), 7.43 (d, *J* = 8.0 Hz, 1H), 7.44–7.18 (m, 4H), 7.09–6.98 (m, 1H), 6.91 (ddd, *J* = 7.9, 5.7, 2.4 Hz, 1H), 6.44 (dd, *J* = 11.7, 2.0 Hz, 1H), 6.25 (dd, *J* = 11.8, 2.0 Hz, 1H), 1.33–1.13 (m, 6H)

^{13}C NMR (75 MHz, DMSO-d$_6$) δ 147.8, 146.4, 142.1, 129.3, 129.1, 128.3, 123.8, 123.3, 119.8, 118.8, 117.5, 40.8, 40.6, 40.3, 40.00, 39.7

MS (ESI+) m/z calcd. for $C_{16}H_{25}BN_3O_2$ $[M+H]^+$ 302.20, found 302.2

HPLC (5–95% Eluent B): t_R = 4.432 min

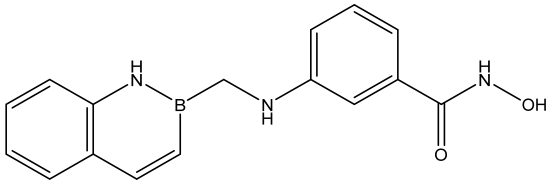

3-((Benzo[*e*][1,2]azaborinin-2(1*H*)-ylmethyl)amino)-*N*-hydroxybenzamide (25). The titled compound was received as a white solid in 76% yield (484 mg, 1.3 mmol).

^{1}H NMR (500 MHz, DMSO-d$_6$) δ 9.44–9.40 (m, 1H), 8.66 (s, 1H), 7.99 (d, *J* = 11.8 Hz, 1H), 7.77 (d, *J* = 11.8 Hz, 1H), 7.68 (s, 1H), 7.57 (dd, *J* = 7.9, 1.5 Hz, 1H), 7.48–7.39 (m, 2H), 7.35 (ddd, *J* = 8.2, 6.9, 1.5 Hz, 1H), 7.29–7.22 (m, 2H), 7.07–6.97 (m, 1H), 6.91 (ddd, *J* = 8.1, 4.9, 3.4 Hz, 1H), 6.46 (dd, *J* = 11.8, 2.1 Hz, 1H), 6.27 (dd, *J* = 11.8, 2.1 Hz, 2H)

^{13}C NMR (126 MHz, DMSO-d$_6$) δ 147.8, 147.0, 146.4, 142.5, 142.1, 130.3, 129.3, 129.1, 128.6, 128.3, 123.8, 119.8, 118.8, 118.1, 117.6, 40.5

^{11}B NMR (160 MHz, DMSO-d$_6$) δ 28.57

MS (ESI+) m/z calcd. for $C_{16}H_{17}BN_3O_2$ $[M+H]^+$ 294.13, found 294.1

HPLC (5–95% Eluent B) t_R = 6.506 min

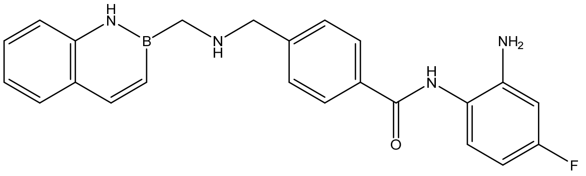

N-(2-amino-4-fluorophenyl)-4-(((benzo[e][1,2]azaborinin-2(1H)-ylmethyl)amino)methyl)-benzamide (26). The titled compound was received as a light yellow solid in 56% yield (269 mg, 0.7 mmol).

^{1}H NMR (500 MHz, DMSO-d$_6$) δ 9.42 (s, 2H), 8.66 (s, 1H), 7.99 (d, *J* = 11.8 Hz, 2H), 7.77 (d, *J* = 11.8 Hz, 1H), 7.57 (d, *J* = 7.8 Hz, 2H), 7.46–7.39 (m, 3H), 7.39–7.32 (m, 2H), 7.24 (q, *J* = 3.7 Hz, 2H), 7.04 (t, *J* = 7.3 Hz, 2H), 6.91 (ddd, *J* = 8.1, 5.2, 3.1 Hz, 1H), 6.46 (dd, *J* = 11.8, 2.1 Hz, 2H), 6.27 (dd, *J* = 11.8, 2.0 Hz, 2H)

^{13}C NMR (126 MHz, DMSO-d$_6$) δ 147.8, 146.4, 142.5, 142.1, 129.4, 129.1, 128.6, 128.3, 123. 8, 123.3, 119.8, 118.9, 118.1, 117.6, 107.2, 103.2, 54.0, 40.5

^{11}B NMR (160 MHz, DMSO-d$_6$) δ 28.45

MS (ESI+) m/z calcd. for $C_{23}H_{23}BFN_4O$ $[M+H]^+$ 401.19, found 401.1

HPLC (5–95% Eluent B) t_R = 0.603 min

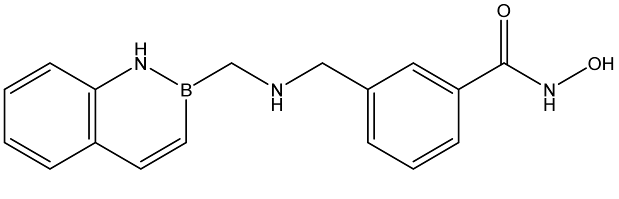

3-(((Benzo[e][1,2]azaborinin-2(1H)-ylmethyl)amino)methyl)-N-hydroxybenzamide (27). The titled compound was received as a light yellow solid in 14% yield (34 mg, 0.1 mmol).

^{1}H NMR (500 MHz, DMSO-d$_6$) δ 9.81 (s, 1H), 8.99 (d, J = 2.8 Hz, 1H), 8.28 (d, J = 2.9 Hz, 1H), 8.26 (d, J = 2.9 Hz, 1H), 8.23 (s, 2H), 7.68 (s, 1H), 6.65 (d, J = 9.3 Hz, 1H), 4.43–4.33 (m, 2H), 4.18 (q, J = 7.1 Hz,2H), 3.37 (s, 2H)

^{13}C NMR (126 MHz, DMSO-d$_6$) δ 170.98, 165.66, 163.87, 147.64, 134.36, 133.24, 130.09, 107.78, 67.74, 60.38, 30.52, 28.99.

MS (ESI+) m/z calcd. for $C_{17}H_{19}BN_3O_2$ $[M+H]^+$ 308.15, found 308.2

HPLC (5–95% Eluent B) t_R =10.503 min

General procedure for the synthesis of BN-indoles

1,2-Phenylendiamine (1 Eq), organotrifluoroborate (1 Eq), and $BF_3{\cdot}NH_2Et$ (3 Eq) were placed in a round-bottom flask equipped with a stir bar. A reflux condenser with a disposable Teflon septum was attached, and the reaction vessel was evacuated and purged with argon three times. A 1:1 mixture of CPME and toluene (2 mL per 1 mmol Amine) was then added, and the reaction mixture was heated to 80 °C. After stirring overnight, the mixture was diluted with 50 mL of saturated $NaHCO_3$ and extracted with EtOAc (2 × 50 mL). The combined organic layers were washed with brine, dried over $MgSO_4$, and concentrated under reduced pressure to afford the azaborine. Final Purification was performed using column chromatography (Cyclohexane / Ethyl acetate 5:1).

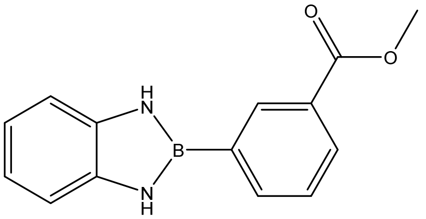

Methyl 3-(1,3-dihydro-2*H*-benzo[*d*][1,3,2]diazaborol-2-yl)benzoate (28). The titled compound was received as a tan solid in 23% yield (606 mg, 2.4 mmol).

^{1}H NMR (500 MHz, DMSO-d_6) δ 9.33 (s, 2H), 8.55 (t, *J* = 1.6 Hz, 1H), 8.17 (dt, *J* = 7.4, 1.4 Hz, 1H), 8.00 (dt, *J* = 7.8, 1.5 Hz, 1H), 7.58 (t, *J* = 7.6 Hz, 1H), 7.08 (dd, *J* = 5.7, 3.3 Hz, 2H), 6.84 (dd, *J* = 5.7, 3.2 Hz, 2H), 3.90 (s, 3H)

^{13}C NMR (126 MHz, DMSO-d_6) δ 167.0, 139.3, 138.6, 137.5, 135.4, 134.5, 133.5, 130.4, 129.8, 128.3, 118.9,111.4, 1114, 52.6.

^{11}B NMR (160 MHz, DMSO-d_6) δ 28.91

HPLC (20–80% Eluent B) t_R = 6.516 min

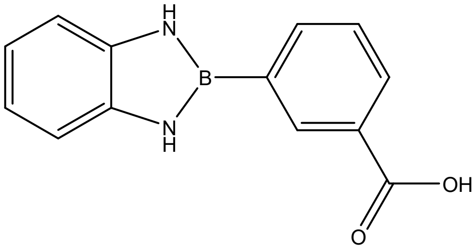

3-(1,3-Dihydro-2*H*-benzo[*d*][1,3,2]diazaborol-2-yl)benzoic acid (29). The titled compound was received as a light yellow solid in 39% yield (48 mg, 0.2 mmol).

^{1}H NMR (500 MHz, DMSO-d_6) δ 8.41 (d, *J* = 1.7 Hz, 1H), 8.22 (s, 3H), 8.03–7.94 (m, 3H), 7.46 (t, *J* = 7.5 Hz, 1H), 7.40–7.25 (m, 1H), 6.49 (dt, *J* = 7.3, 3.6 Hz, 1H), 6.37 (dd, *J* = 5.7, 3.4 Hz, 1H)

^{13}C NMR (126 MHz, DMSO-d_6) δ 168.2, 138.9, 135.6, 135.3, 131.3, 130.3, 128.1, 117.8, 115.1

^{11}B NMR (160 MHz, DMSO-d_6) δ 24.07

HPLC (20-80% Eluent B) t_R = 1.397 min

The coupling of **29** with (4-fluoro)-1,2-phenylenediamine or with the protected hydroxamic acid was carried out according to the general procedure for linker synthesis.

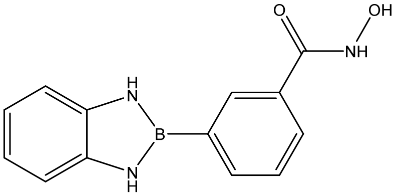

3-(1,3-dihydro-2*H*-benzo[*d*][1,3,2]diazaborol-2-yl)-*N*-hydroxybenzamide (30). The titled compound was received as a white solid in 61% yield (29 mg, 0.1 mmol).

^{1}H NMR (500 MHz, DMSO-d6) δ 8.52 (s, 1H), 8.10 – 7.95 (m, 1H), 7.77–7.57 (m, 2H), 6.91 (dd, J = 11.8, 7.5 Hz, 1H), 6.80 (t, J = 9.2 Hz, 1H), 6.67 (d, J = 7.9 Hz, 1H), 5.76 (s, 1H), 4.83 (d, J = 104.3 Hz, 2H).

^{13}C NMR (126 MHz, DMSO-d6) δ 175.1, 133.3, 129.9, 117.7, 114.7

HPLC (5–95% Eluent B) t_R = 4.897 min

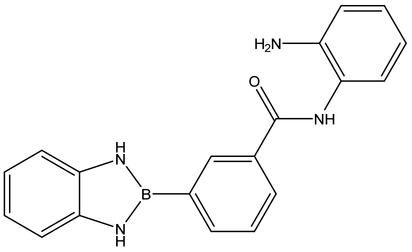

***N*-(2-aminophenyl)-3-(1,3-dihydro-2*H*-benzo[*d*][1,3,2]diazaborol-2-yl)benzamide (31).** The titled compound was received as a light yellow solid in 68% yield (42 mg, 0.1 mmol).

^{1}H NMR (500 MHz, CDCl3) δ 10.65 (dt, *J* = 7.2, 3.7 Hz, 1H), 10.55 (dd, *J* = 5.7, 3.4 Hz, 1H), 8.82 (m, 8H), 7.27 (p, *J* = 1.7 Hz, 2H), 6.82 (s, 1H), 6.81–6.53 (m, 1H), 6.23–6.07 (m, 1H), 5.25 (s, 1H), 4.91 (dd, *J* = 43.2, 6.5 Hz, 1H)

^{13}C NMR (126 MHz, CDCl3) δ 171.0, 138.5, 135.0, 130,9, 126.5, 123.3, 122.4, 121.2, 120.3, 114.8, 118.3, 112.7

HPLC (5–95% Eluent B) t_R = 5.206 min

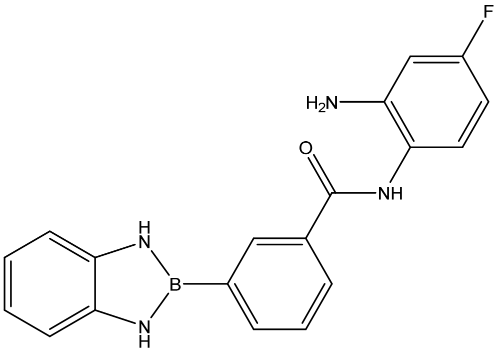

***N*-(2-amino-4-fluorophenyl)-3-(1,3-dihydro-2*H*-benzo[*d*][1,3,2]diazaborol-2-yl)benzamide (32).** The titled compound was received as a light brown solid in 60% yield (39 mg, 0.1 mmol).

^{1}H NMR (500 MHz, DMSO-d6) δ 7.95 (s, 2H), 7.53 (m, 3H), 7.44 (d, *J* = 8.0 Hz, 3H), 7.00 (dt, *J* = 19.6, 7.0 Hz, 5H)

^{13}C NMR (126 MHz, DMSO-d6) δ 155.6, 143.3, 132.0, 132.0, 127.6, 122.3, 121.0, 118.1, 112.6, 105.1

HPLC (5–95% Eluent B) t_R = 4.598 min

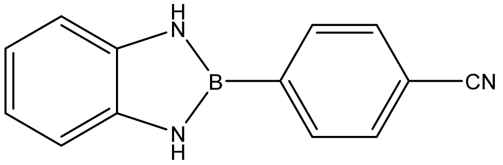

4-(1,3-dihydro-2*H*-benzo[*d*][1,3,2]diazaborol-2-yl)benzonitrile (33). The titled compound was recevied as a tan solid in 38% yield (1004 mg, 4.6 mmol).

^{1}H NMR (500 MHz, DMSO-d_6) δ 9.38 (s, 2H), 8.12–8.07 (m, 2H), 7.91–7.86 (m, 2H), 7.11 (dt, *J* = 7.4, 3.7 Hz, 2H), 6.87 (dd, *J* = 5.7, 3.2 Hz, 2H)

^{13}C NMR (126 MHz, DMSO-d_6) δ 138.6, 137.4, 134.4, 132.0, 119.6, 119.2, 112.1, 111. 7

^{11}B NMR (160 MHz, DMSO-d_6) δ 28.59

HPLC (5–95% Eluent B) t_R = 6.045 min

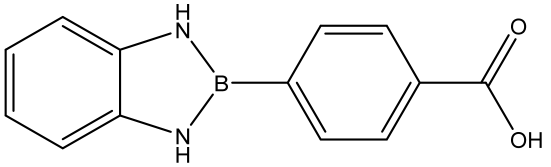

4-(1,3-Dihydro-2*H*-benzo[*d*][1,3,2]diazaborol-2-yl)benzoic acid (34). The titled compound was received as a light brown solid in 93% yield (456 mg, 1.9 mmol).

Compound **33** was dissolved in DMF (15 mL) until complete dissolution was achieved. NaOH (1 M, 6 mL, 10 Eq) was then added, and the mixture was stirred at 80 °C. The solution was acidified and extracted with ethyl acetate. The organic layer was dried over Na_2SO_4 and concentrated under reduced pressure. Purification was carried out by column chromatography using DCM/MeOH (5%).

^{1}H NMR (500 MHz, DMSO-d_6) δ 9.37 (s, 1H), 8.42 (s, 1H), 8.08 (d, *J* = 7.9 Hz, 1H), 7.94 (d, *J* = 7.8 Hz, 1H), 7.87 (dd, *J* = 14.7, 5.3 Hz, 2H), 7.79 (d, *J* = 7.8 Hz, 1H), 7.64 (dd, *J* = 8.6, 2.5 Hz, 1H), 7.44 (s, 1H), 7.10 (dd, *J* = 5.7, 3.2 Hz, 1H), 6.89 (ddt, *J* = 21.2, 5.7, 3.1 Hz, 1H)

^{13}C NMR (126 MHz, DMSO-d_6) δ 137.4, 135.1, 134.7, 134.4, 132.0, 131.6, 131.5, 119.7, 119.5, 119.2, 116.9, 112.9, 111.7

^{11}B NMR (160 MHz, DMSO-d_6) δ 28.00

HPLC (5–95% Eluent B) t_R = 6.819 min

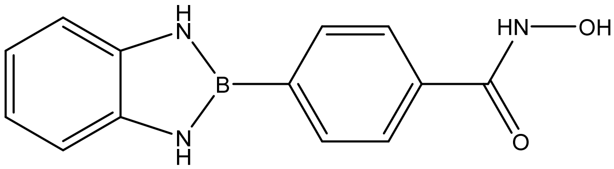

4-(1,3-Dihydro-2*H*-benzo[*d*][1,3,2]diazaborol-2-yl)-*N*-hydroxybenzamide (35). The titled compound was received as a white solid in 52% yield (105 mg, 0.4 mmol).

^{1}H NMR (500 MHz, $CDCl_3$) δ 8.03 (dd, J = 6.3, 3.5 Hz, 1H), 7.86–7.71 (m, 1H), 7.74–7.67 (m, 1H), 7.58–7.51 (m, 1H), 7.49–7.43 (m, 1H), 6.96–6.90 (m, 1H), 6.76 (t, J = 5.5 Hz, 1H), 4.61 (td, J = 4.5, 2.8 Hz, 1H)

^{13}C NMR (126 MHz, $CDCl_3$) δ 160.4, 152.4, 134.2, 129.3, 119.4, 116.4, 103.3, 99.1

HPLC (5–95% Eluent B) t_R = 6.819 min

2. NMR-Spectra

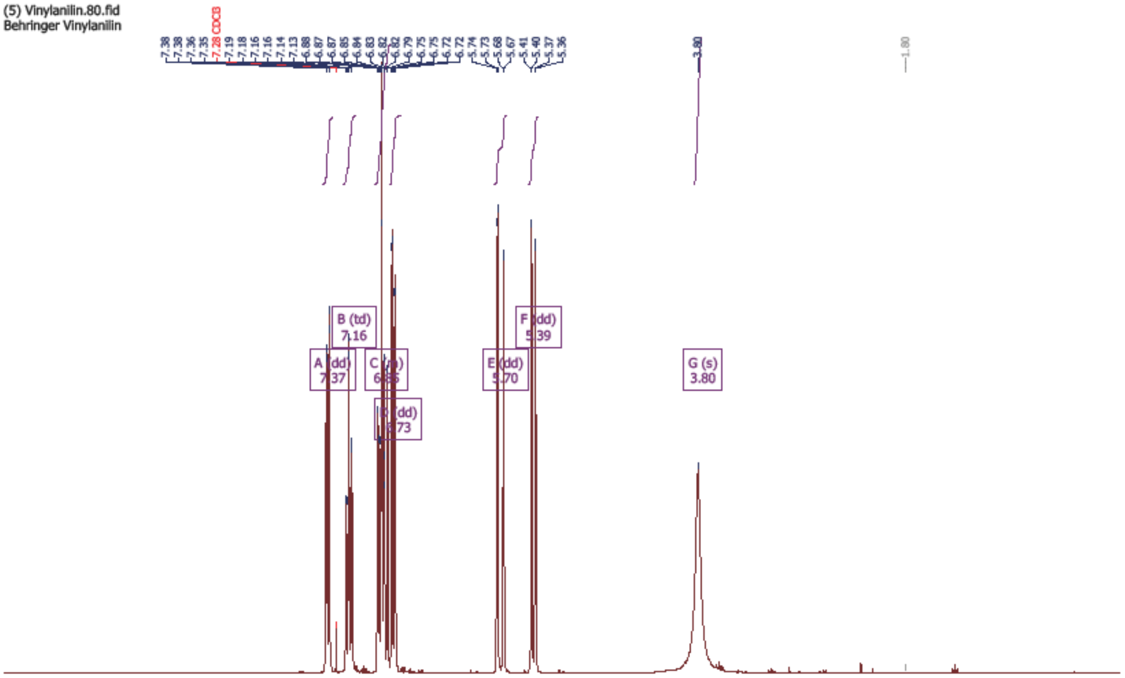

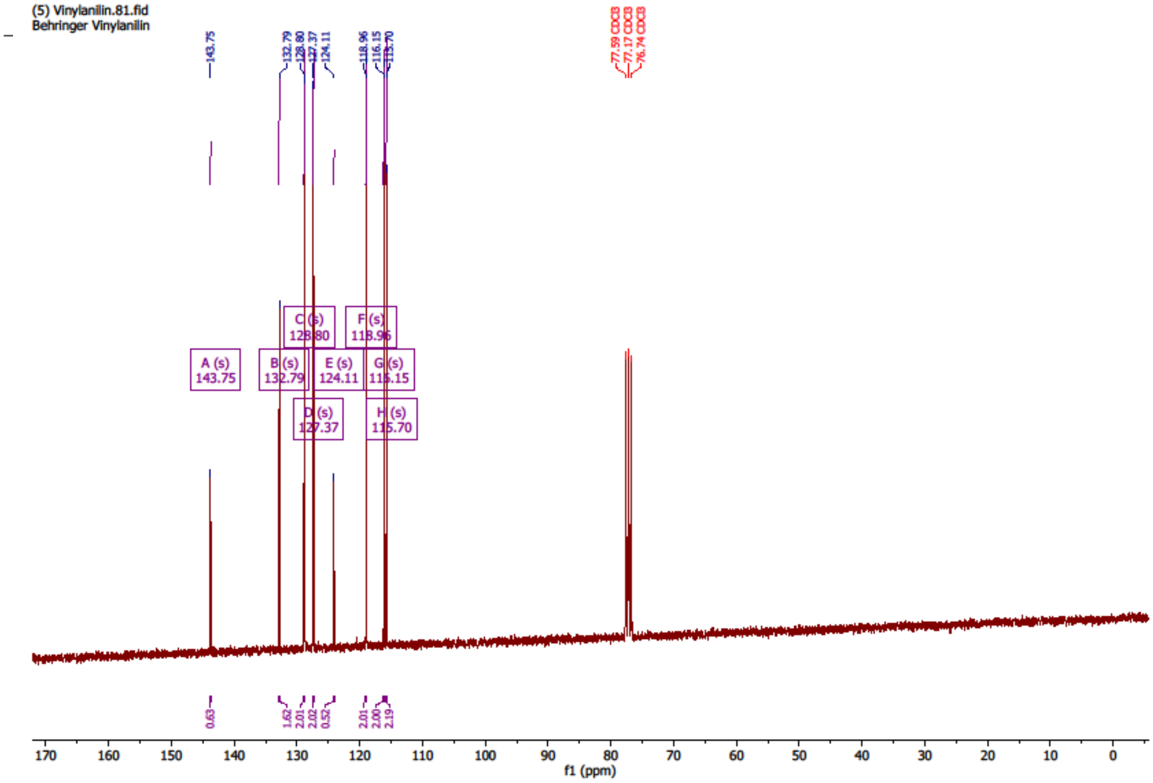

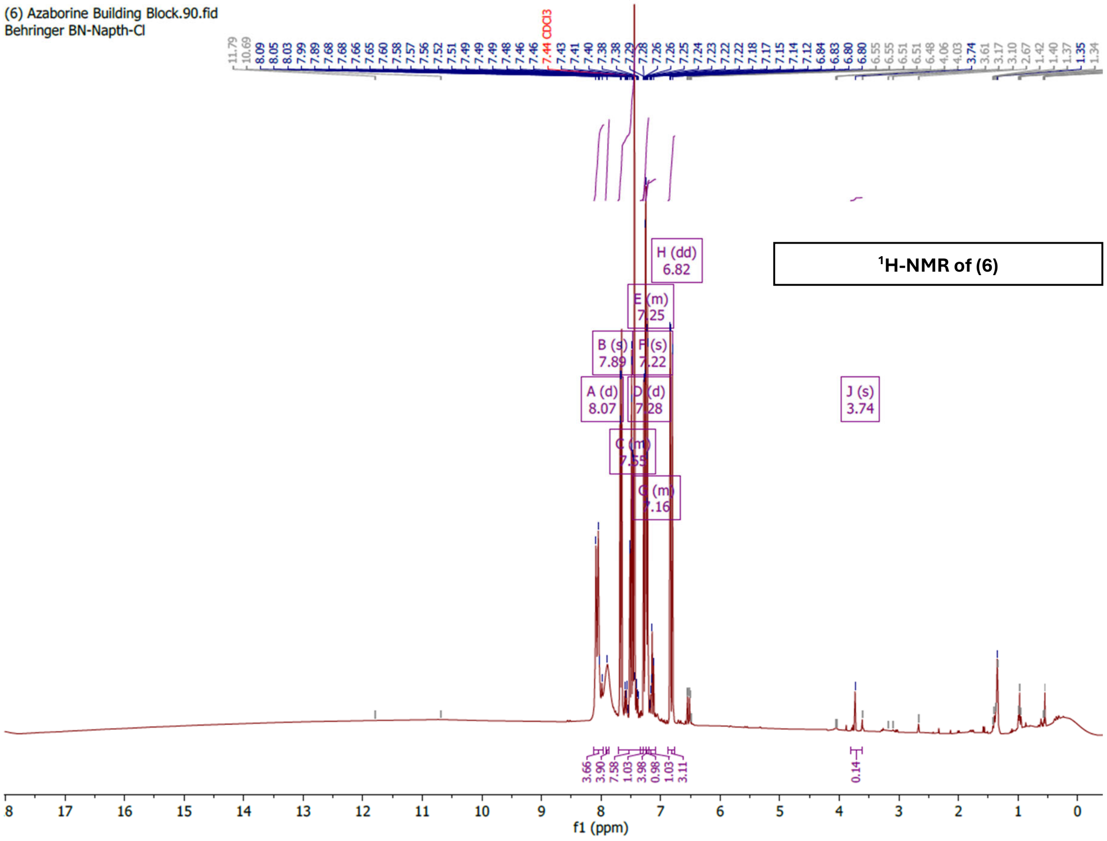
(6) Azaborine Building Block.90.fid
Behringer BN-Napth-Cl
^{1}H-NMR of (6)
H (dd) 6.82
E (m) 7.25
B (s) 7.89
F (s) 7.22
A (d) 8.07
D (d) 7.28
C (m) 7.55
G (m) 7.16
J (s) 3.74
f1 (ppm)

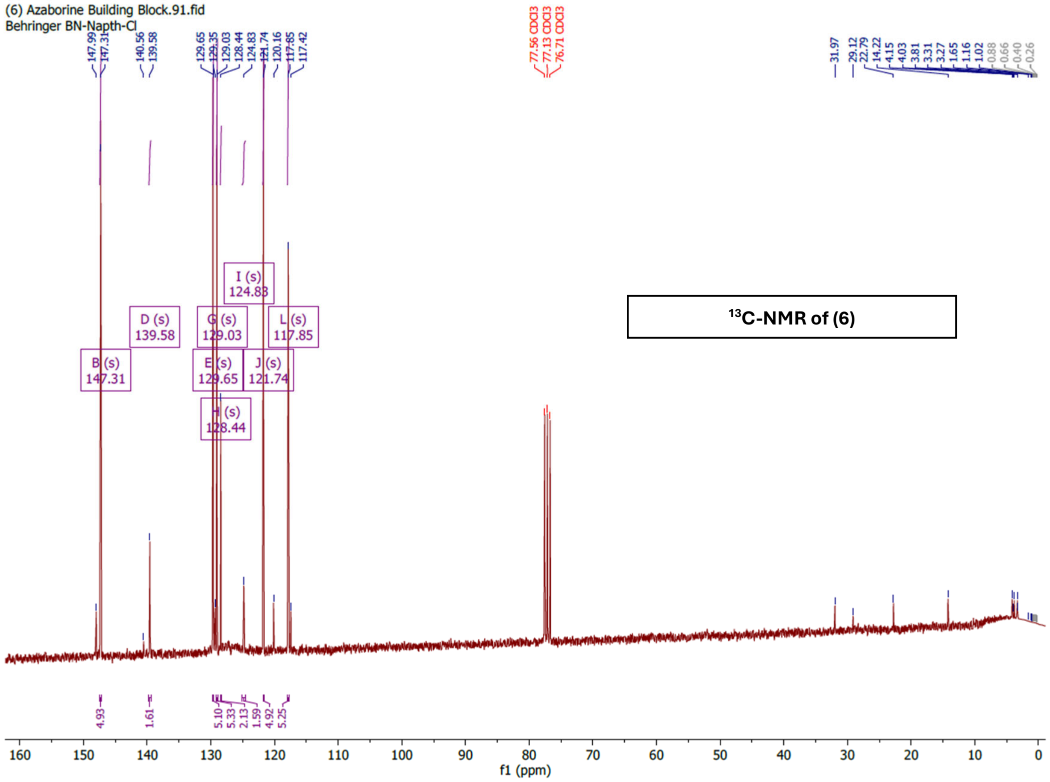
(6) Azaborine Building Block.91.fid
Behringer BN-Napth-Cl
^{13}C-NMR of (6)
I (s) 124.83
D (s) 139.58
G (s) 129.03
L (s) 117.85
B (s) 147.31
E (s) 129.65
J (s) 121.74
H (s) 128.44
f1 (ppm)

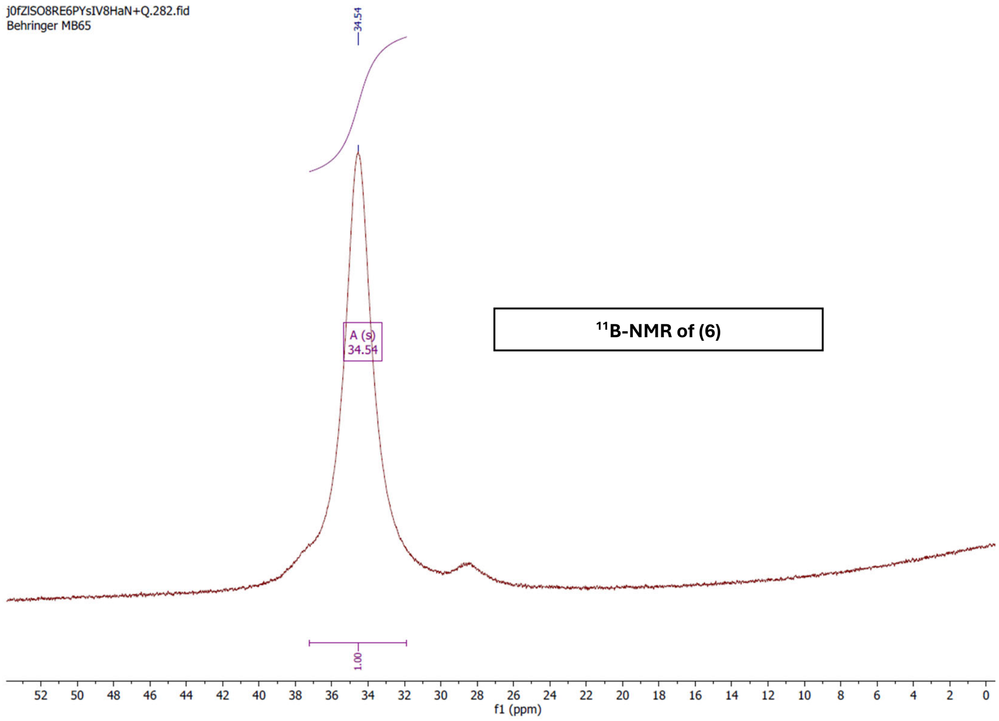

^{11}B-NMR of (6)

^{1}H-NMR of (7)

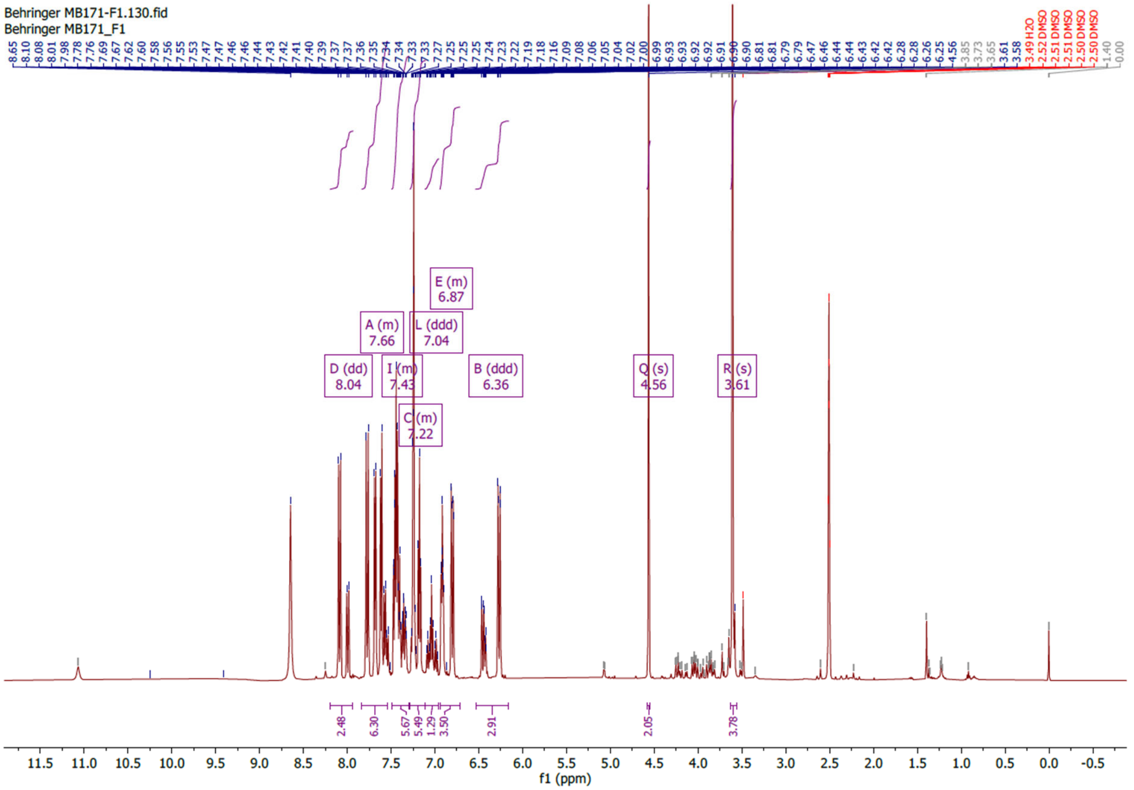
Behringer MB171-F1.130.fid
Behringer MB171_F1
D (dd) 8.04
A (m) 7.66
I (m) 7.43
L (ddd) 7.04
E (m) 6.87
C (m) 7.22
B (ddd) 6.36
Q (s) 4.56
R (s) 3.61
f1 (ppm)

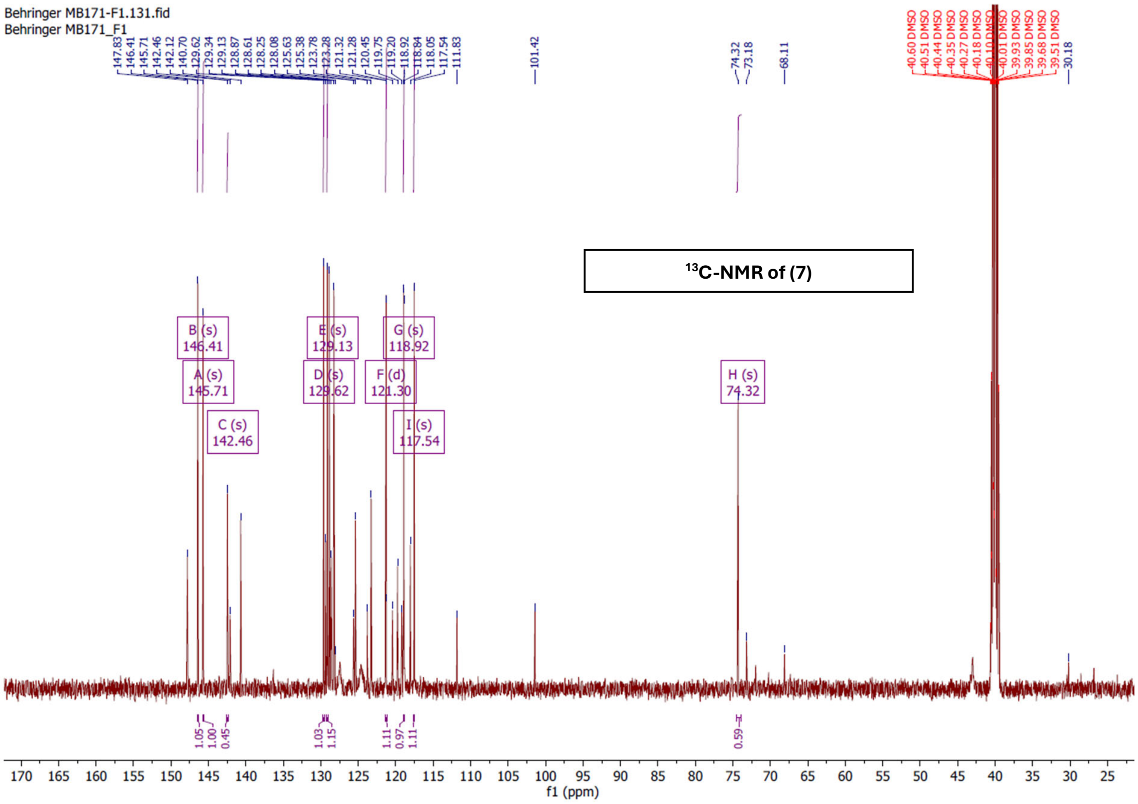
Behringer MB171-F1.131.fid
Behringer MB171_F1
¹³C-NMR of (7)
B (s) 146.41
A (s) 145.71
C (s) 142.46
E (s) 129.13
D (s) 129.62
G (s) 118.92
F (d) 121.30
I (s) 117.54
H (s) 74.32
f1 (ppm)

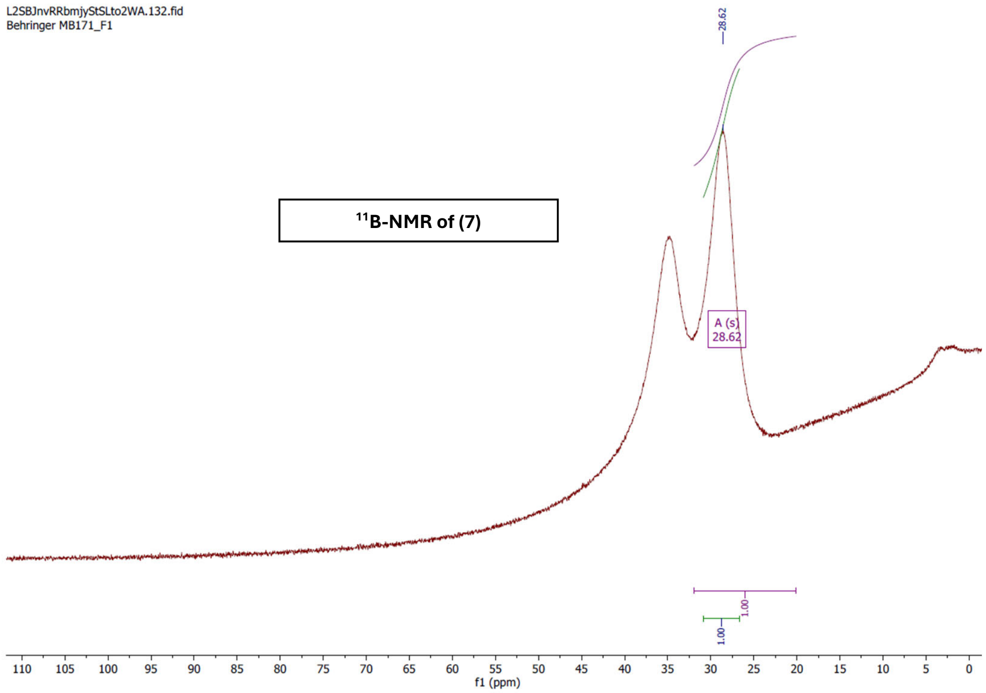
L2SBJnvRRbmjyStSLto2WA.132.fid
Behringer MB171_F1
28.62
¹¹B-NMR of (7)
A (s)
28.62
1.00
1.00
110 105 100 95 90 85 80 75 70 65 60 55 50 45 40 35 30 25 20 15 10 5 0
f1 (ppm)

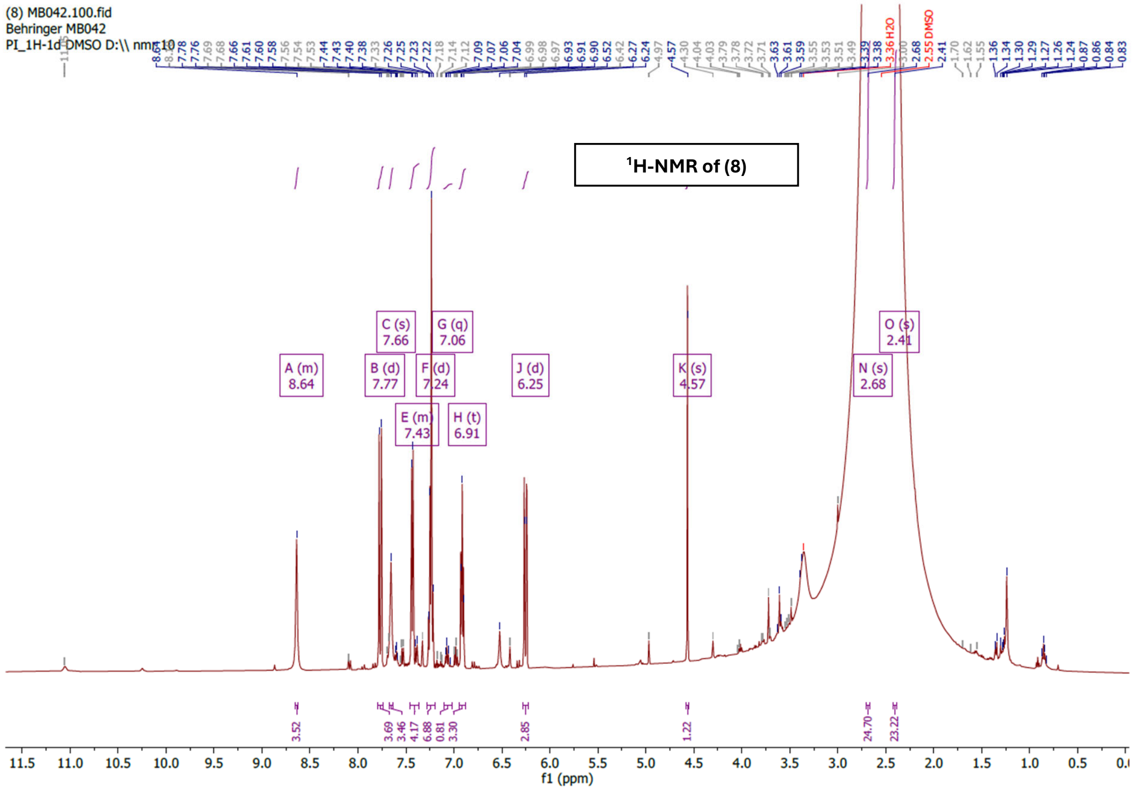
(8) MB042.100.fid
Behringer MB042
PI_1H-1d DMSO D:\\ nmr 10
1H-NMR of (8)
A (m) 8.64
B (d) 7.77
C (s) 7.66
E (m) 7.43
F (d) 7.24
G (q) 7.06
H (t) 6.91
J (d) 6.25
K (s) 4.57
N (s) 2.68
O (s) 2.41
f1 (ppm)

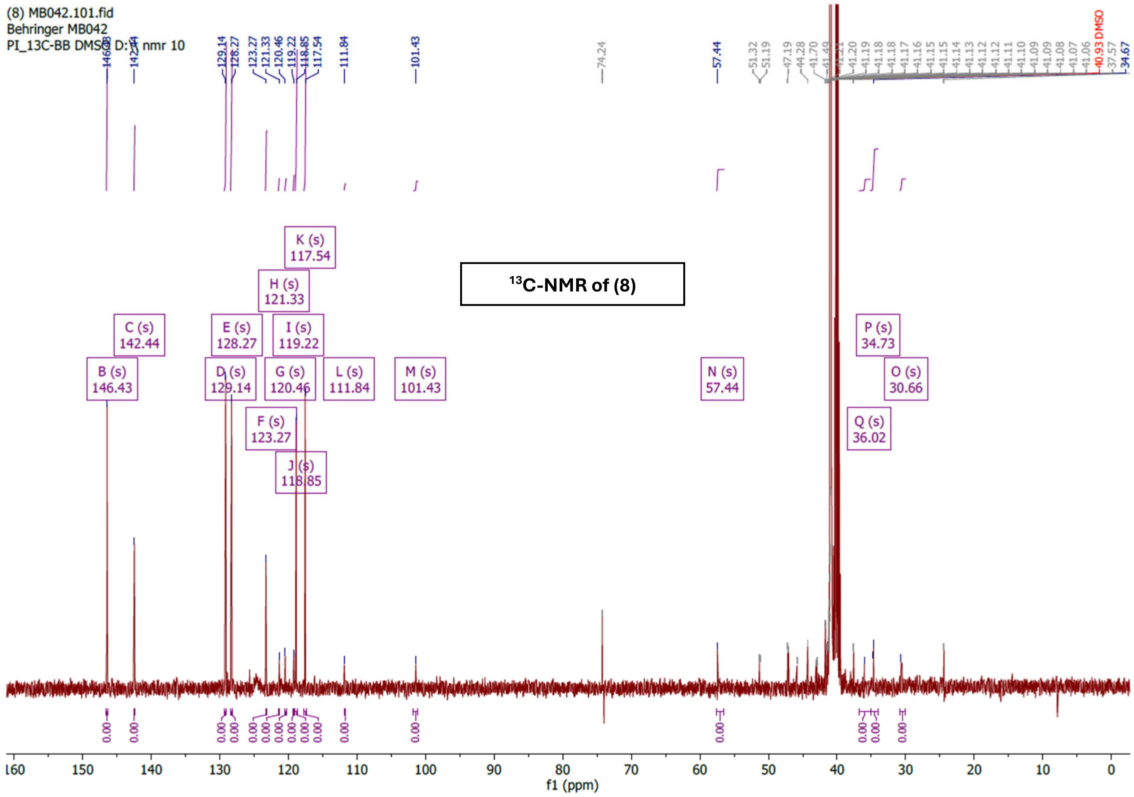
(8) MB042.101.fid
Behringer MB042
PI_13C-BB DMSO D:\\ nmr 10
13C-NMR of (8)
B (s) 146.43
C (s) 142.44
D (s) 129.14
E (s) 128.27
F (s) 123.27
H (s) 121.33
G (s) 120.46
I (s) 119.22
J (s) 118.85
K (s) 117.54
L (s) 111.84
M (s) 101.43
N (s) 57.44
Q (s) 36.02
P (s) 34.73
O (s) 30.66
f1 (ppm)

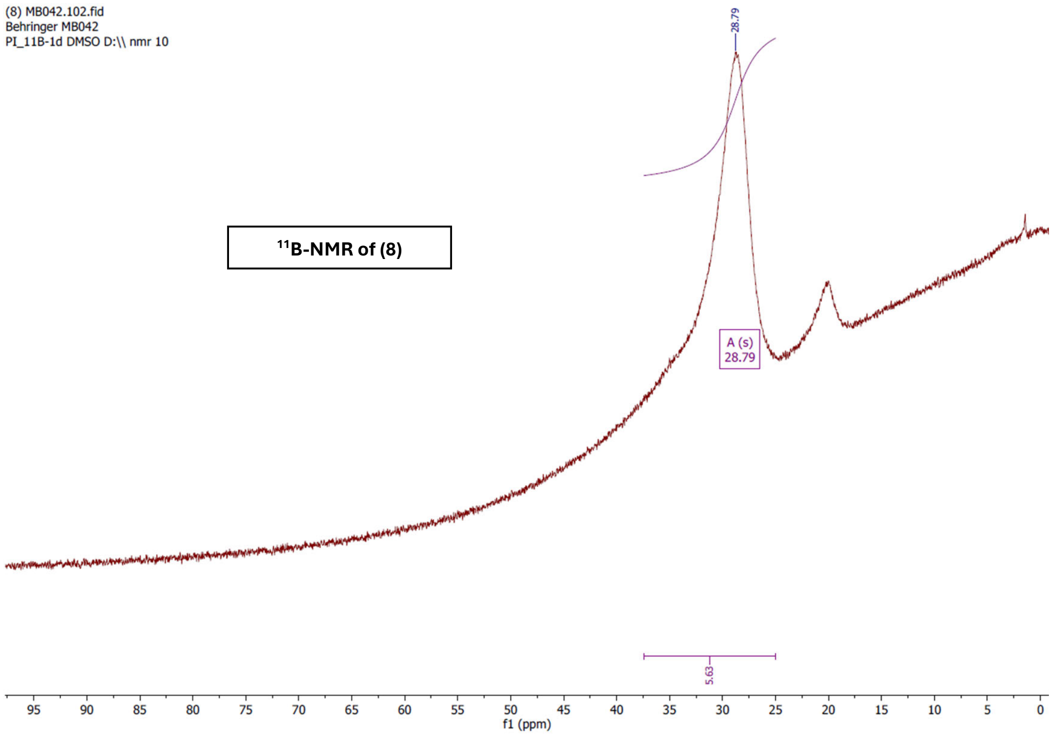

^{11}B-NMR of (8)

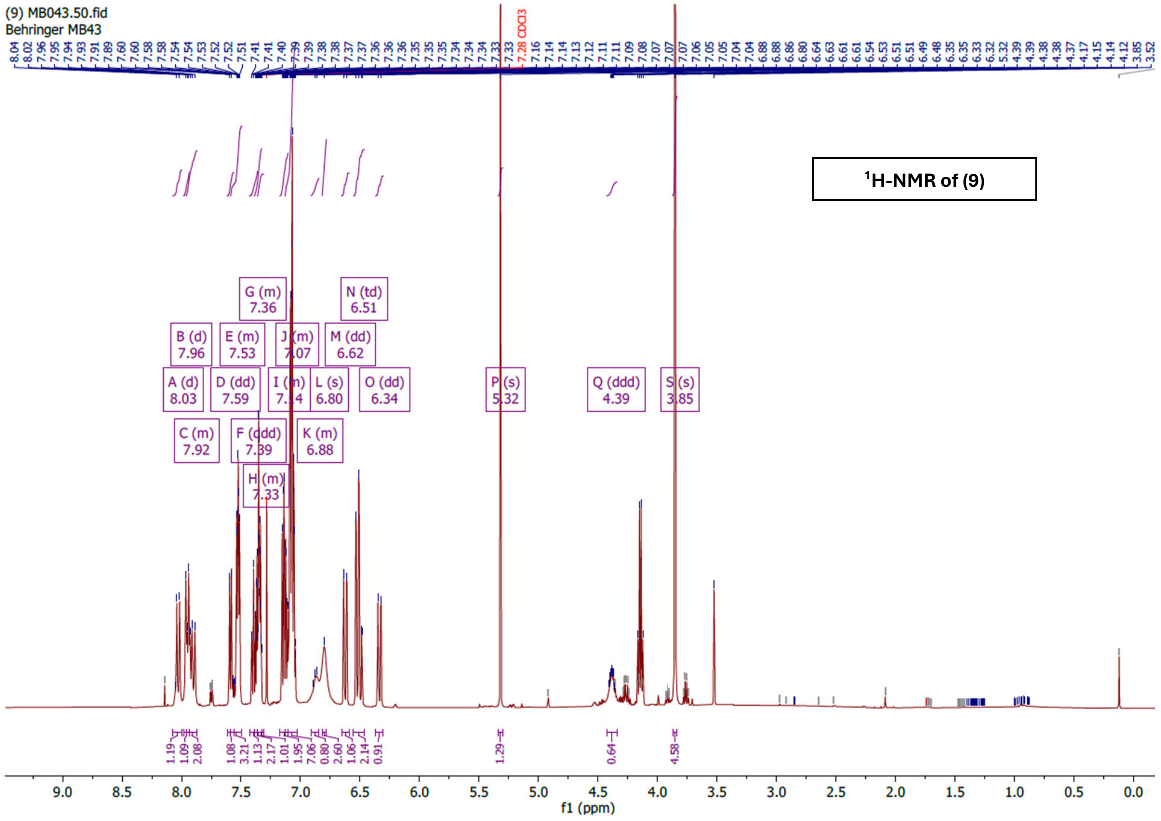

^{1}H-NMR of (9)

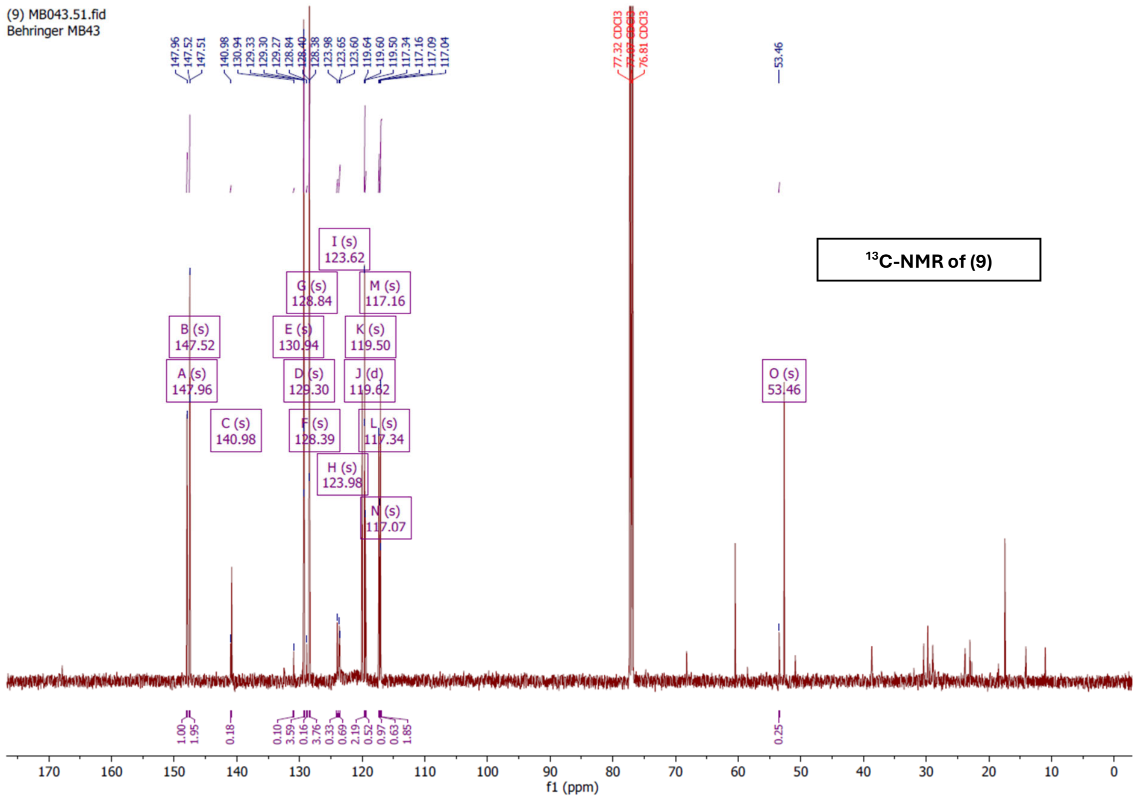

(9) MB043.51.fid
Behringer MB43
13C-NMR of (9)
f1 (ppm)

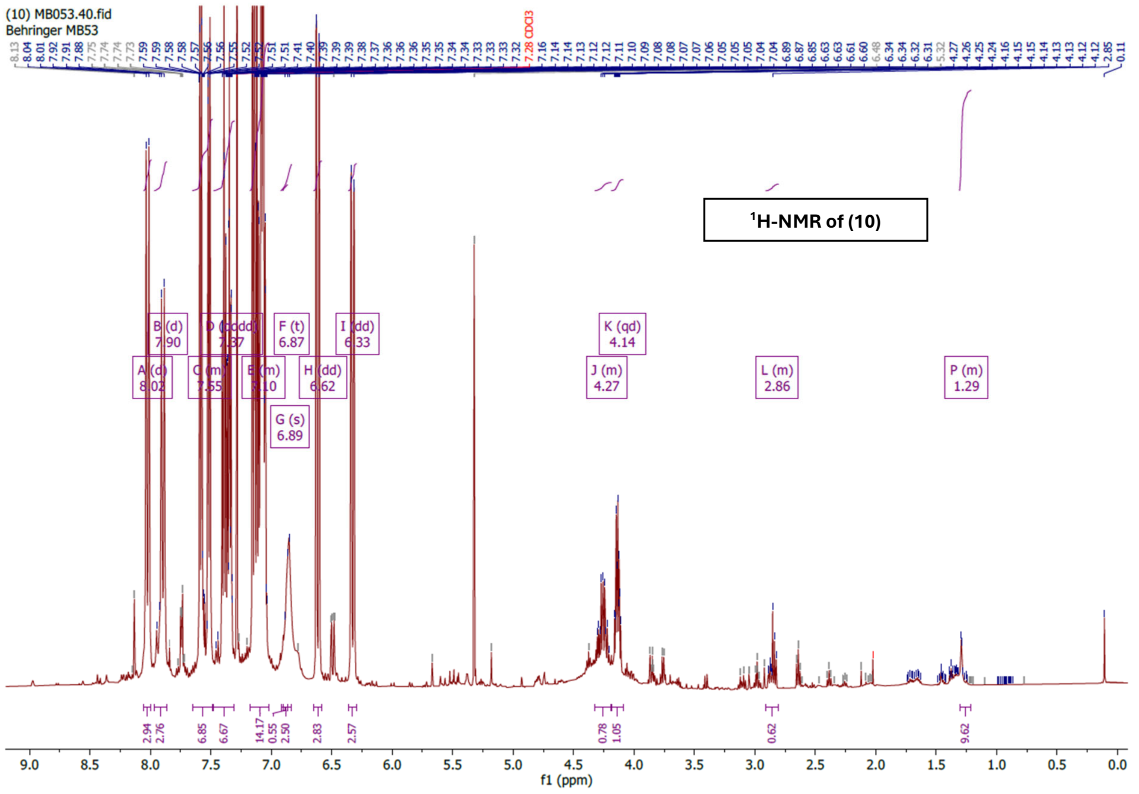

(10) MB053.40.fid
Behringer MB53
1H-NMR of (10)
f1 (ppm)

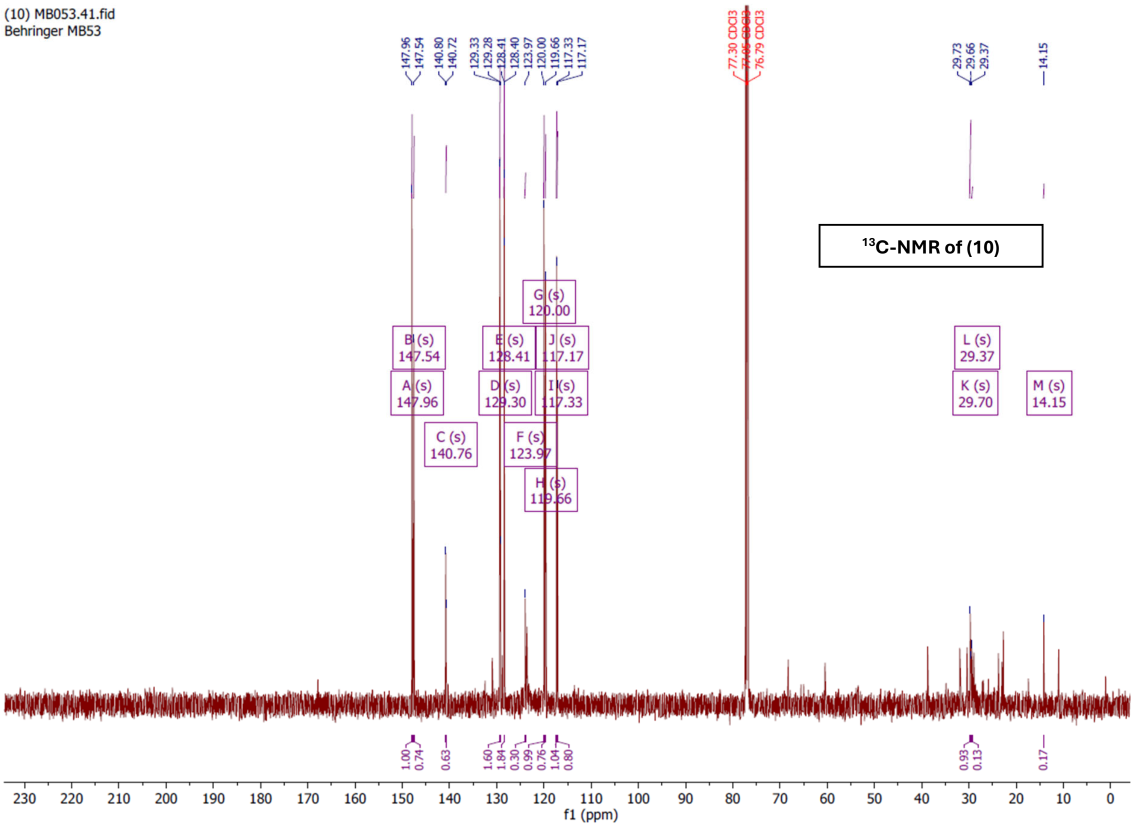
(10) MB053.41.fid
Behringer MB53
13C-NMR of (10)
A (s) 147.96
B (s) 147.54
C (s) 140.76
D (s) 129.30
E (s) 128.41
F (s) 123.97
G (s) 120.00
H (s) 119.66
I (s) 117.33
J (s) 117.17
K (s) 29.70
L (s) 29.37
M (s) 14.15
f1 (ppm)

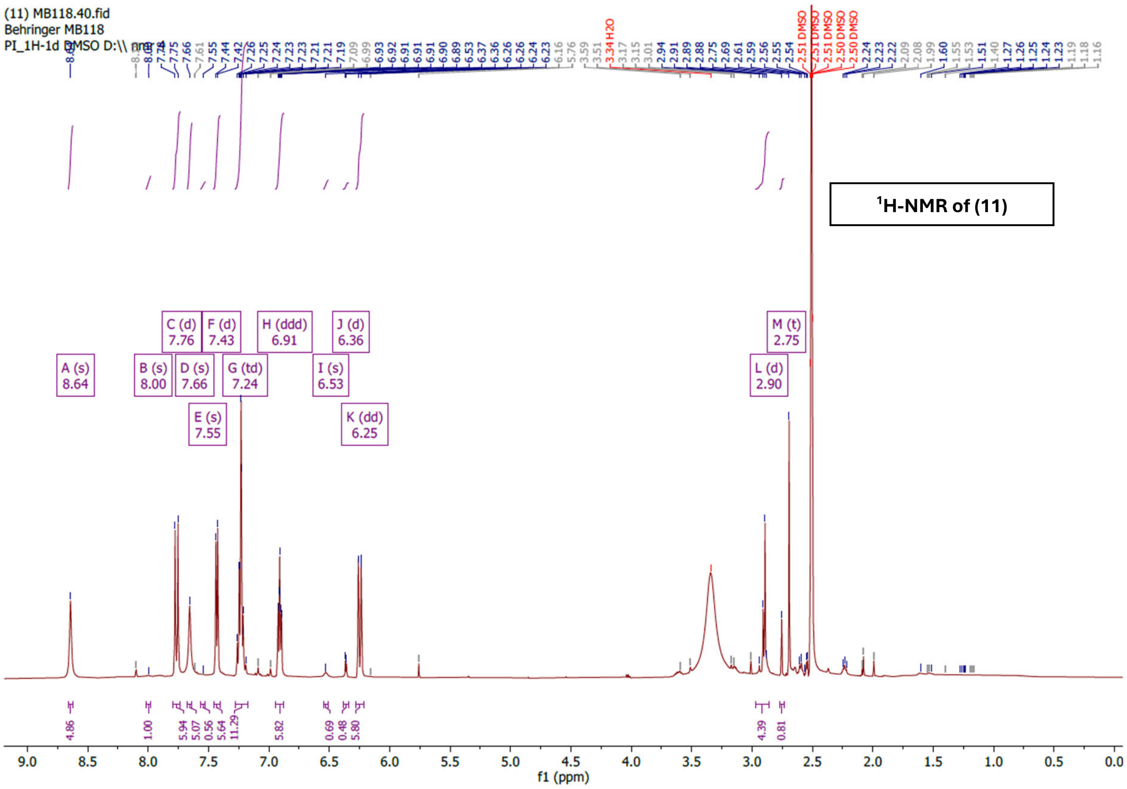
(11) MB118.40.fid
Behringer MB118
1H-NMR of (11)
A (s) 8.64
B (s) 8.00
C (d) 7.76
D (s) 7.66
E (s) 7.55
F (d) 7.43
G (td) 7.24
H (ddd) 6.91
I (s) 6.53
J (d) 6.36
K (dd) 6.25
L (d) 2.90
M (t) 2.75
f1 (ppm)

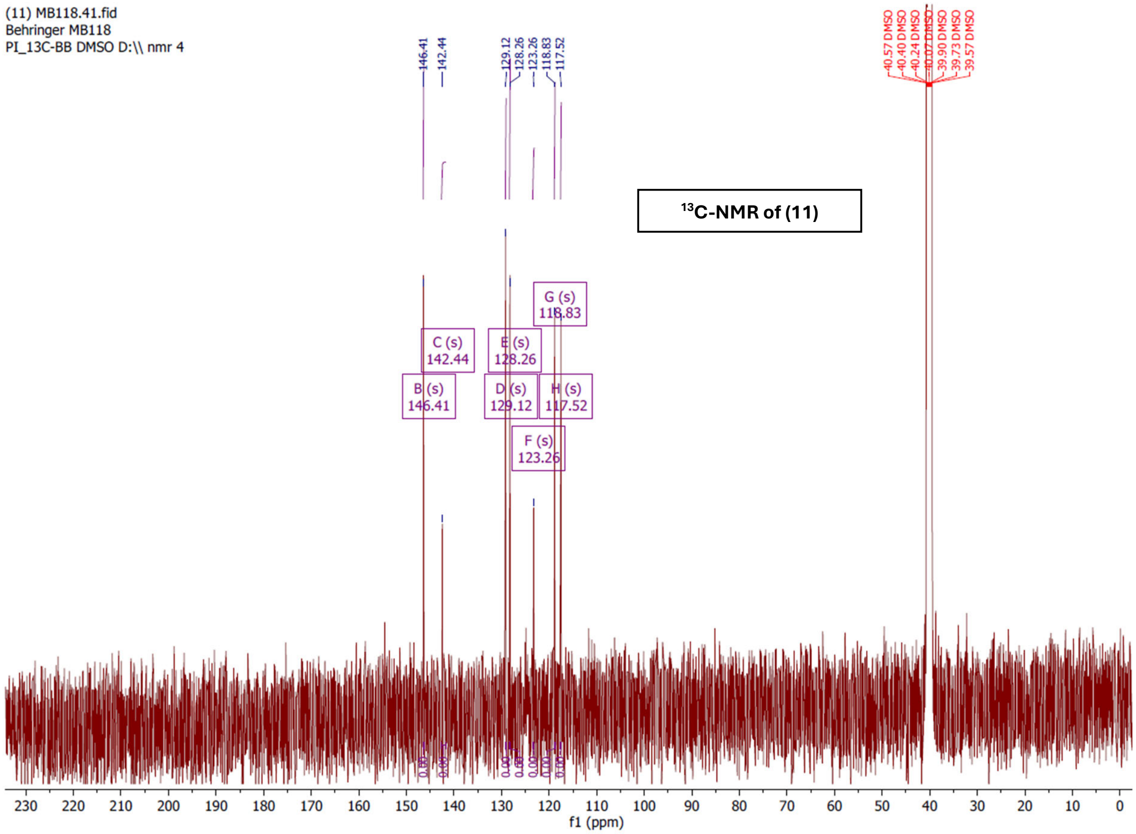

(11) MB118.41.fid
Behringer MB118
PI_13C-BB DMSO D:\\ nmr 4
146.41
142.44
129.12
128.26
123.26
118.83
117.52
40.57 DMSO
40.40 DMSO
40.24 DMSO
40.07 DMSO
39.90 DMSO
39.73 DMSO
39.57 DMSO
13C-NMR of (11)
G (s) 118.83
C (s) 142.44
E (s) 128.26
B (s) 146.41
D (s) 129.12
H (s) 117.52
F (s) 123.26
f1 (ppm)

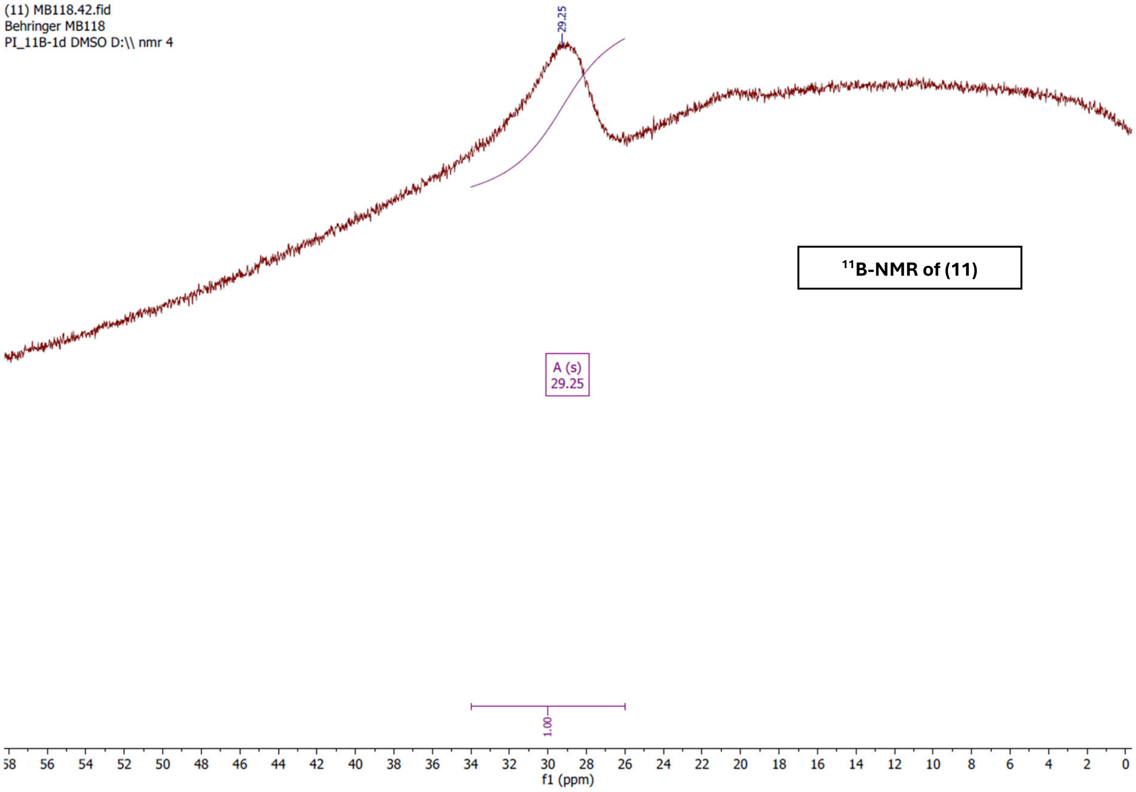

(11) MB118.42.fid
Behringer MB118
PI_11B-1d DMSO D:\\ nmr 4
29.25
11B-NMR of (11)
A (s) 29.25
1.00
f1 (ppm)

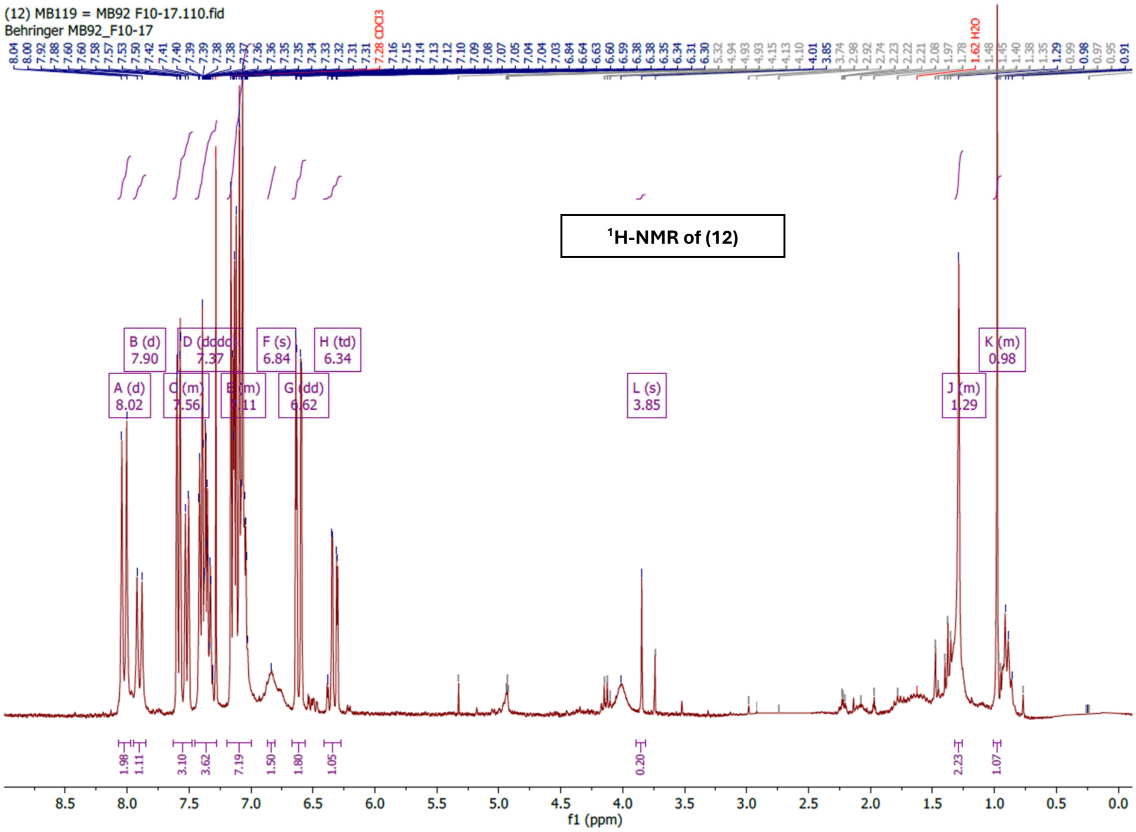
(12) MB119 = MB92 F10-17.110.fid
Behringer MB92_F10-17
¹H-NMR of (12)
A (d) 8.02
B (d) 7.90
C (m) 7.56
D (dddd) 7.37
E (m) 7.11
F (s) 6.84
G (dd) 6.62
H (td) 6.34
L (s) 3.85
J (m) 1.29
K (m) 0.98
f1 (ppm)

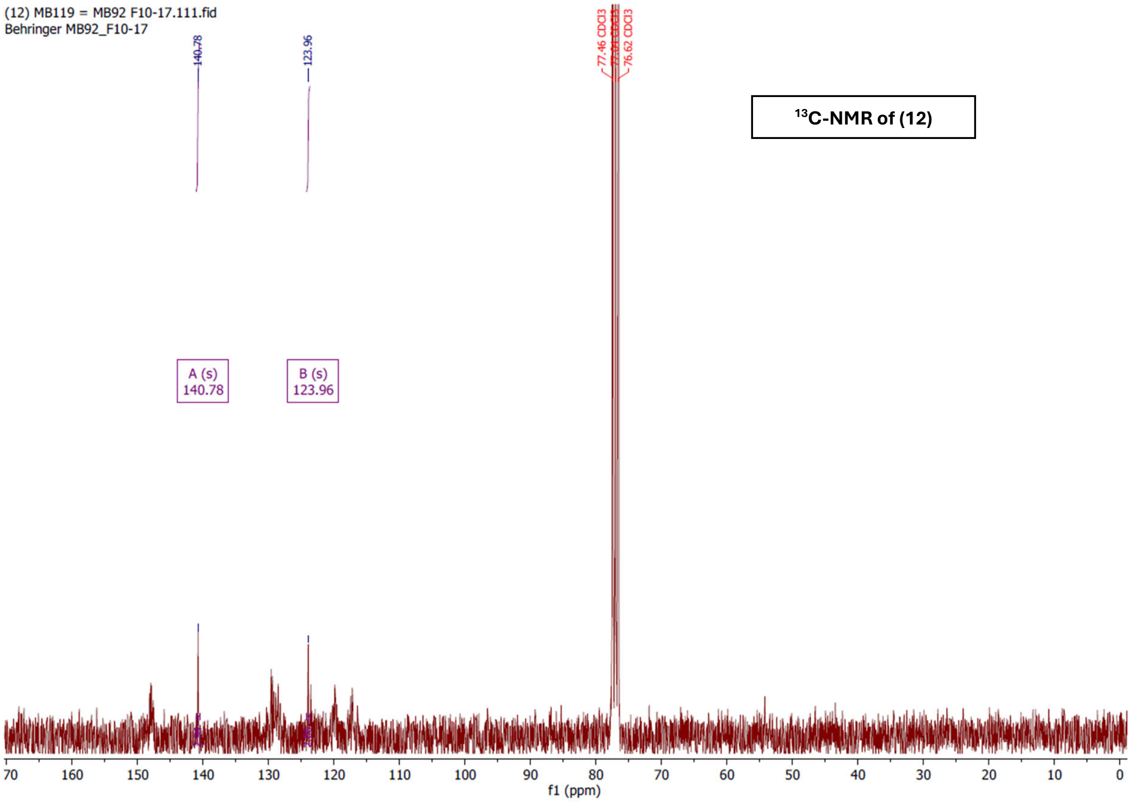
(12) MB119 = MB92 F10-17.111.fid
Behringer MB92_F10-17
¹³C-NMR of (12)
A (s) 140.78
B (s) 123.96
f1 (ppm)

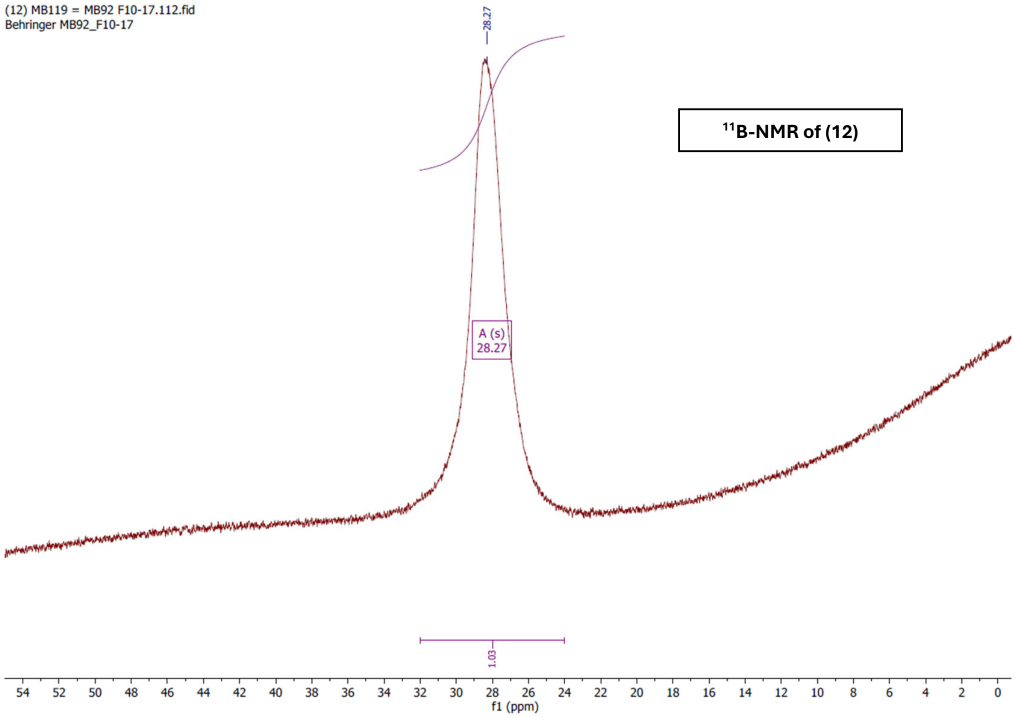
(12) MB119 = MB92 F10-17.112.fid
Behringer MB92_F10-17
28.27
¹¹B-NMR of (12)
A (s)
28.27
1.03
f1 (ppm)

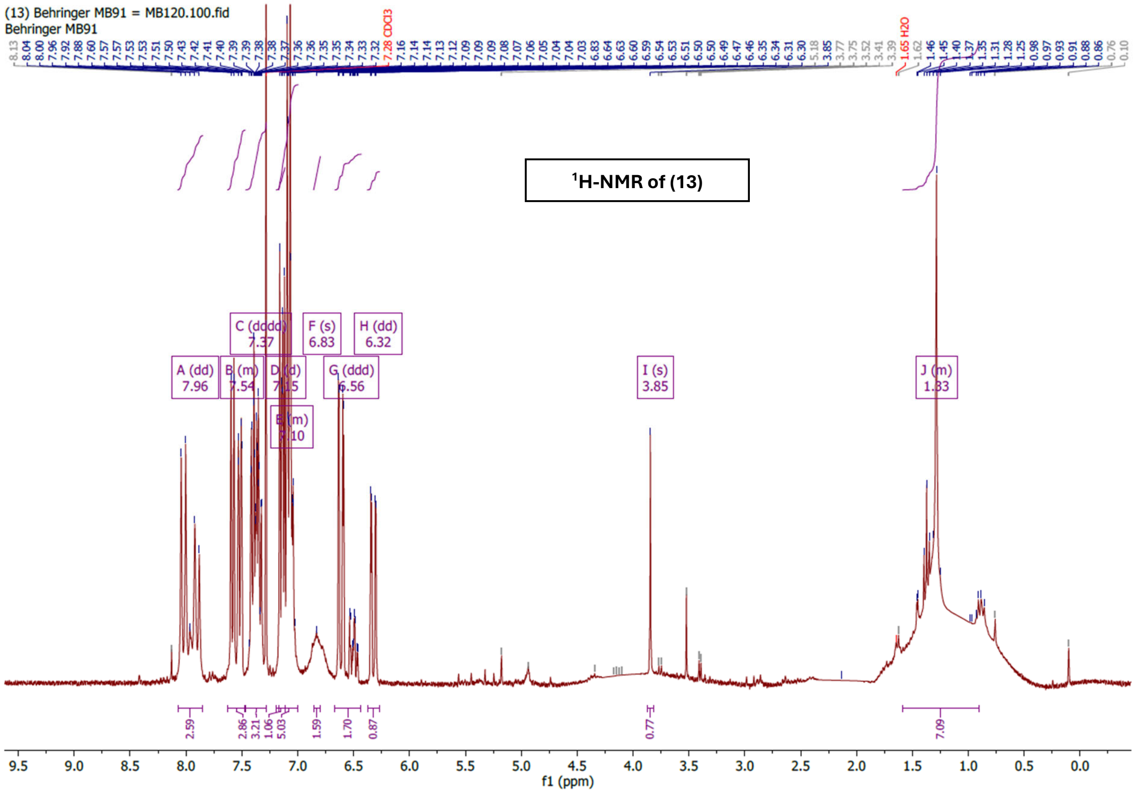
(13) Behringer MB91 = MB120.100.fid
Behringer MB91
¹H-NMR of (13)
A (dd)
7.96
B (m)
7.54
C (dddd)
7.37
D (d)
7.15
E (m)
7.10
F (s)
6.83
G (ddd)
6.56
H (dd)
6.32
I (s)
3.85
J (m)
1.33
7.28 CDCl3
1.65 H2O
f1 (ppm)

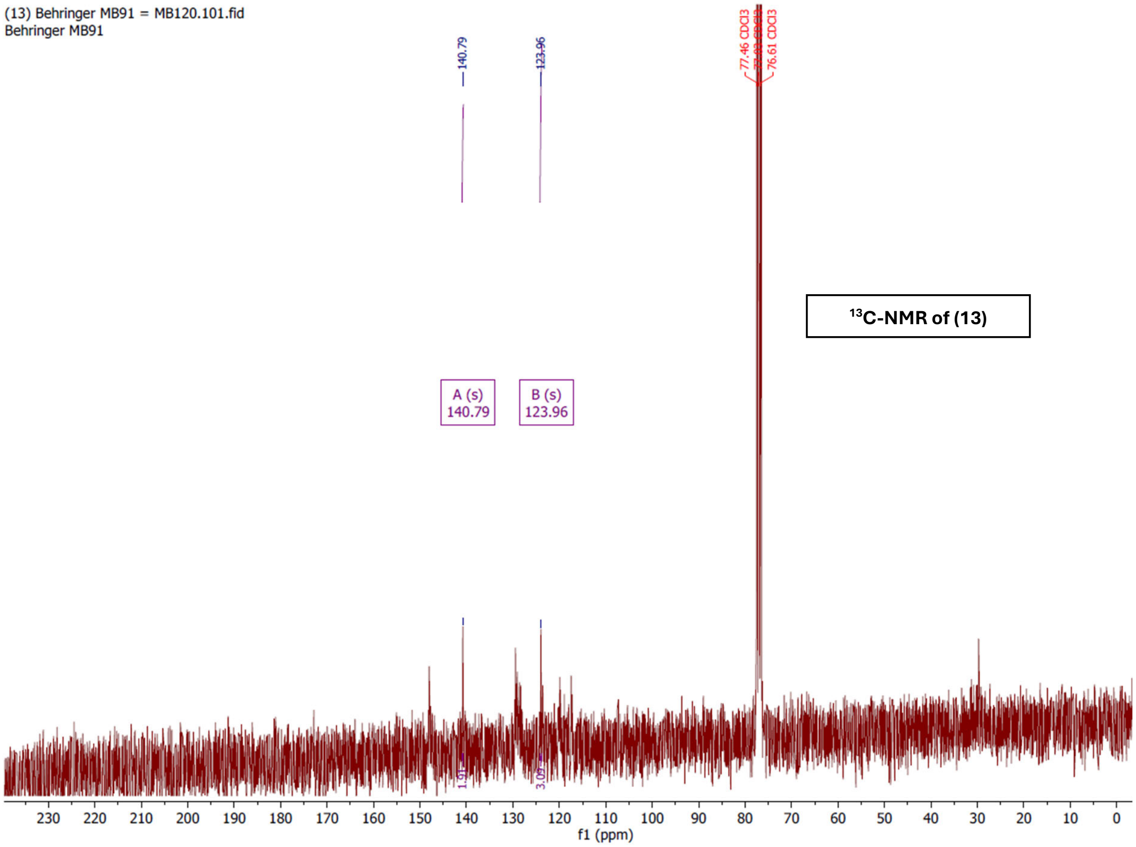
(13) Behringer MB91 = MB120.101.fid
Behringer MB91
140.79
123.96
77.46 CDCl3
76.61 CDCl3
^{13}C-NMR of (13)
A (s)
140.79
B (s)
123.96
1.01
3.09
230 220 210 200 190 180 170 160 150 140 130 120 110 100 90 80 70 60 50 40 30 20 10 0
f1 (ppm)

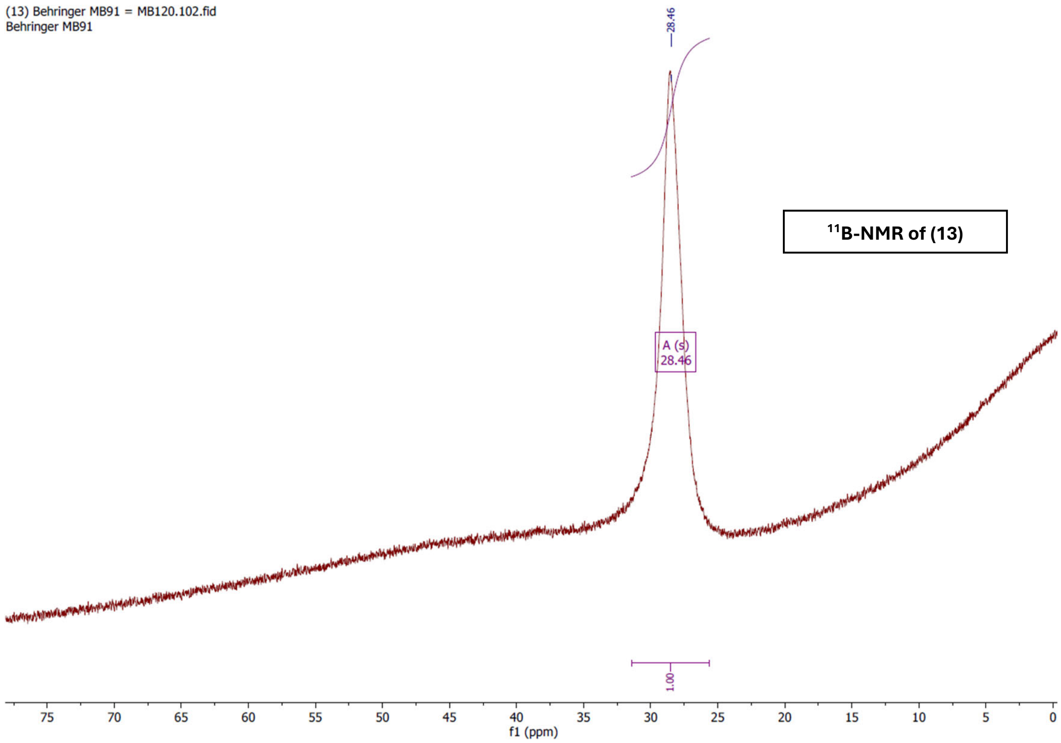
(13) Behringer MB91 = MB120.102.fid
Behringer MB91
28.46
^{11}B-NMR of (13)
A (s)
28.46
1.00
75 70 65 60 55 50 45 40 35 30 25 20 15 10 5 0
f1 (ppm)

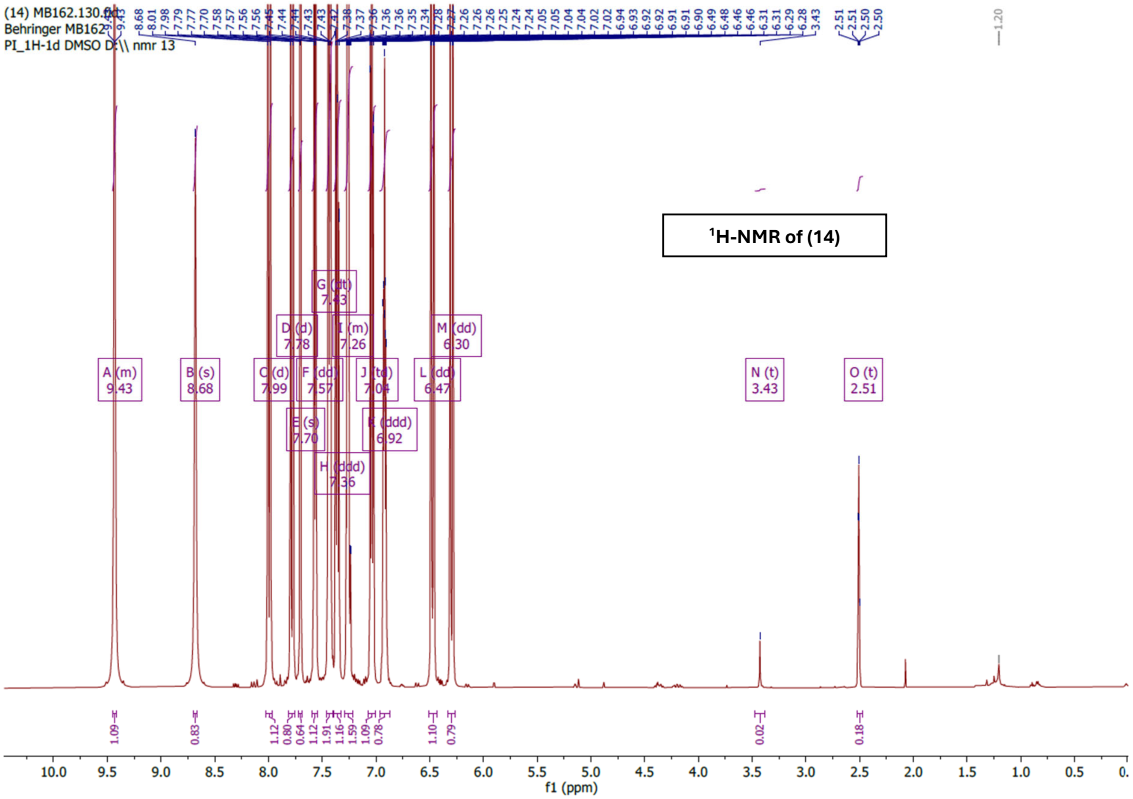

^{1}H-NMR of (14)

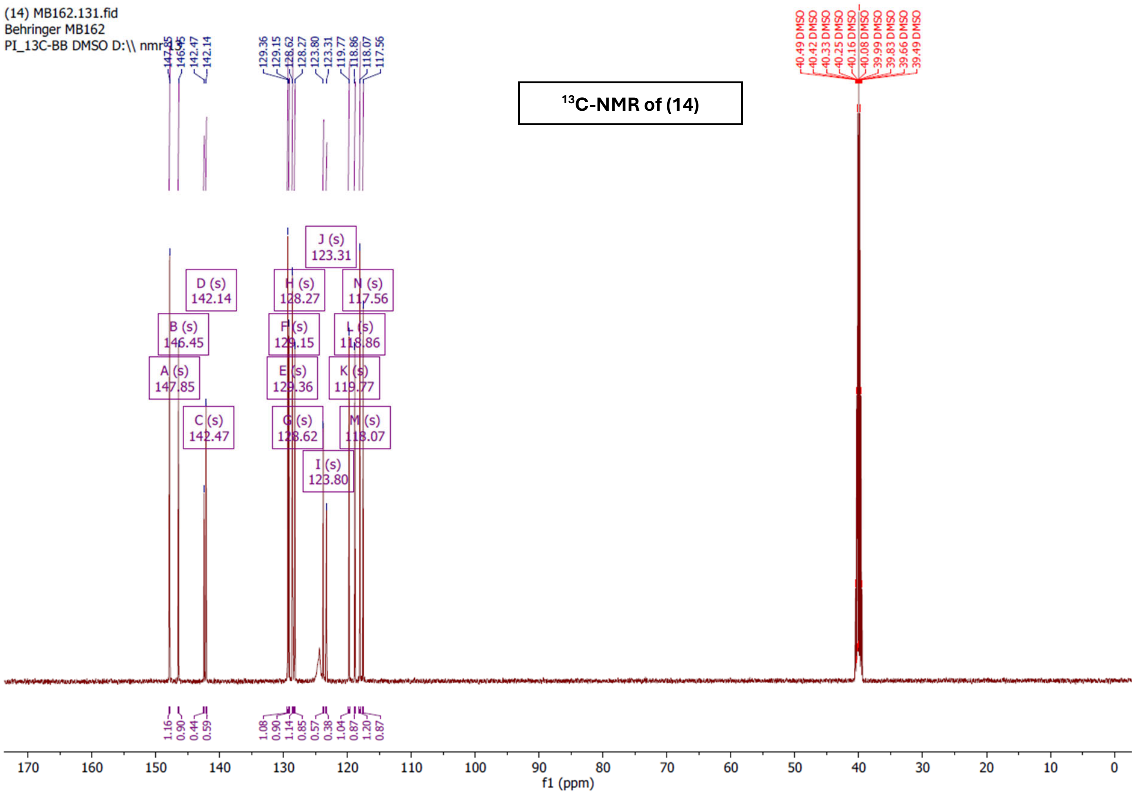

^{13}C-NMR of (14)

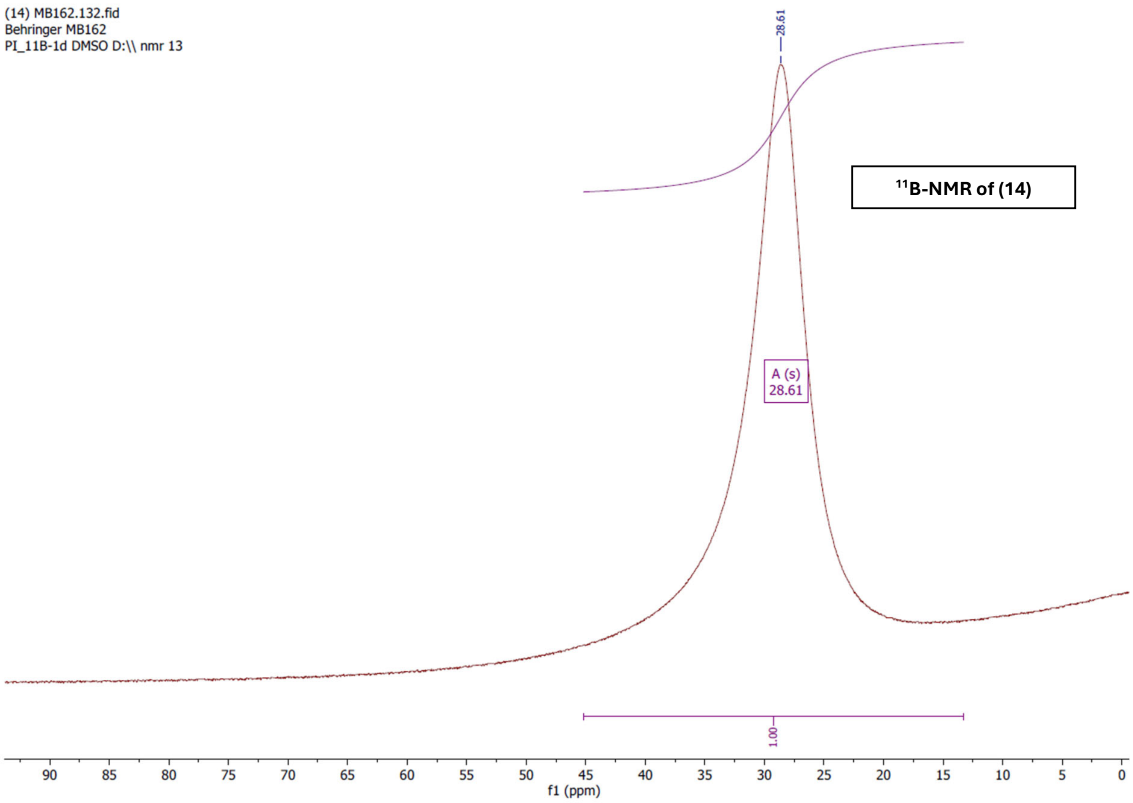
(14) MB162.132.fid
Behringer MB162
PI_11B-1d DMSO D:\\ nmr 13
28.61
A (s)
28.61
1.00
90
85
80
75
70
65
60
55
50
45
40
35
30
25
20
15
10
5
0
f1 (ppm)

^{11}B-NMR of (14)

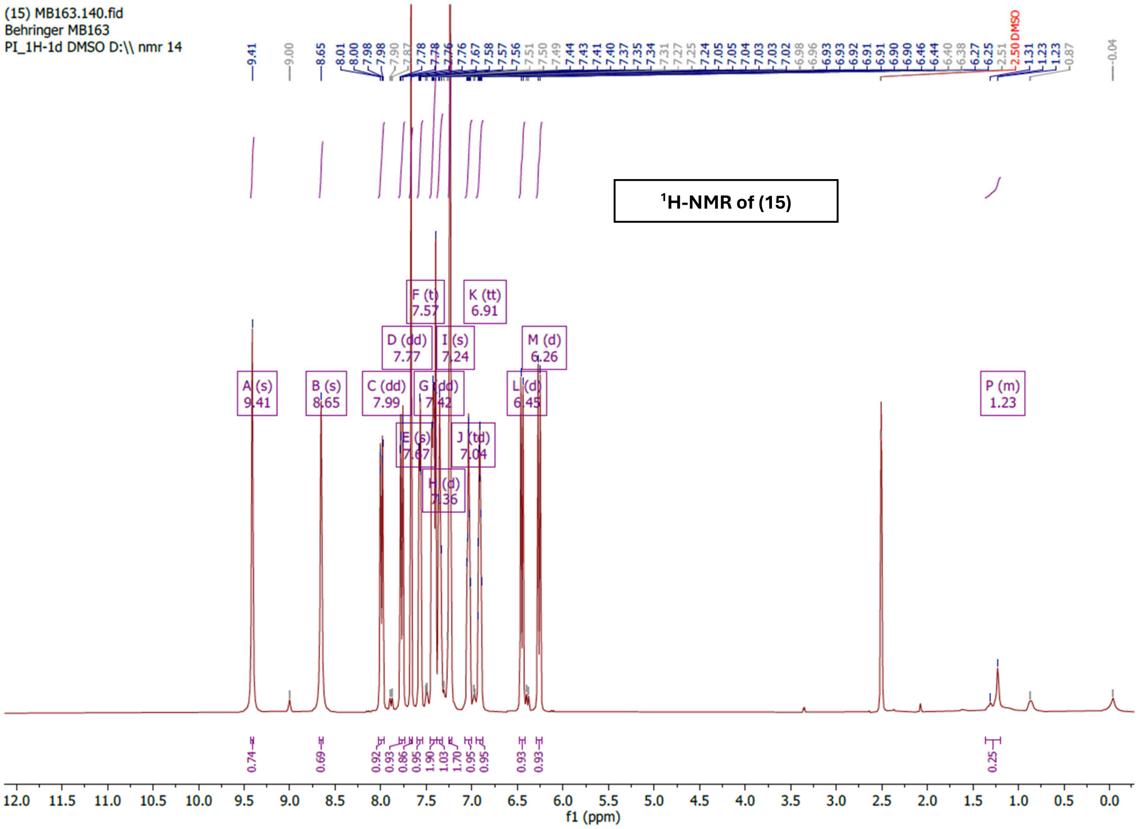
(15) MB163.140.fid
Behringer MB163
PI_1H-1d DMSO D:\\ nmr 14
1H-NMR of (15)
f1 (ppm)

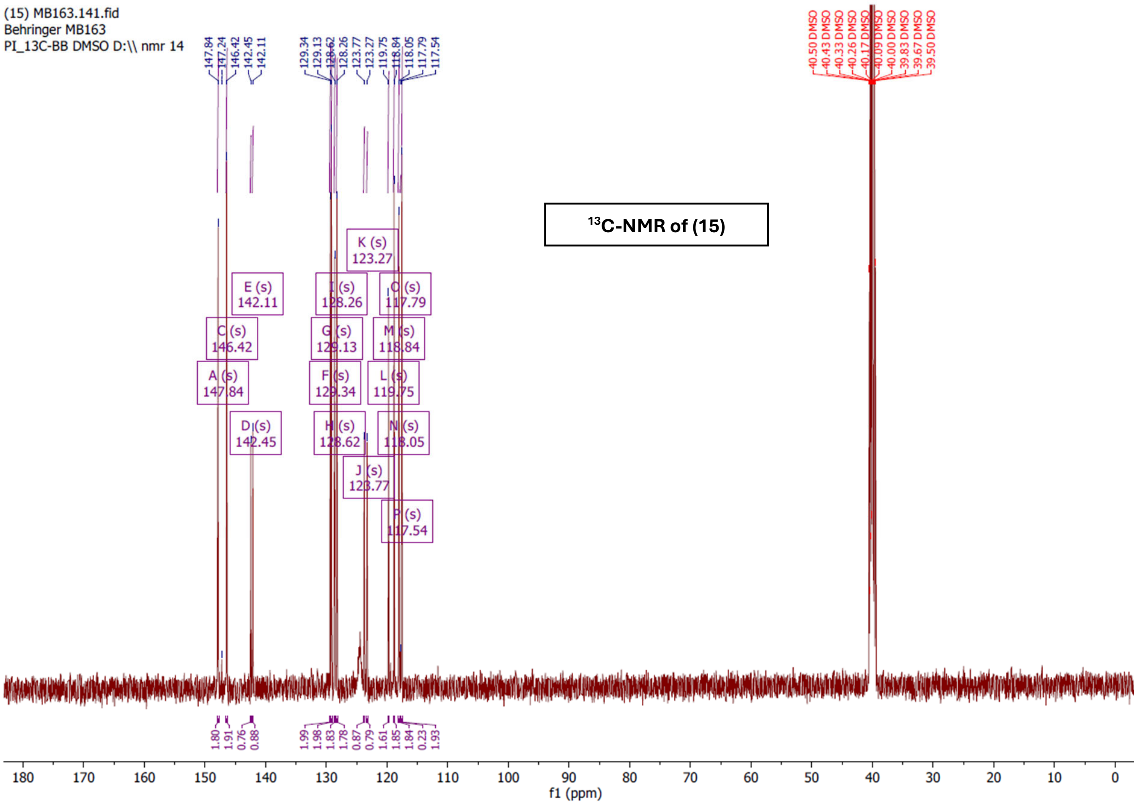
(15) MB163.141.fid
Behringer MB163
PI_13C-BB DMSO D:\\ nmr 14
13C-NMR of (15)
f1 (ppm)

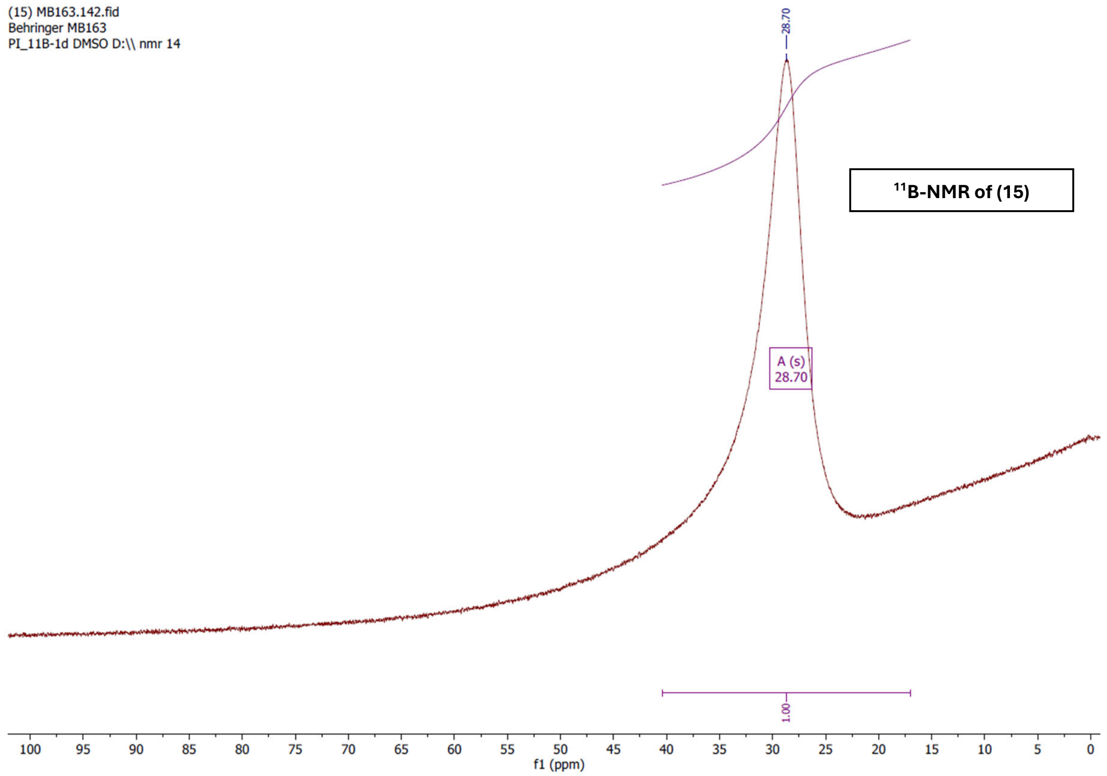
(15) MB163.142.fid
Behringer MB163
PI_11B-1d DMSO D:\\ nmr 14
28.70
^{11}B-NMR of (15)
A (s)
28.70
1.00
100 95 90 85 80 75 70 65 60 55 50 45 40 35 30 25 20 15 10 5 0
f1 (ppm)

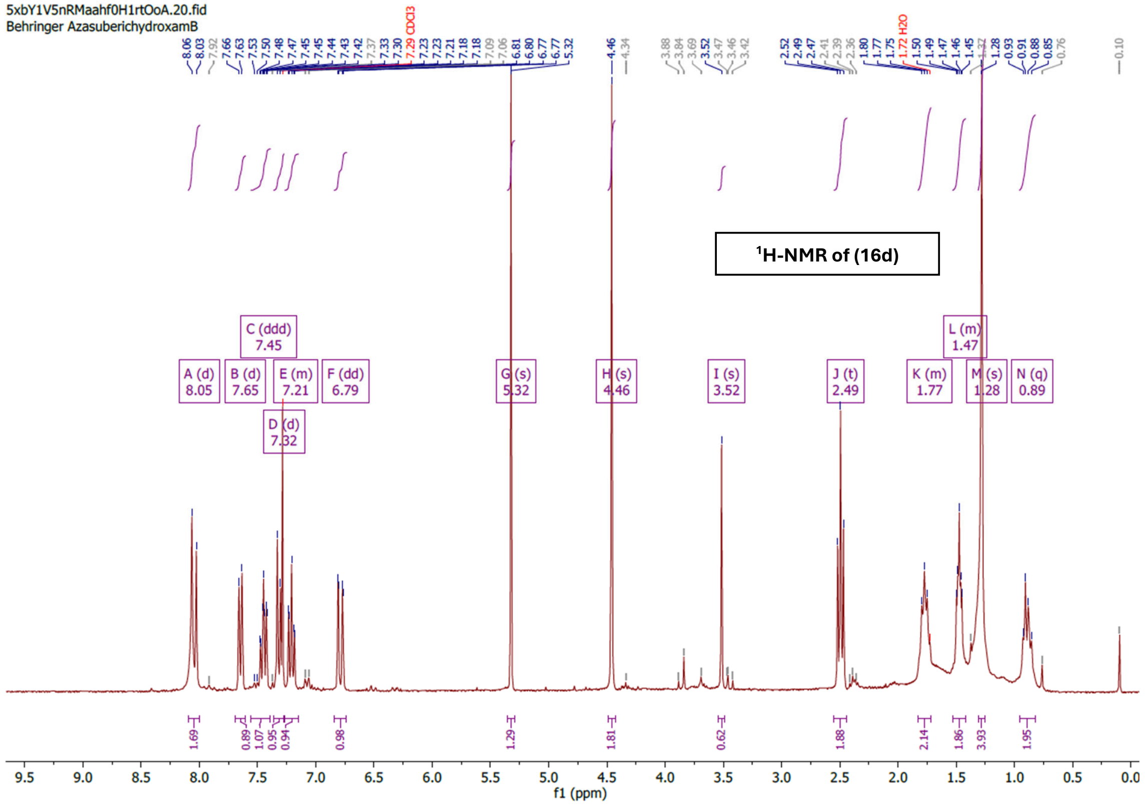
5xbY1V5nRMaahf0H1rtOoA.20.fid
Behringer AzasuberichydroxamB
7.29 CDCl3
1.72 H2O
^{1}H-NMR of (16d)
A (d)
8.05
B (d)
7.65
C (ddd)
7.45
D (d)
7.32
E (m)
7.21
F (dd)
6.79
G (s)
5.32
H (s)
4.46
I (s)
3.52
J (t)
2.49
K (m)
1.77
L (m)
1.47
M (s)
1.28
N (q)
0.89
1.69
0.89
1.07
0.95
0.94
0.98
1.29
1.81
0.62
1.88
2.14
1.86
3.93
1.95
9.5 9.0 8.5 8.0 7.5 7.0 6.5 6.0 5.5 5.0 4.5 4.0 3.5 3.0 2.5 2.0 1.5 1.0 0.5 0.0
f1 (ppm)

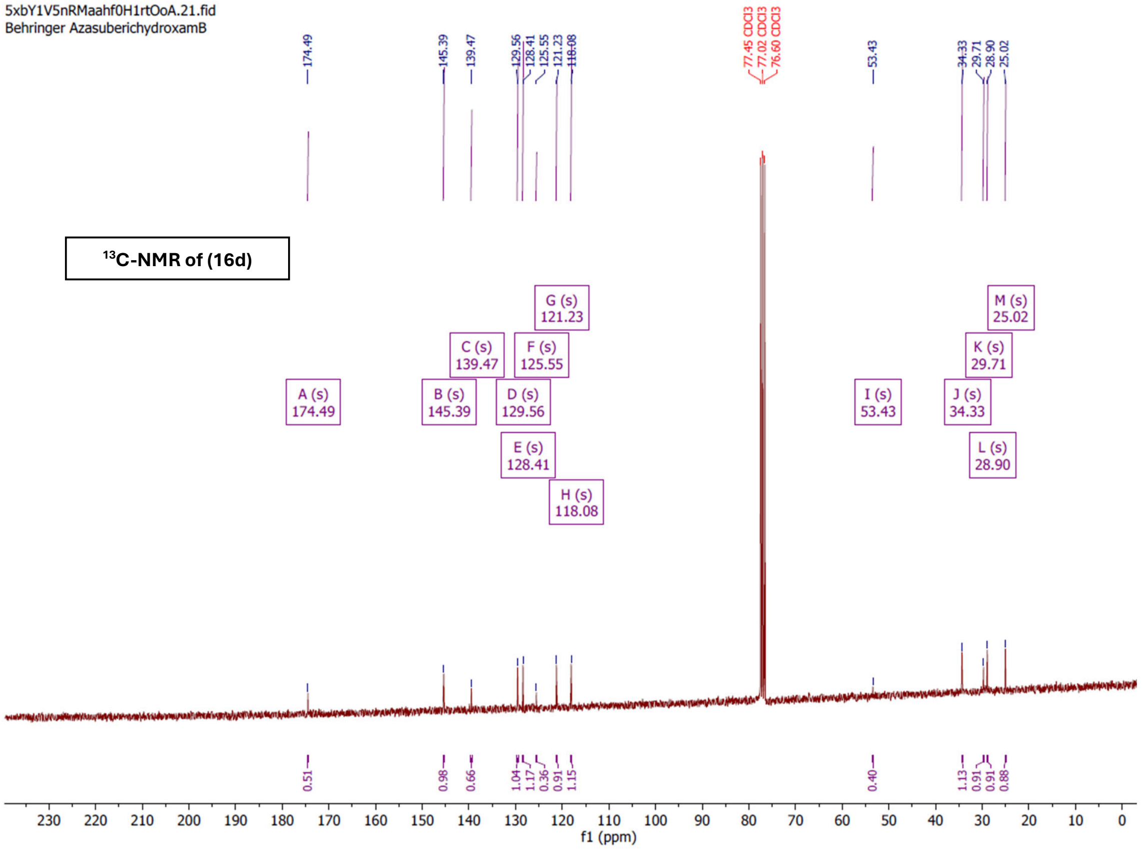
5xbY1V5nRMaahf0H1rtOoA.21.fid
Behringer AzasuberichydroxamB
¹³C-NMR of (16d)
A (s) 174.49
B (s) 145.39
C (s) 139.47
D (s) 129.56
E (s) 128.41
F (s) 125.55
G (s) 121.23
H (s) 118.08
I (s) 53.43
J (s) 34.33
K (s) 29.71
L (s) 28.90
M (s) 25.02
77.45 CDCl3
77.02 CDCl3
76.60 CDCl3
f1 (ppm)

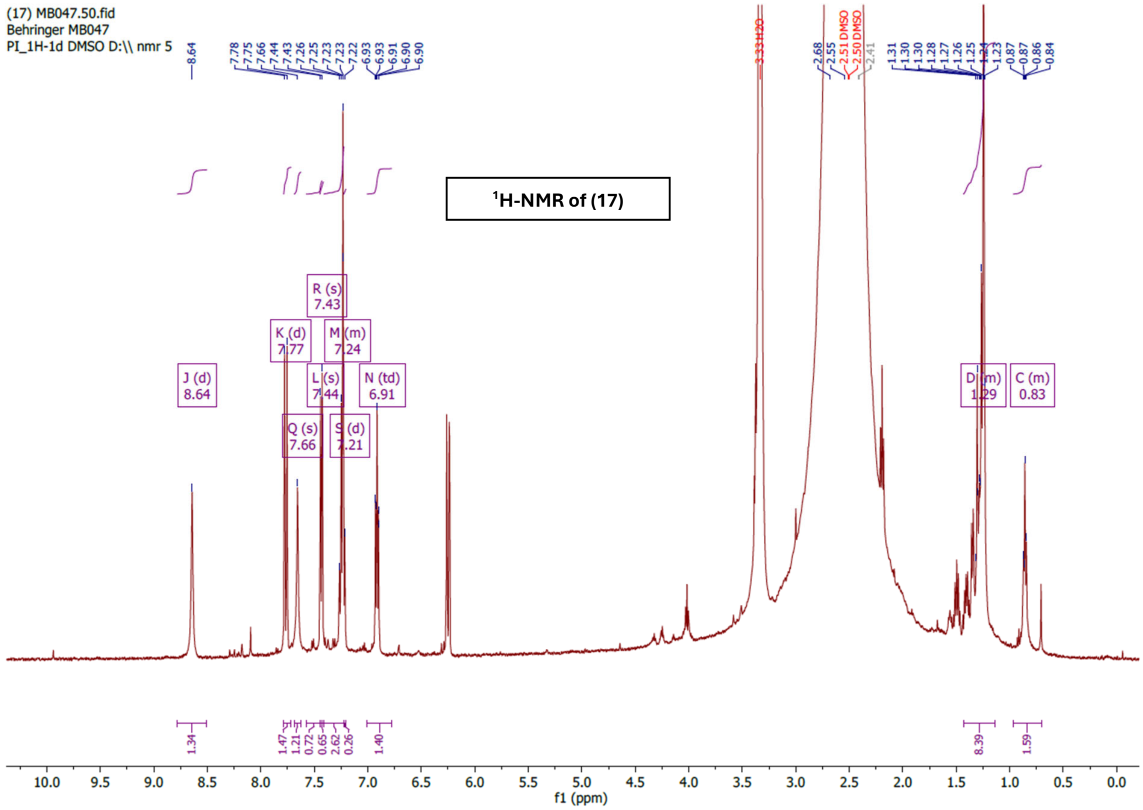
(17) MB047.50.fid
Behringer MB047
PI_1H-1d DMSO D:\\ nmr 5
¹H-NMR of (17)
J (d) 8.64
K (d) 7.77
L (s) 7.44
Q (s) 7.66
R (s) 7.43
M (m) 7.24
S (d) 7.21
N (td) 6.91
D (m) 1.29
C (m) 0.83
3.33 H2O
2.51 DMSO
2.50 DMSO
f1 (ppm)

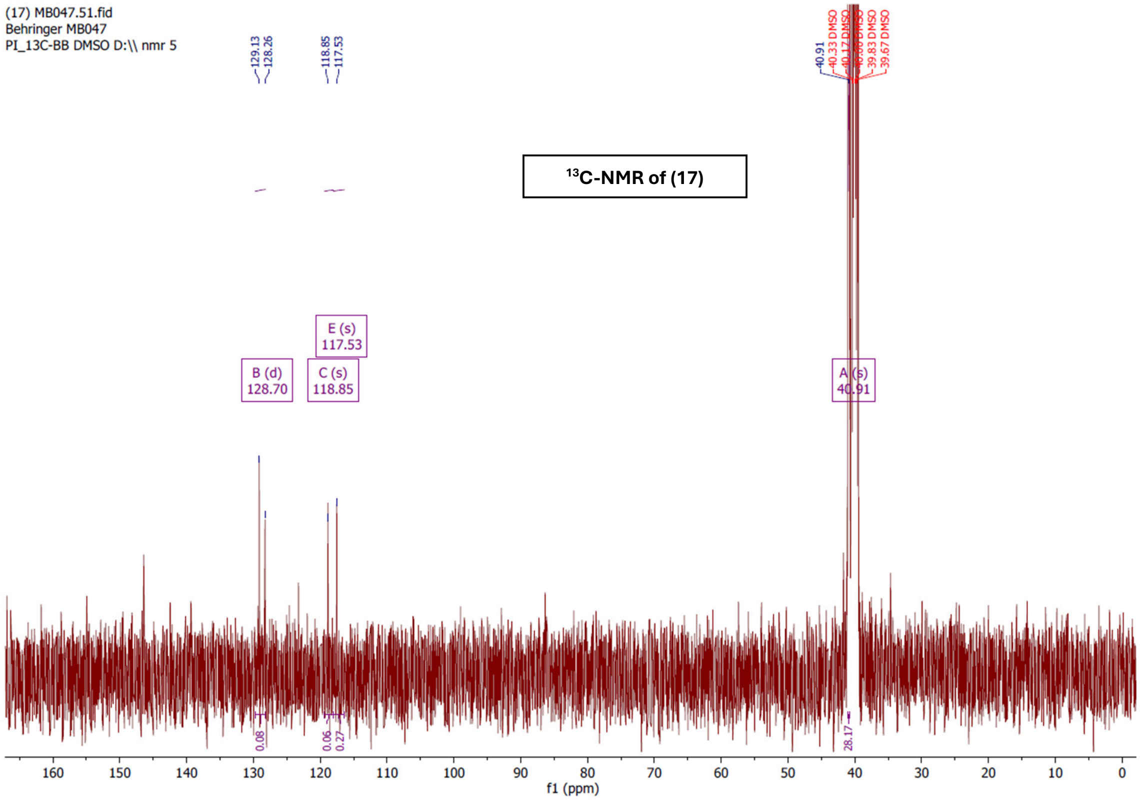

^{13}C-NMR of (17)

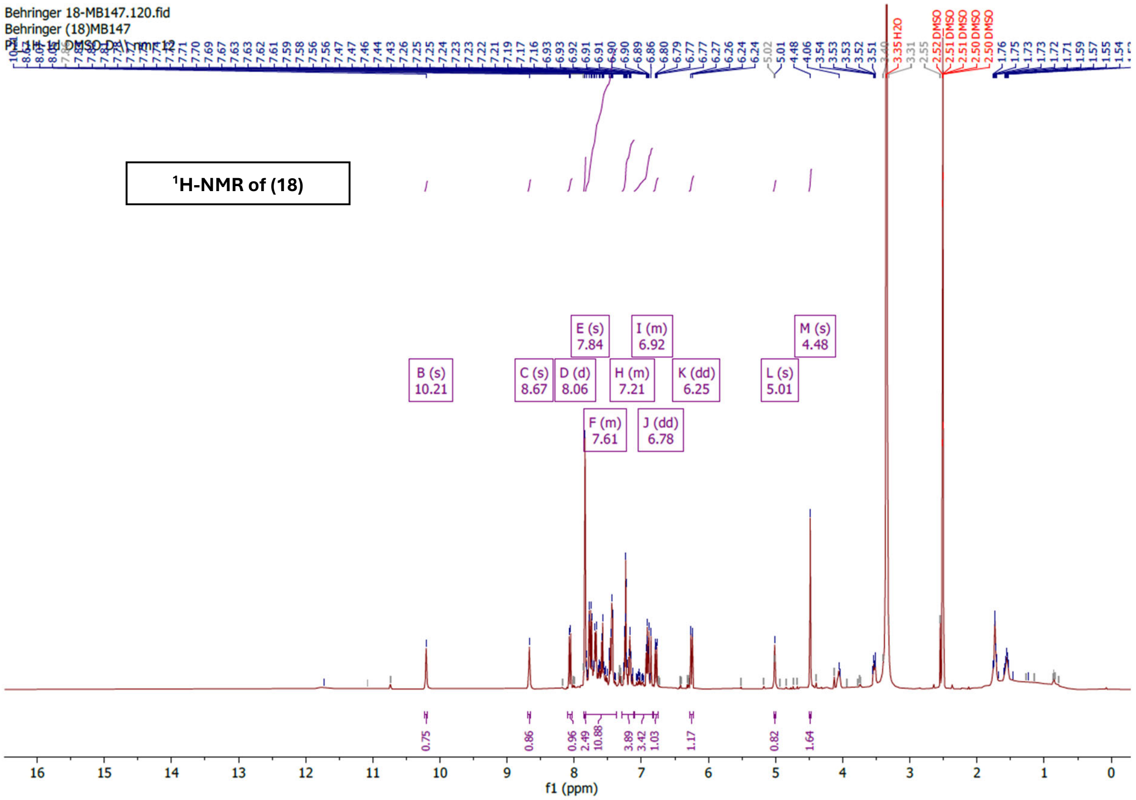

^{1}H-NMR of (18)

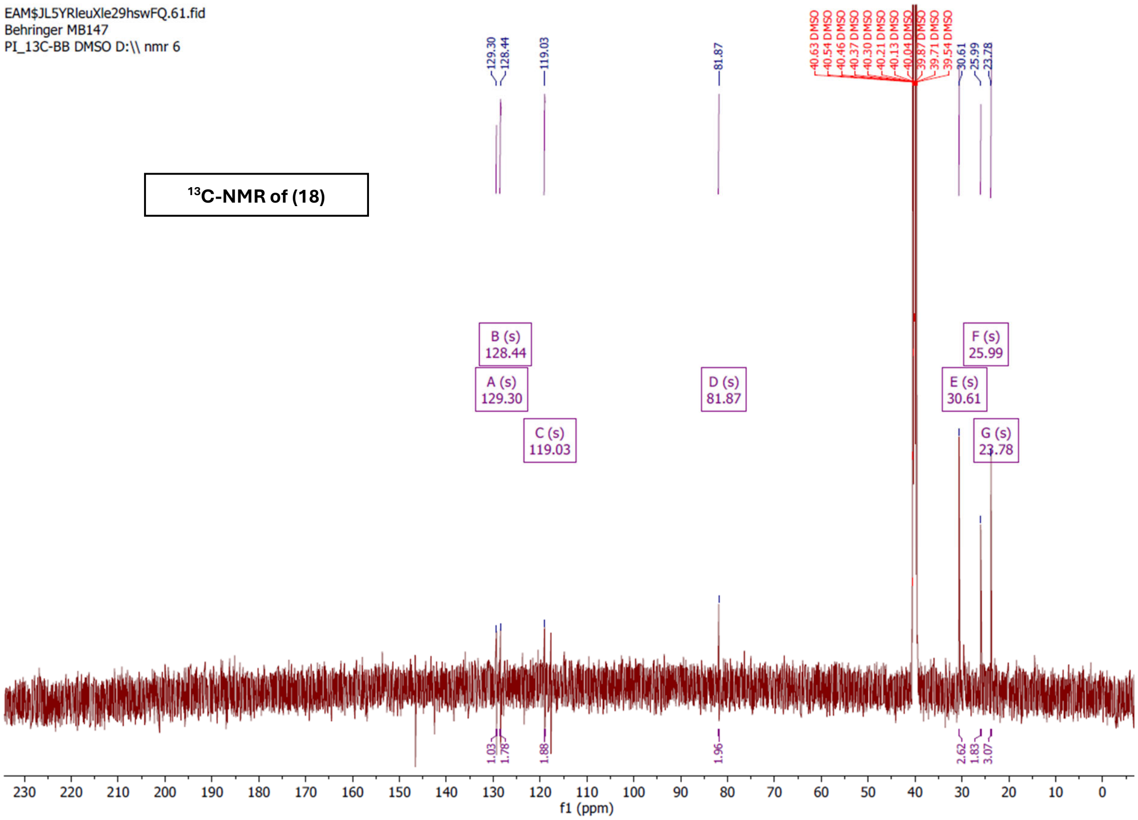
EAM$JL5YRleuXle29hswFQ.61.fid
Behringer MB147
PI_13C-BB DMSO D:\\ nmr 6
13C-NMR of (18)
129.30
128.44
119.03
81.87
40.63 DMSO
40.54 DMSO
40.46 DMSO
40.37 DMSO
40.30 DMSO
40.21 DMSO
40.13 DMSO
40.04 DMSO
39.87 DMSO
39.71 DMSO
39.54 DMSO
30.61
25.99
23.78
B (s) 128.44
A (s) 129.30
C (s) 119.03
D (s) 81.87
F (s) 25.99
E (s) 30.61
G (s) 23.78
1.03
1.78
1.88
1.96
2.62
1.83
3.07
f1 (ppm)

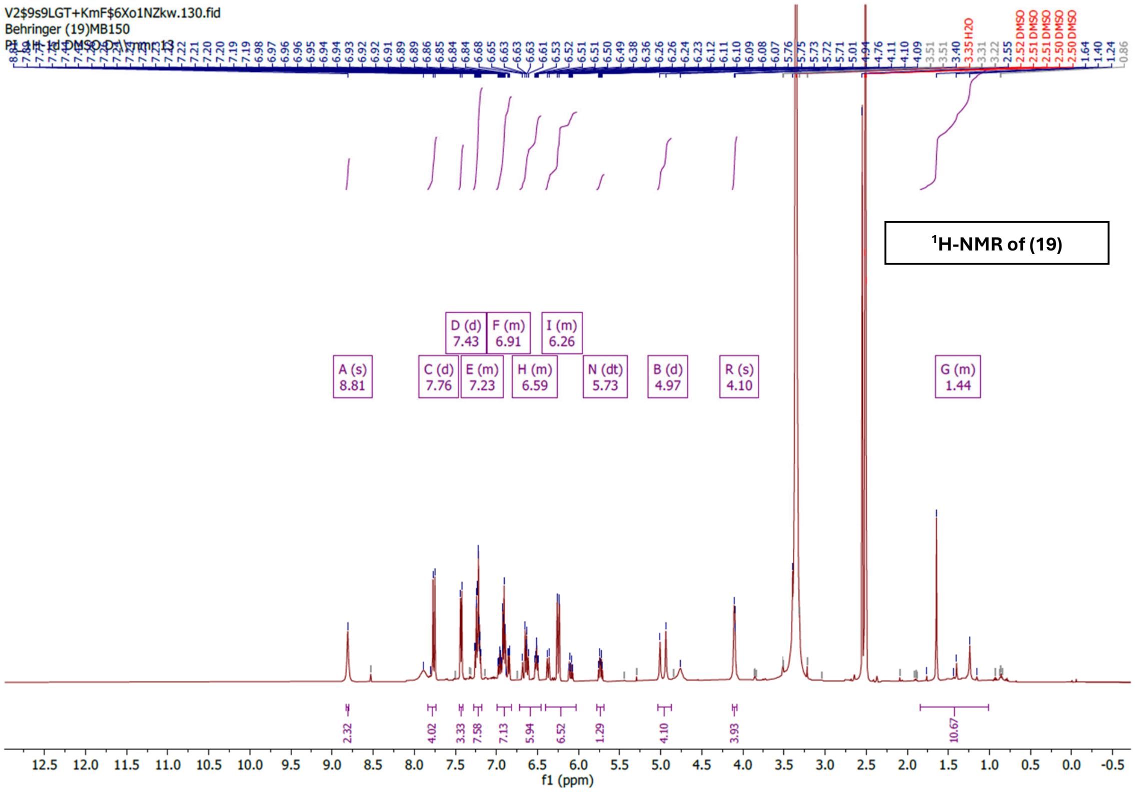
V2$9s9LGT+KmF$6Xo1NZkw.130.fid
Behringer (19)MB150
1H-NMR of (19)
3.35 H2O
2.52 DMSO
2.51 DMSO
2.51 DMSO
2.50 DMSO
2.50 DMSO
A (s) 8.81
C (d) 7.76
D (d) 7.43
E (m) 7.23
F (m) 6.91
H (m) 6.59
I (m) 6.26
N (dt) 5.73
B (d) 4.97
R (s) 4.10
G (m) 1.44
2.32
4.02
3.33
7.58
7.13
5.94
6.52
1.29
4.10
3.93
10.67
f1 (ppm)

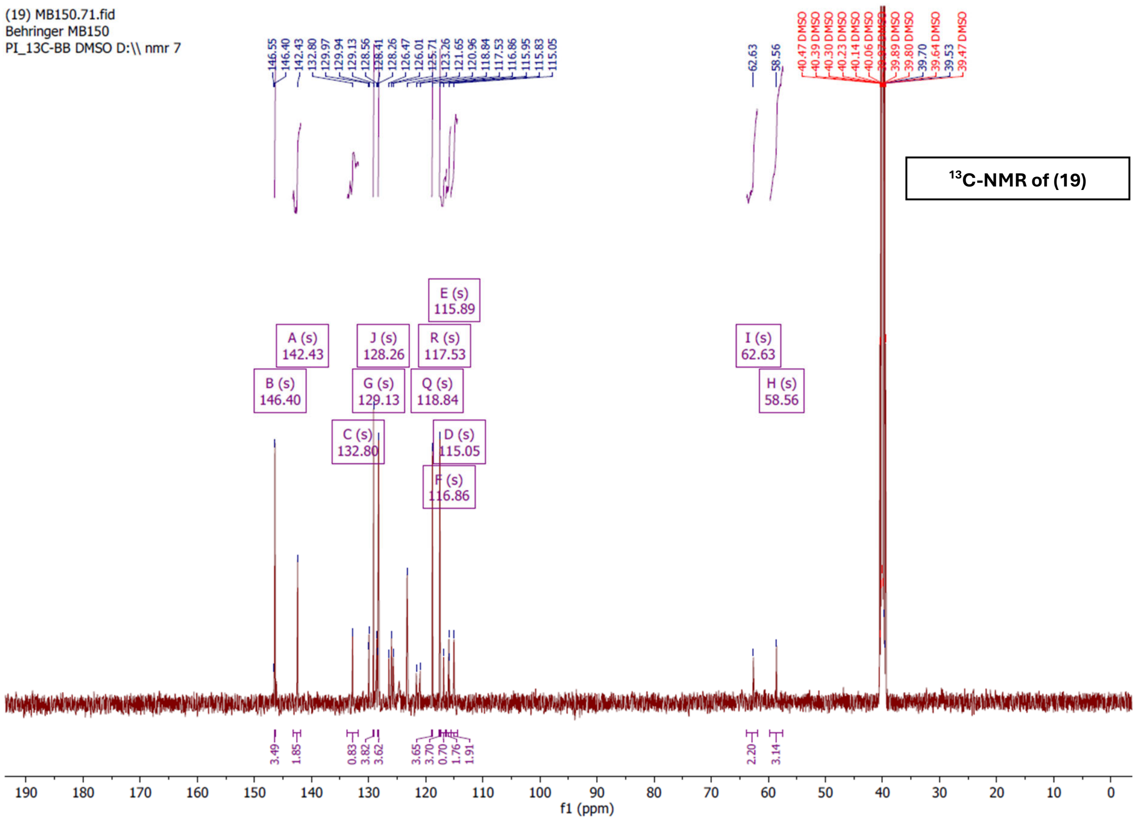
(19) MB150.71.fid
Behringer MB150
PI_13C-BB DMSO D:\\ nmr 7
13C-NMR of (19)
A (s) 142.43
B (s) 146.40
C (s) 132.80
D (s) 115.05
E (s) 115.89
F (s) 116.86
G (s) 129.13
H (s) 58.56
I (s) 62.63
J (s) 128.26
Q (s) 118.84
R (s) 117.53
f1 (ppm)

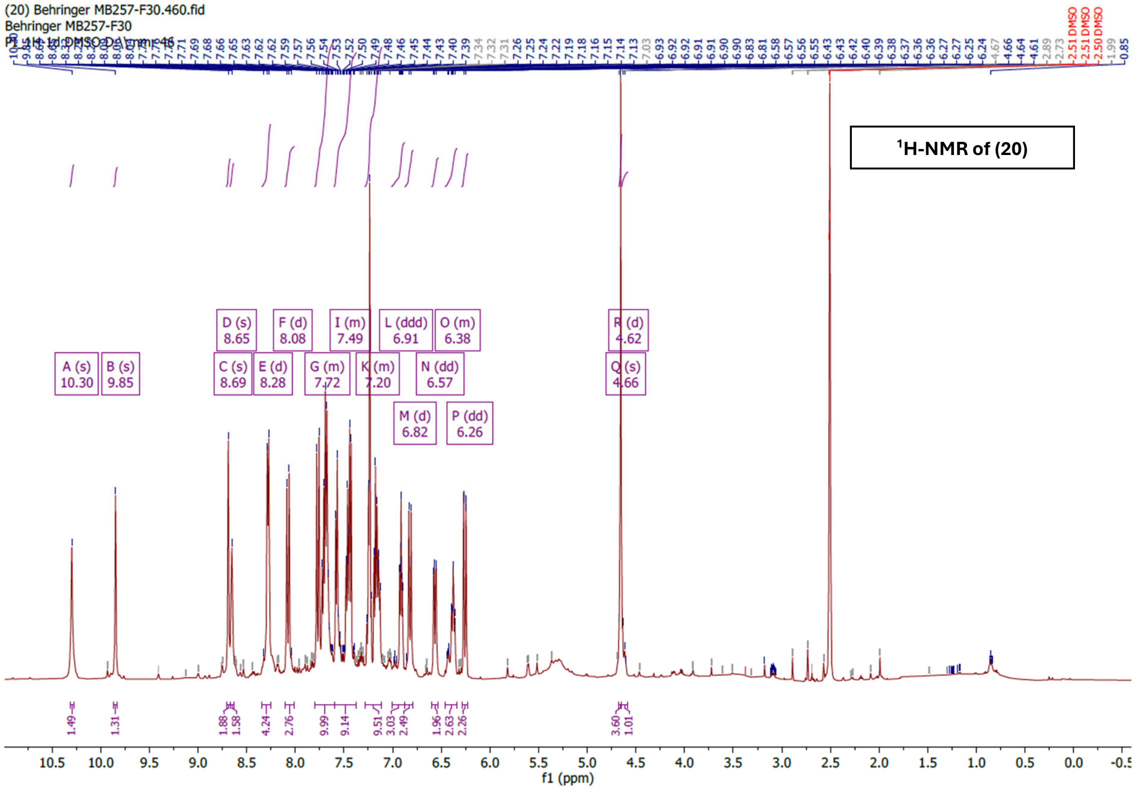
(20) Behringer MB257-F30.460.fid
Behringer MB257-F30
1H-NMR of (20)
A (s) 10.30
B (s) 9.85
C (s) 8.69
D (s) 8.65
E (d) 8.28
F (d) 8.08
G (m) 7.72
I (m) 7.49
K (m) 7.20
L (ddd) 6.91
M (d) 6.82
N (dd) 6.57
O (m) 6.38
P (dd) 6.26
Q (s) 4.66
R (d) 4.62
f1 (ppm)

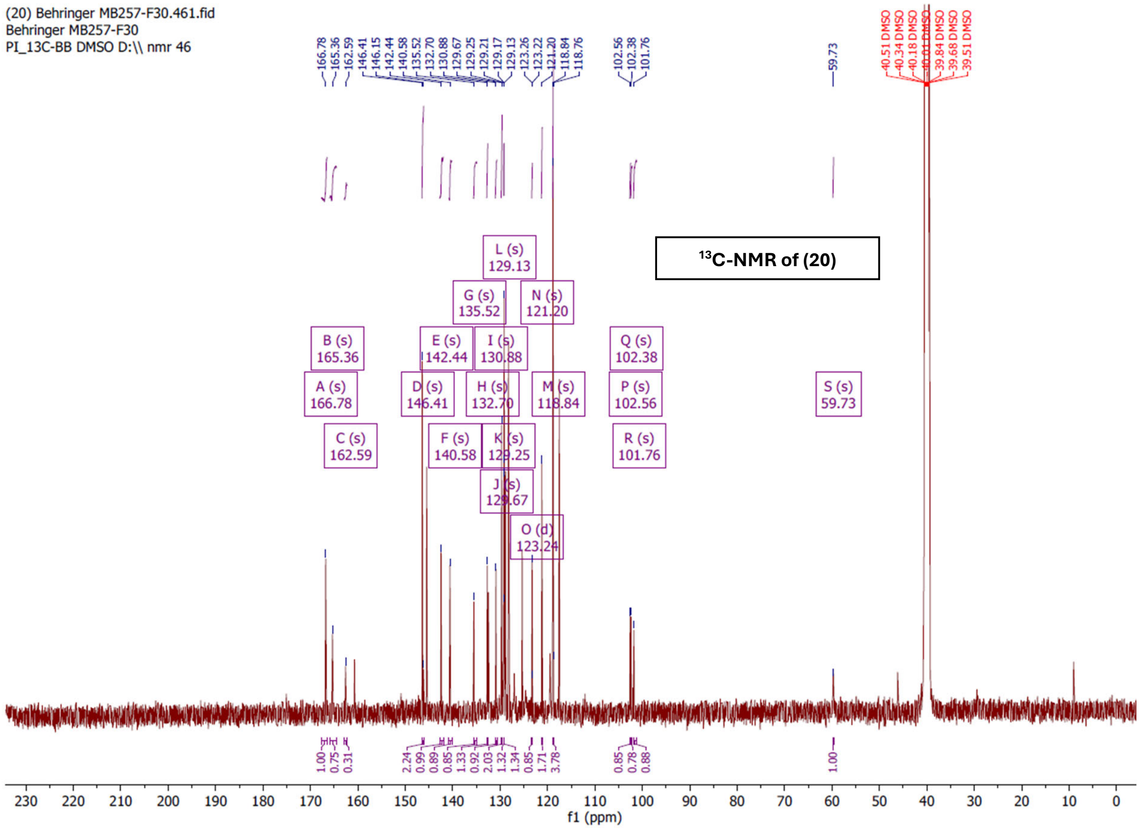
(20) Behringer MB257-F30.461.fid
Behringer MB257-F30
PI_13C-BB DMSO D:\\ nmr 46
13C-NMR of (20)
A (s) 166.78
B (s) 165.36
C (s) 162.59
D (s) 146.41
E (s) 142.44
F (s) 140.58
G (s) 135.52
H (s) 132.70
I (s) 130.88
J (s) 129.67
K (s) 129.25
L (s) 129.13
M (s) 118.84
N (s) 121.20
O (d) 123.24
P (s) 102.56
Q (s) 102.38
R (s) 101.76
S (s) 59.73
f1 (ppm)

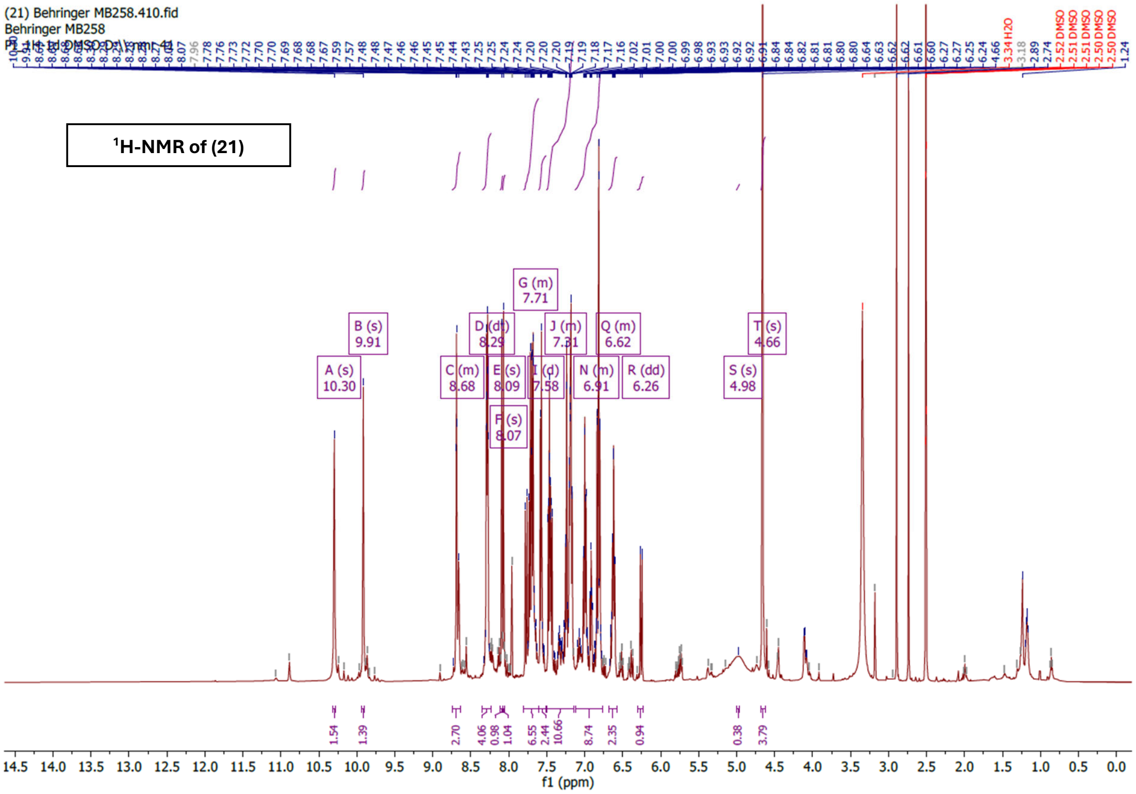
(21) Behringer MB258.410.fid
Behringer MB258
1H-NMR of (21)
A (s) 10.30
B (s) 9.91
C (m) 8.68
D (dt) 8.29
E (s) 8.09
F (s) 8.07
G (m) 7.71
I (d) 7.58
J (m) 7.31
N (m) 6.91
Q (m) 6.62
R (dd) 6.26
S (s) 4.98
T (s) 4.66
f1 (ppm)

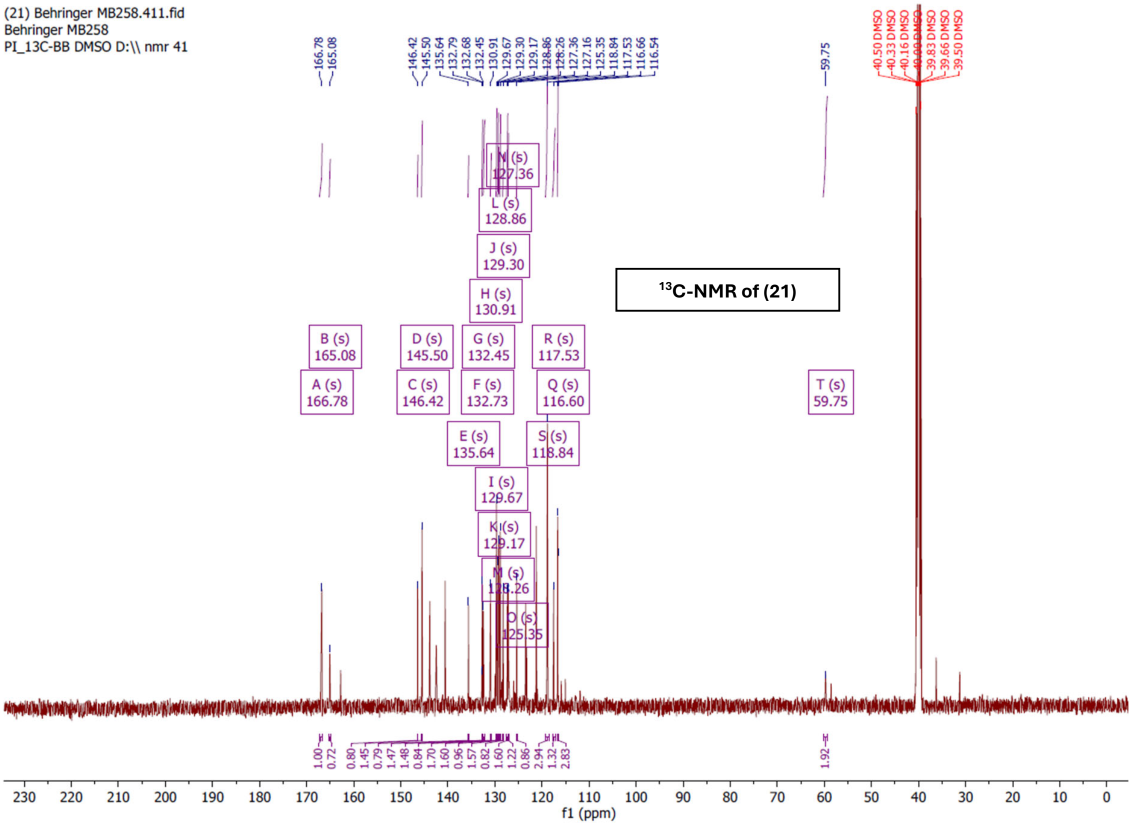
(21) Behringer MB258.411.fid
Behringer MB258
PI_13C-BB DMSO D:\\ nmr 41
13C-NMR of (21)
A (s) 166.78
B (s) 165.08
C (s) 146.42
D (s) 145.50
E (s) 135.64
F (s) 132.73
G (s) 132.45
H (s) 130.91
I (s) 129.67
J (s) 129.30
K (s) 129.17
L (s) 128.86
M (s) 128.26
N (s) 127.36
O (s) 125.35
Q (s) 116.60
R (s) 117.53
S (s) 118.84
T (s) 59.75
f1 (ppm)

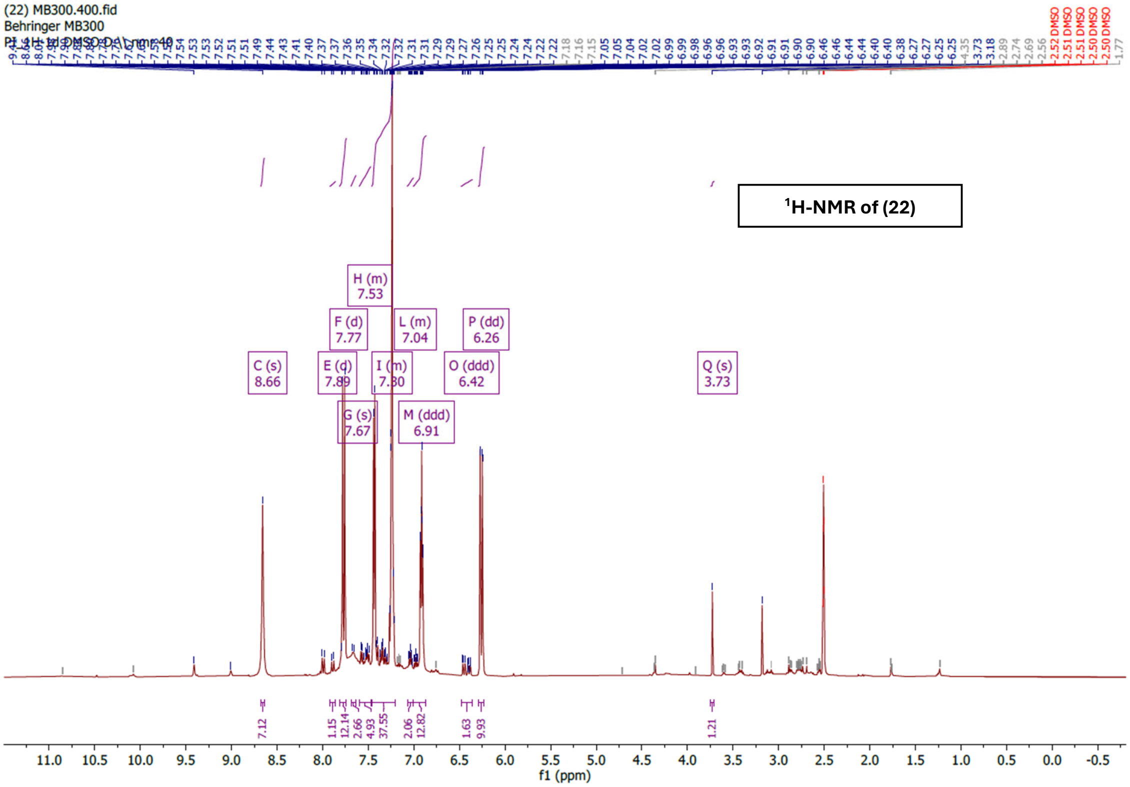
(22) MB300.400.fid
Behringer MB300
¹H-NMR of (22)
C (s) 8.66
E (d) 7.89
F (d) 7.77
G (s) 7.67
H (m) 7.53
I (m) 7.30
L (m) 7.04
M (ddd) 6.91
O (ddd) 6.42
P (dd) 6.26
Q (s) 3.73
7.12
1.15
12.14
2.66
4.93
37.55
2.06
12.82
1.63
9.93
1.21
f1 (ppm)

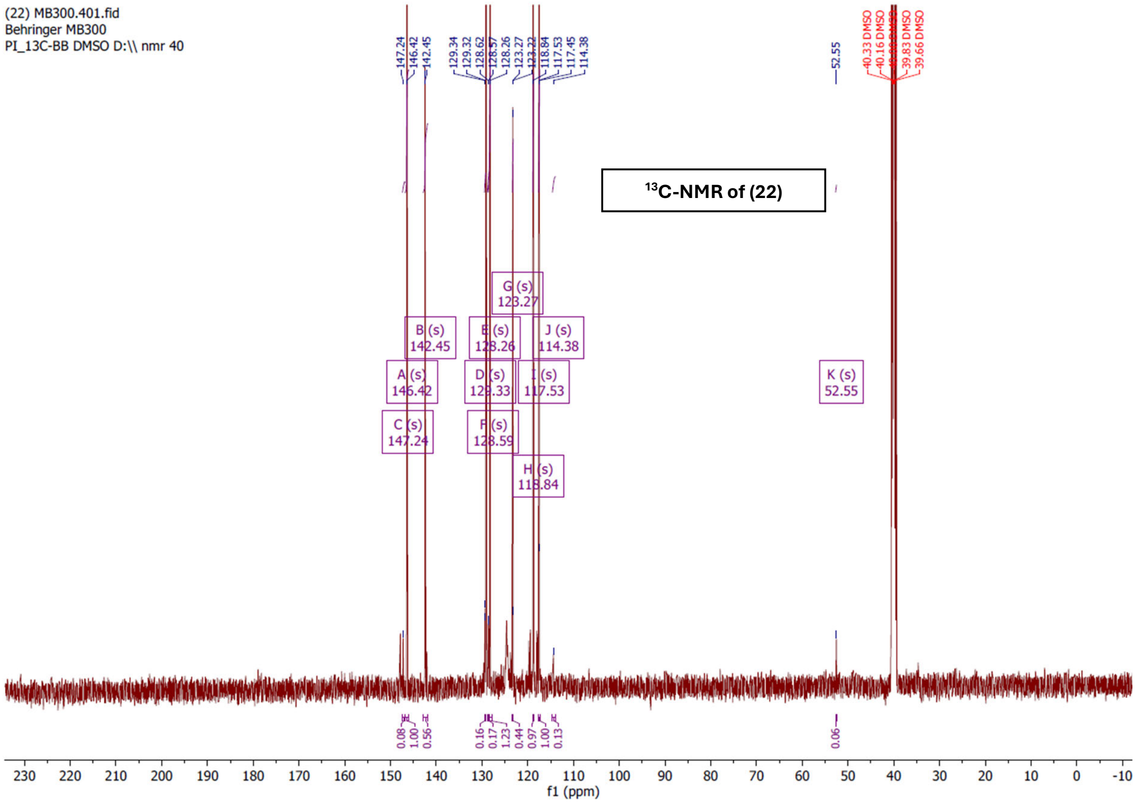
(22) MB300.401.fid
Behringer MB300
PI_13C-BB DMSO D:\\ nmr 40
¹³C-NMR of (22)
A (s) 146.42
B (s) 142.45
C (s) 147.24
D (s) 129.33
E (s) 128.26
F (s) 128.59
G (s) 123.27
H (s) 118.84
I (s) 117.53
J (s) 114.38
K (s) 52.55
40.33 DMSO
40.16 DMSO
39.83 DMSO
39.66 DMSO
f1 (ppm)

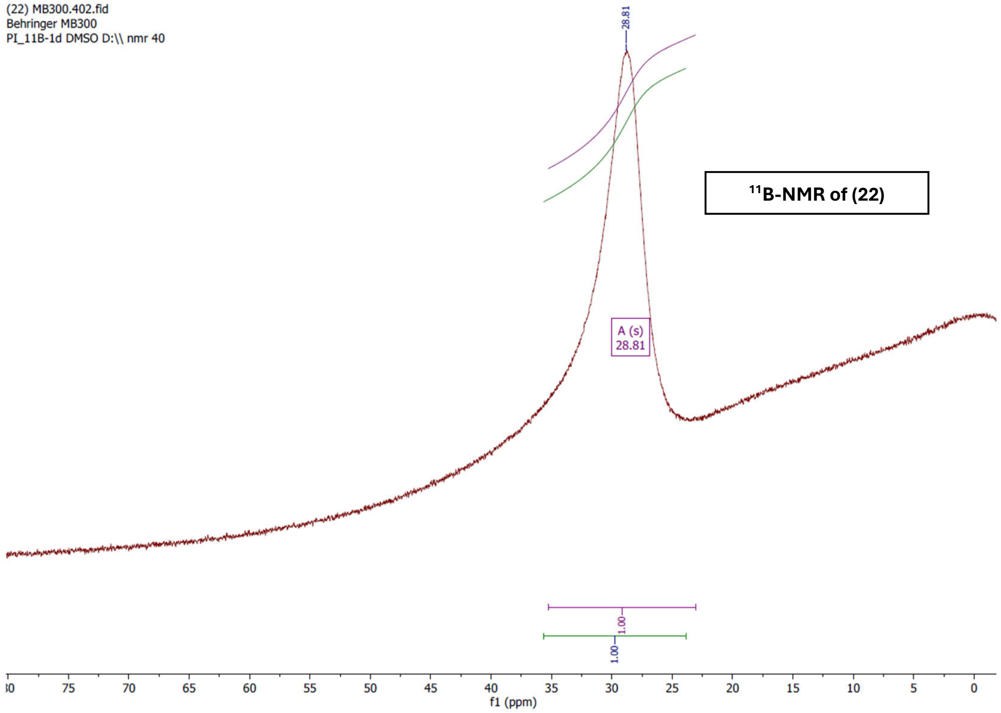

^{11}B-NMR of (22)

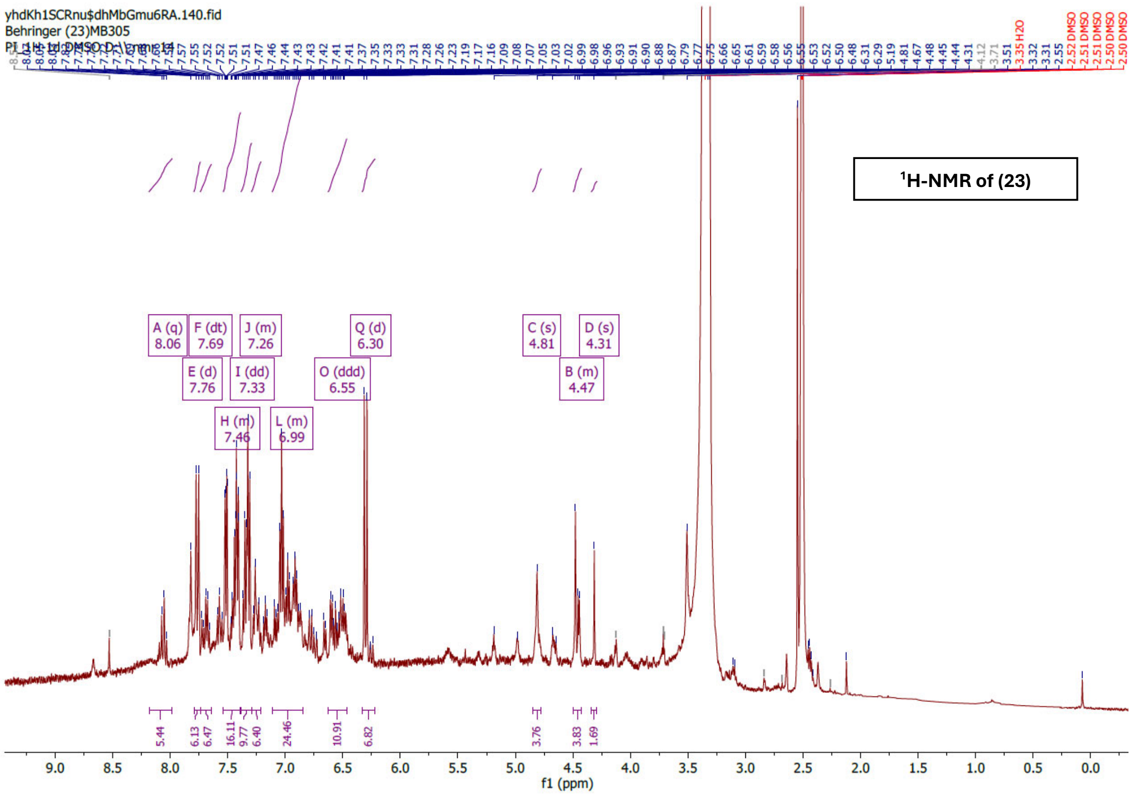

^{1}H-NMR of (23)

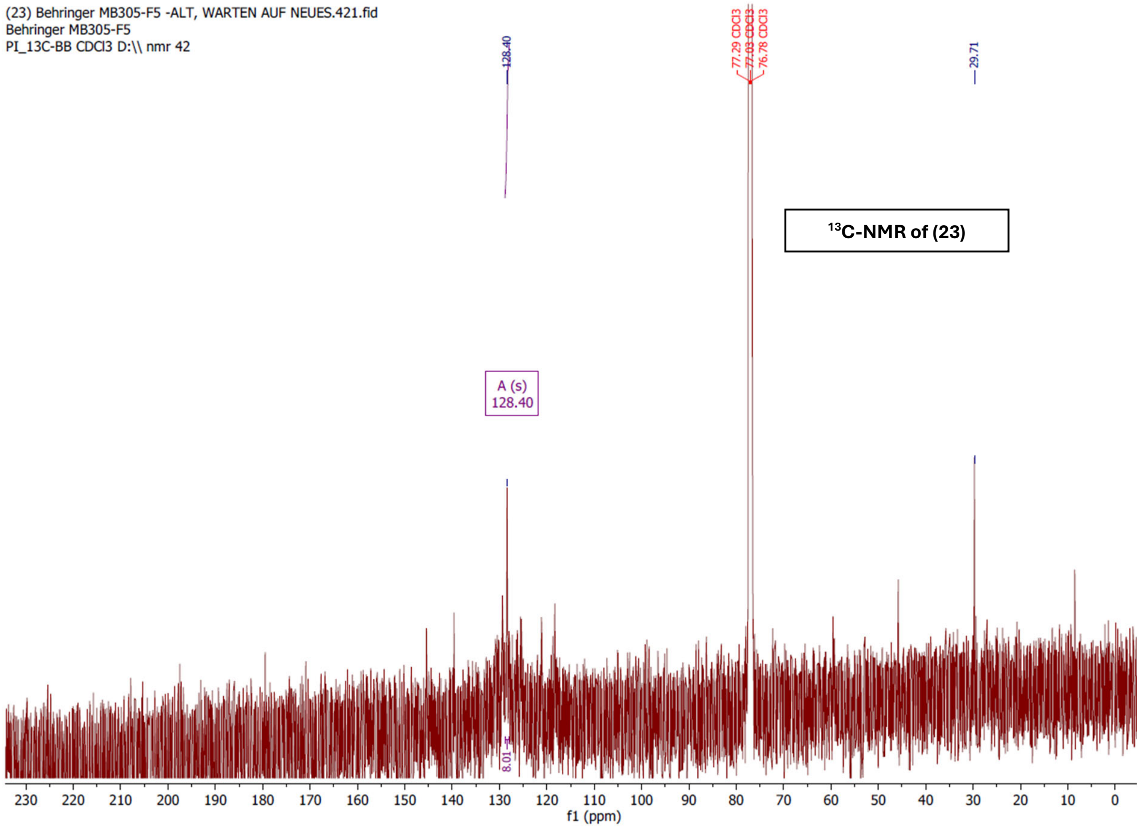

(23) Behringer MB305-F5 -ALT, WARTEN AUF NEUES.421.fid
Behringer MB305-F5
PI_13C-BB CDCl3 D:\\ nmr 42
128.40
77.29 CDCl3
77.03 CDCl3
76.78 CDCl3
29.71
13C-NMR of (23)
A (s)
128.40
8.01
230 220 210 200 190 180 170 160 150 140 130 120 110 100 90 80 70 60 50 40 30 20 10 0
f1 (ppm)

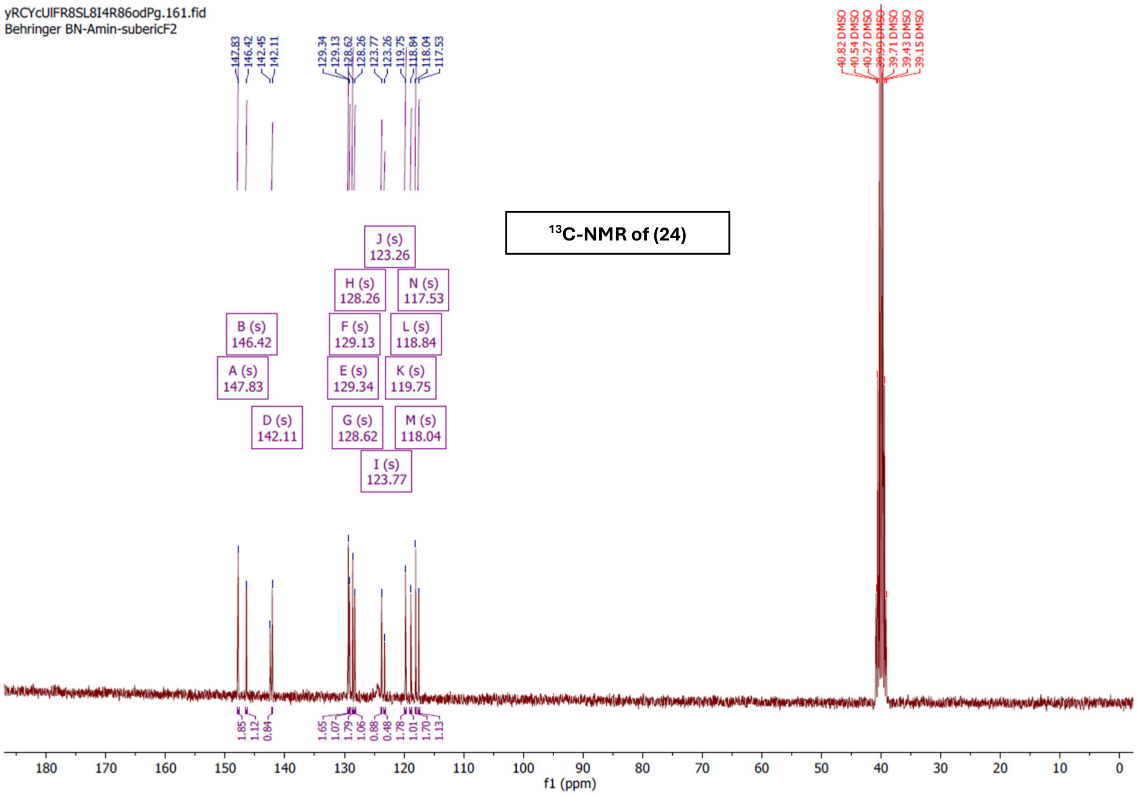

^{13}C-NMR of (24)

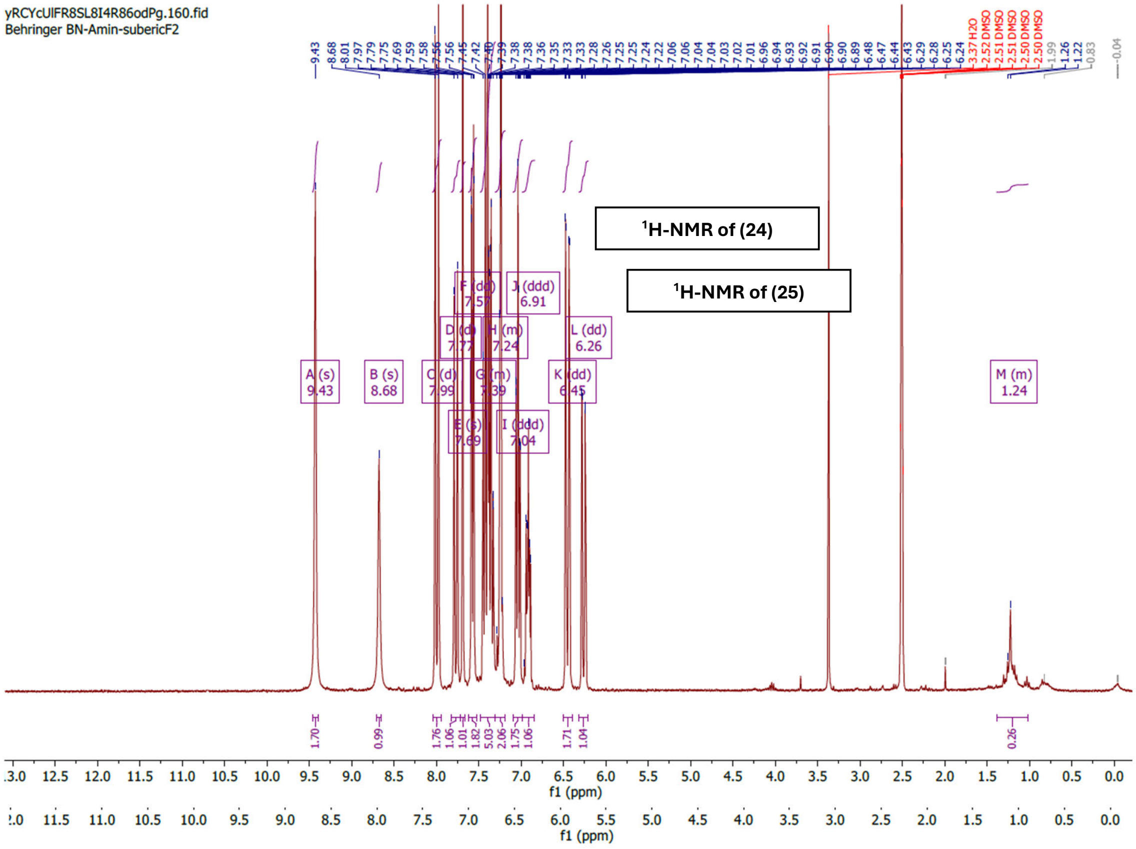

^{1}H-NMR of (24)

^{1}H-NMR of (25)

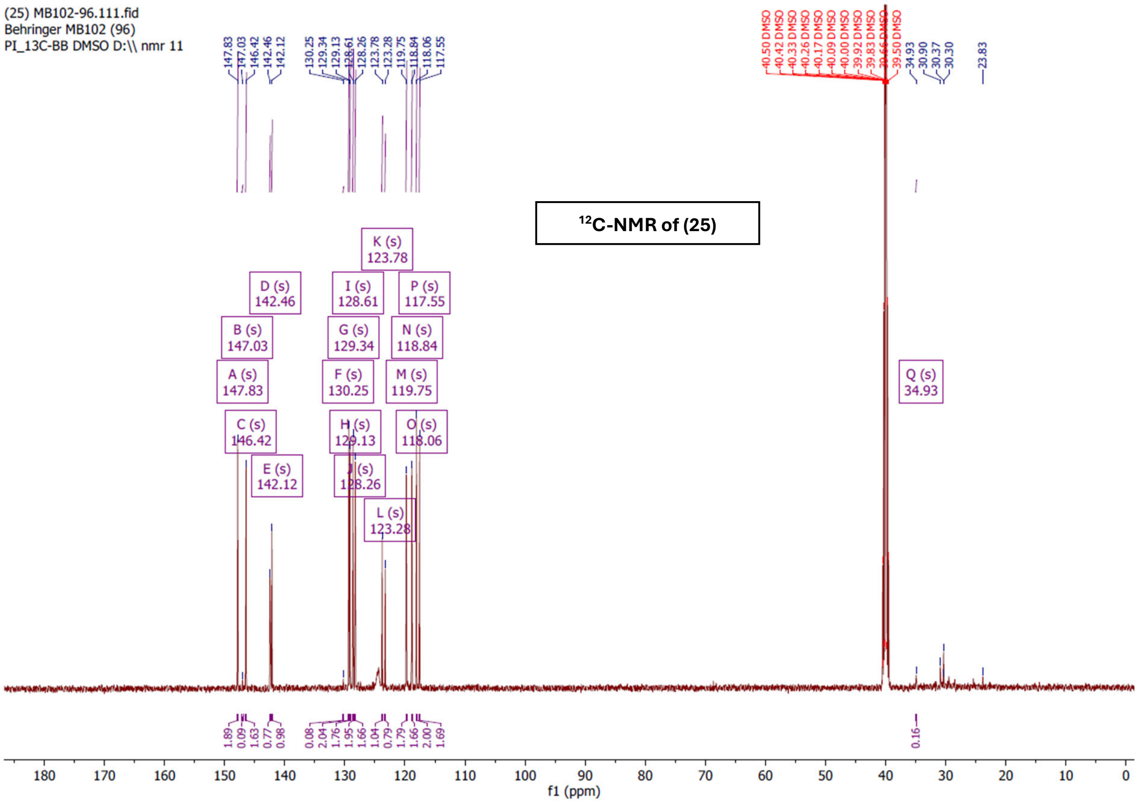

[12]C-NMR of (25)

^{11}B-NMR of (25)

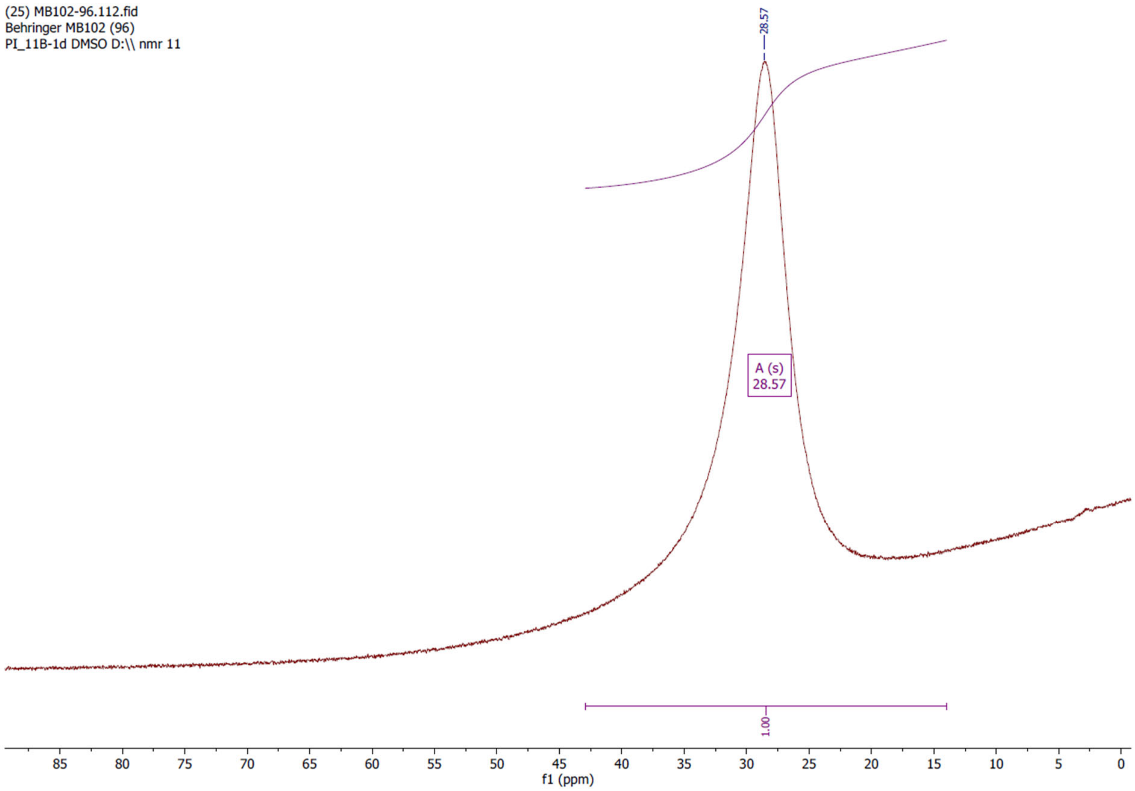
(25) MB102-96.112.fid
Behringer MB102 (96)
PI_11B-1d DMSO D:\\ nmr 11
28.57
A (s)
28.57
1.00
85 80 75 70 65 60 55 50 45 40 35 30 25 20 15 10 5 0
f1 (ppm)

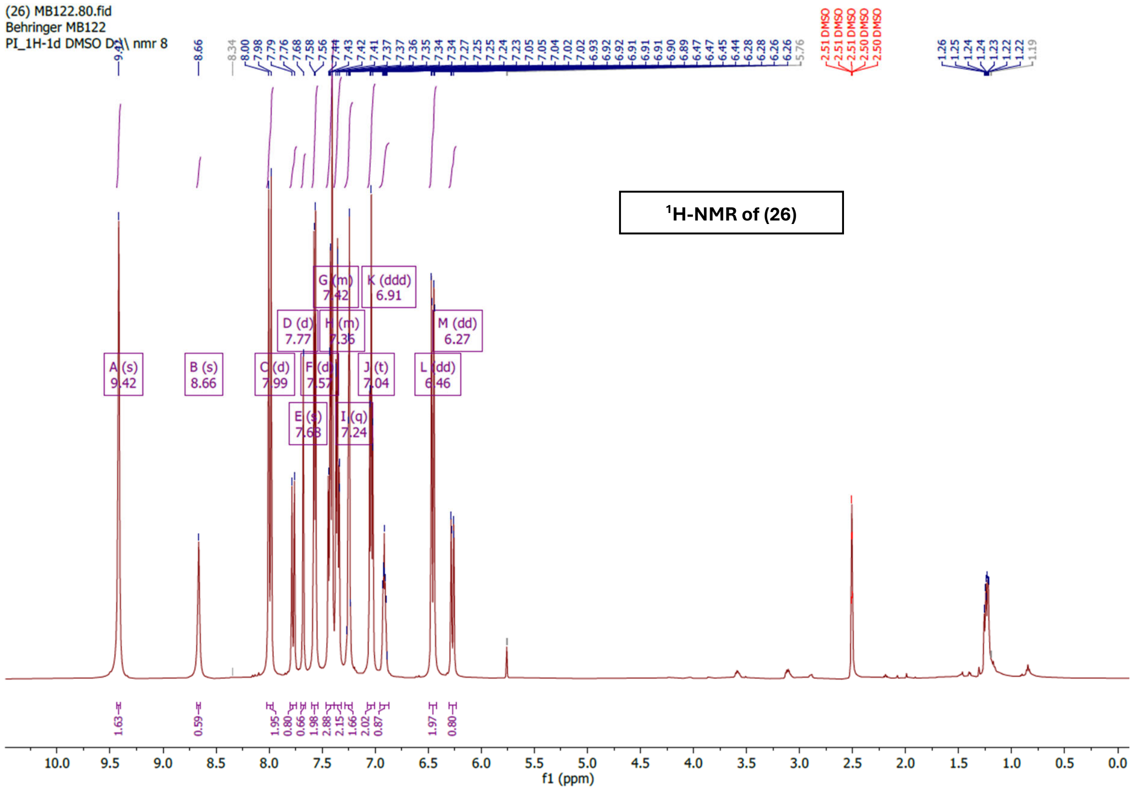
(26) MB122.80.fid
Behringer MB122
PI_1H-1d DMSO D:\\ nmr 8
1H-NMR of (26)
A (s) 9.42
B (s) 8.66
C (d) 7.99
D (d) 7.77
E (s) 7.68
F (d) 7.57
G (m) 7.42
H (m) 7.36
I (q) 7.24
J (t) 7.04
K (ddd) 6.91
L (dd) 6.46
M (dd) 6.27
f1 (ppm)

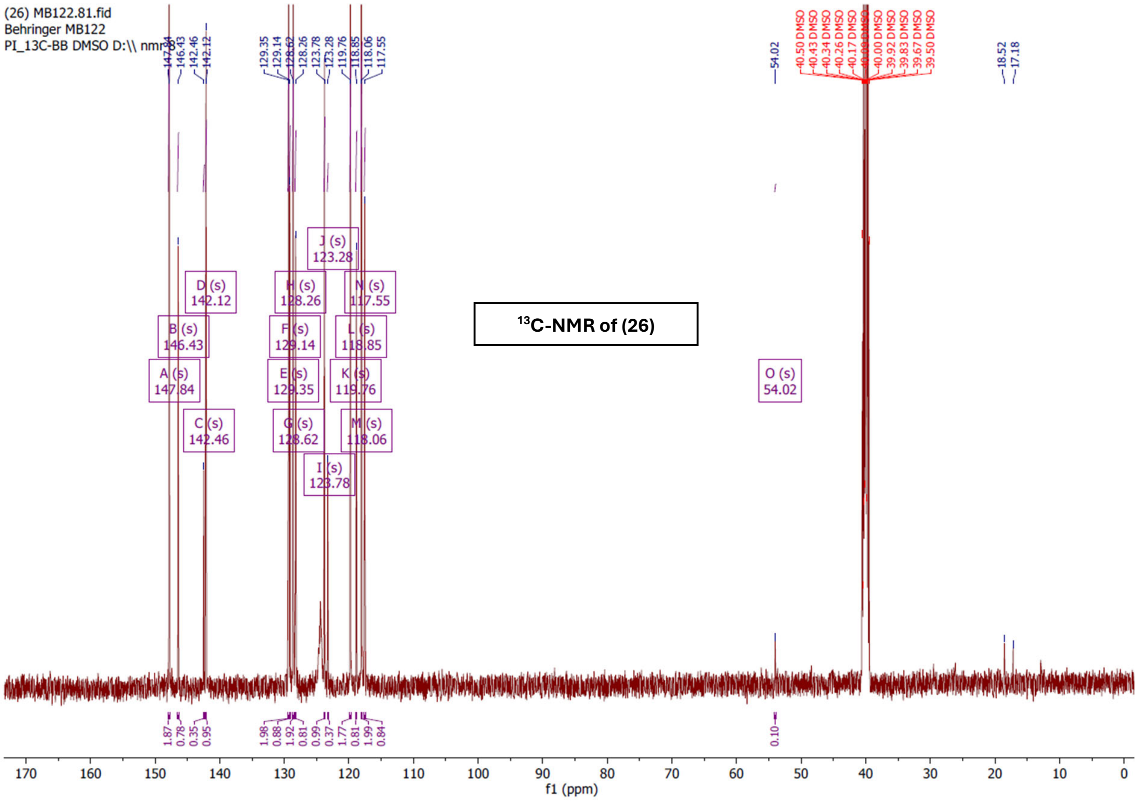
(26) MB122.81.fid
Behringer MB122
PI_13C-BB DMSO D:\\ nmr 8
13C-NMR of (26)
A (s) 147.84
B (s) 146.43
C (s) 142.46
D (s) 142.12
E (s) 129.35
F (s) 129.14
G (s) 128.62
H (s) 128.26
I (s) 123.78
J (s) 123.28
K (s) 119.76
L (s) 118.85
M (s) 118.06
N (s) 117.55
O (s) 54.02
f1 (ppm)

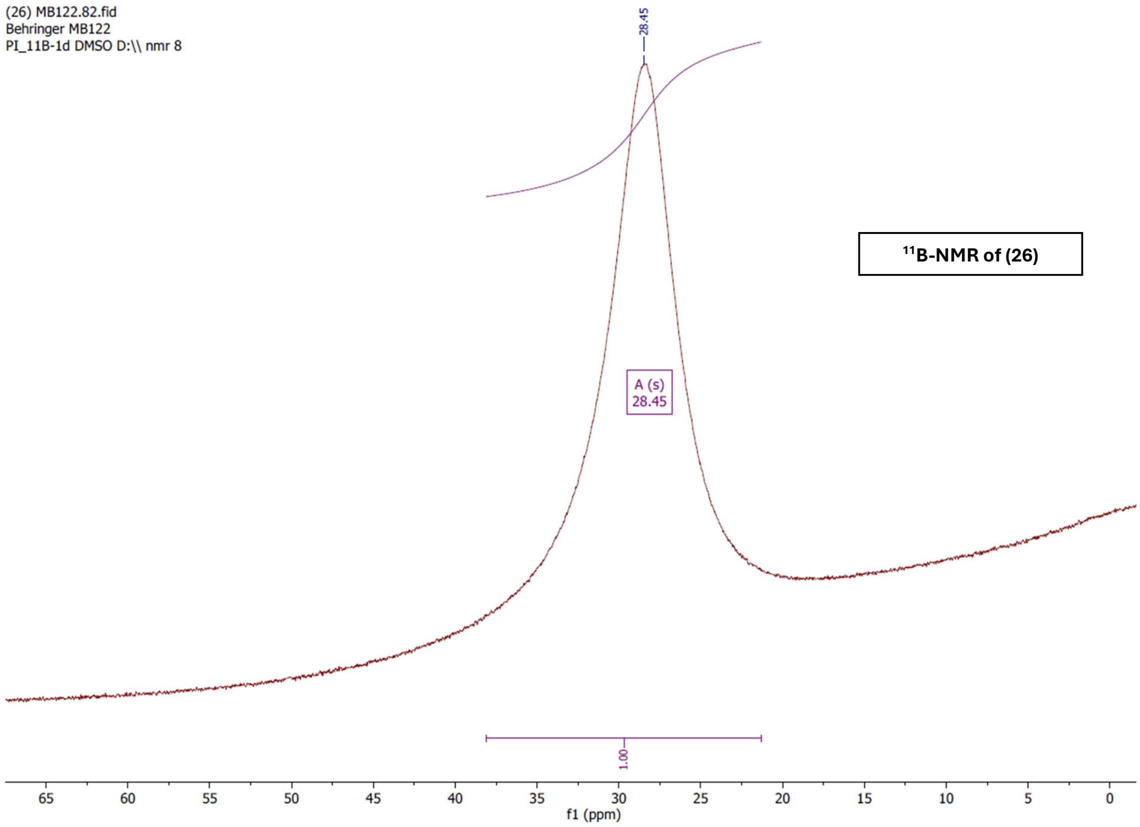
(26) MB122.82.fid
Behringer MB122
PI_11B-1d DMSO D:\\ nmr 8
28.45
11B-NMR of (26)
A (s)
28.45
1.00
f1 (ppm)

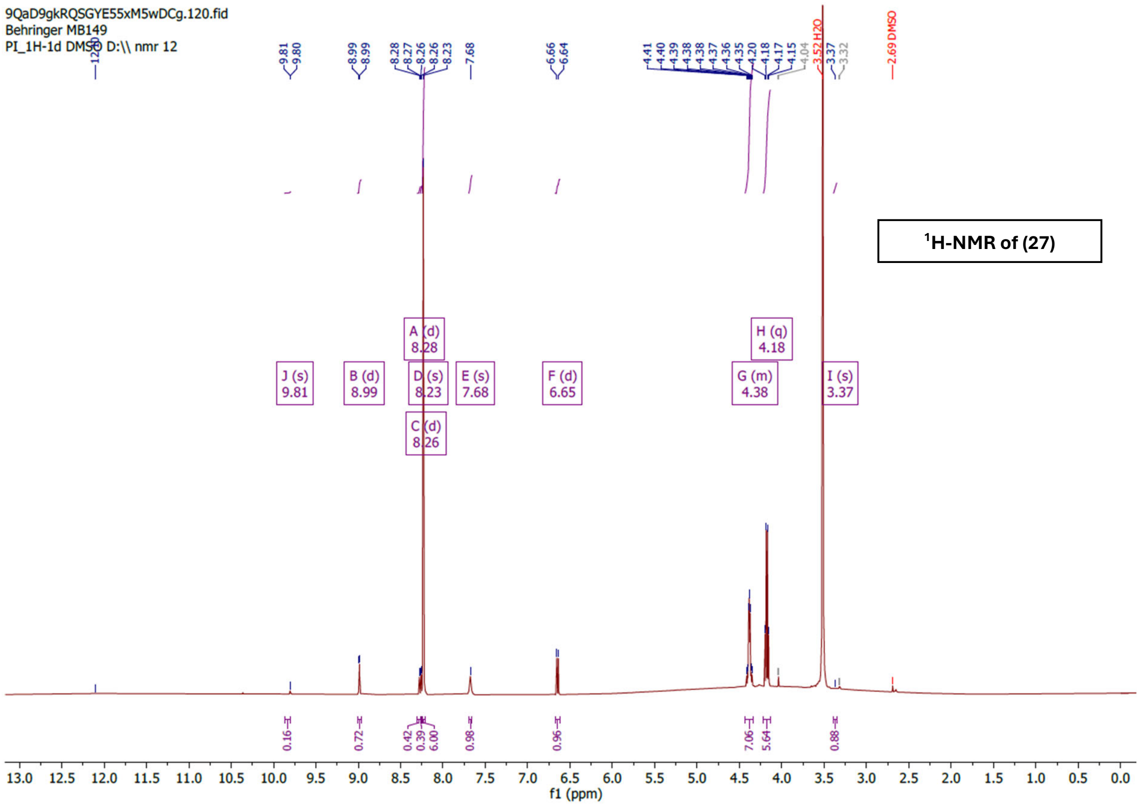
9QaD9gkRQSGYE55xM5wDCg.120.fid
Behringer MB149
PI_1H-1d DMSO D:\\ nmr 12
1H-NMR of (27)
A (d) 8.28
B (d) 8.99
C (d) 8.26
D (s) 8.23
E (s) 7.68
F (d) 6.65
G (m) 4.38
H (q) 4.18
I (s) 3.37
J (s) 9.81
3.52 H2O
2.69 DMSO
f1 (ppm)

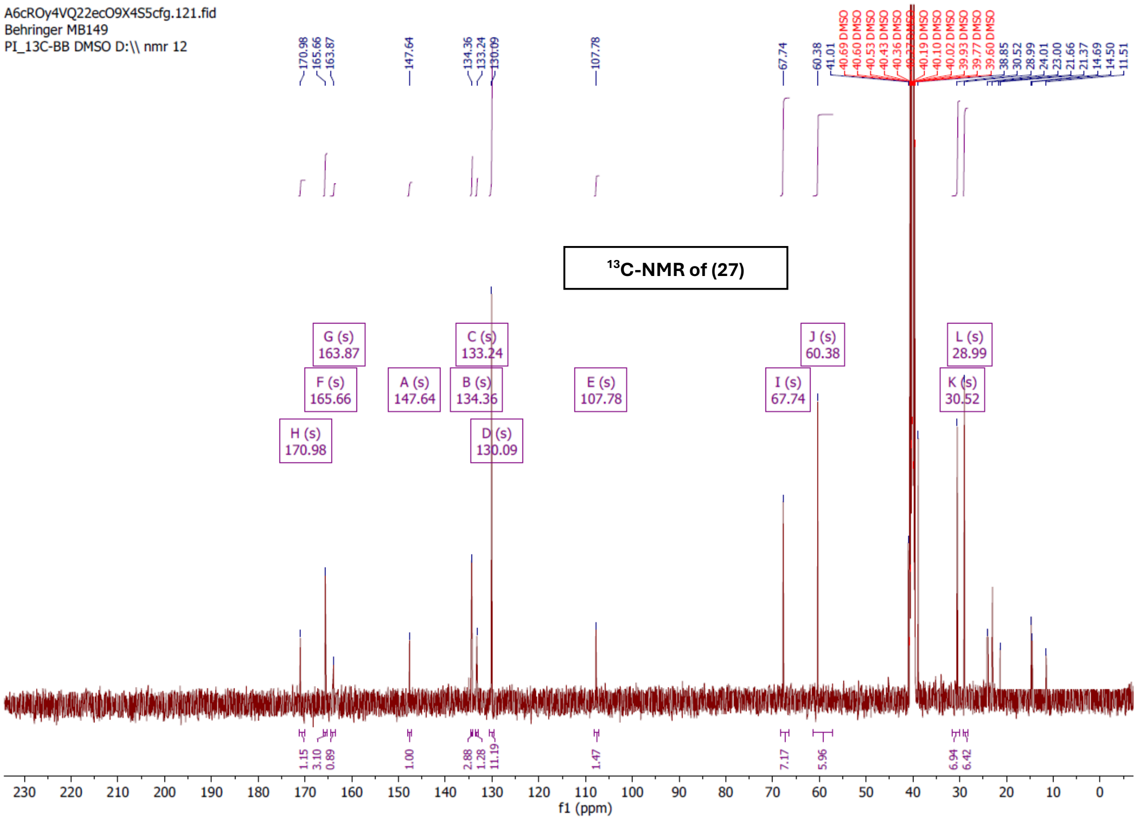
A6cROy4VQ22ecO9X4S5cfg.121.fid
Behringer MB149
PI_13C-BB DMSO D:\\ nmr 12
^{13}C-NMR of (27)
f1 (ppm)

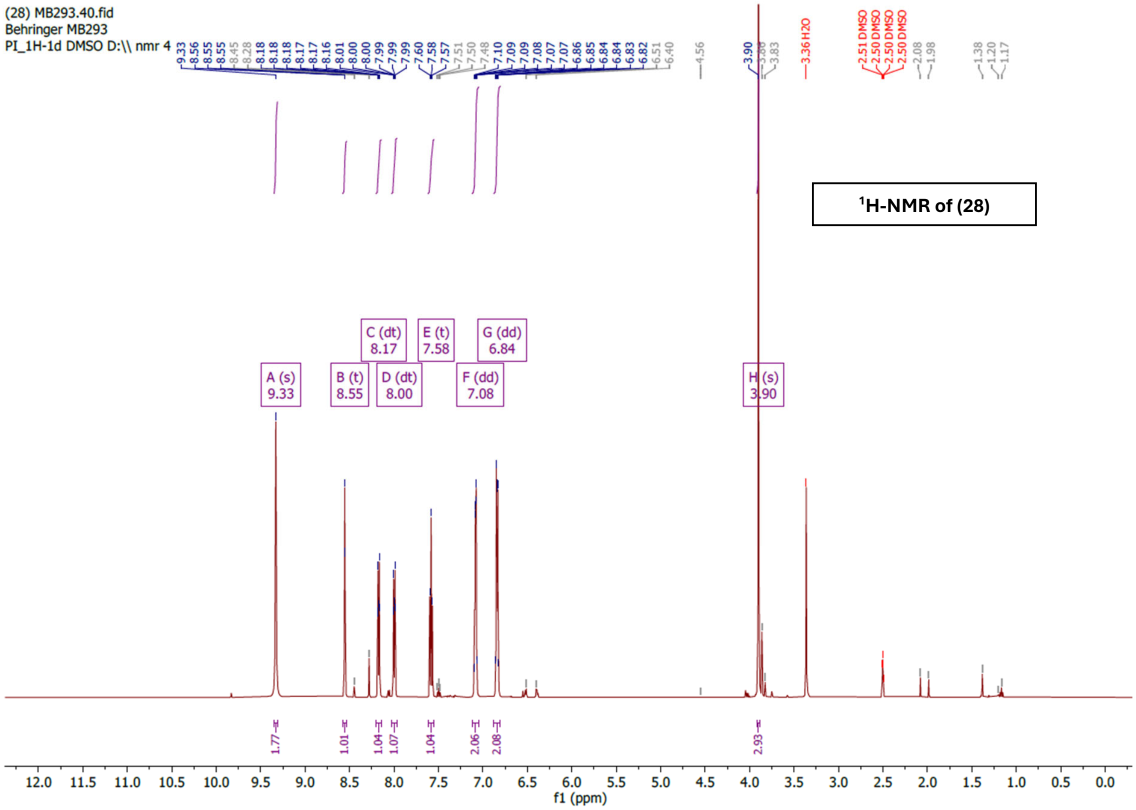
(28) MB293.40.fid
Behringer MB293
PI_1H-1d DMSO D:\\ nmr 4
^{1}H-NMR of (28)
f1 (ppm)

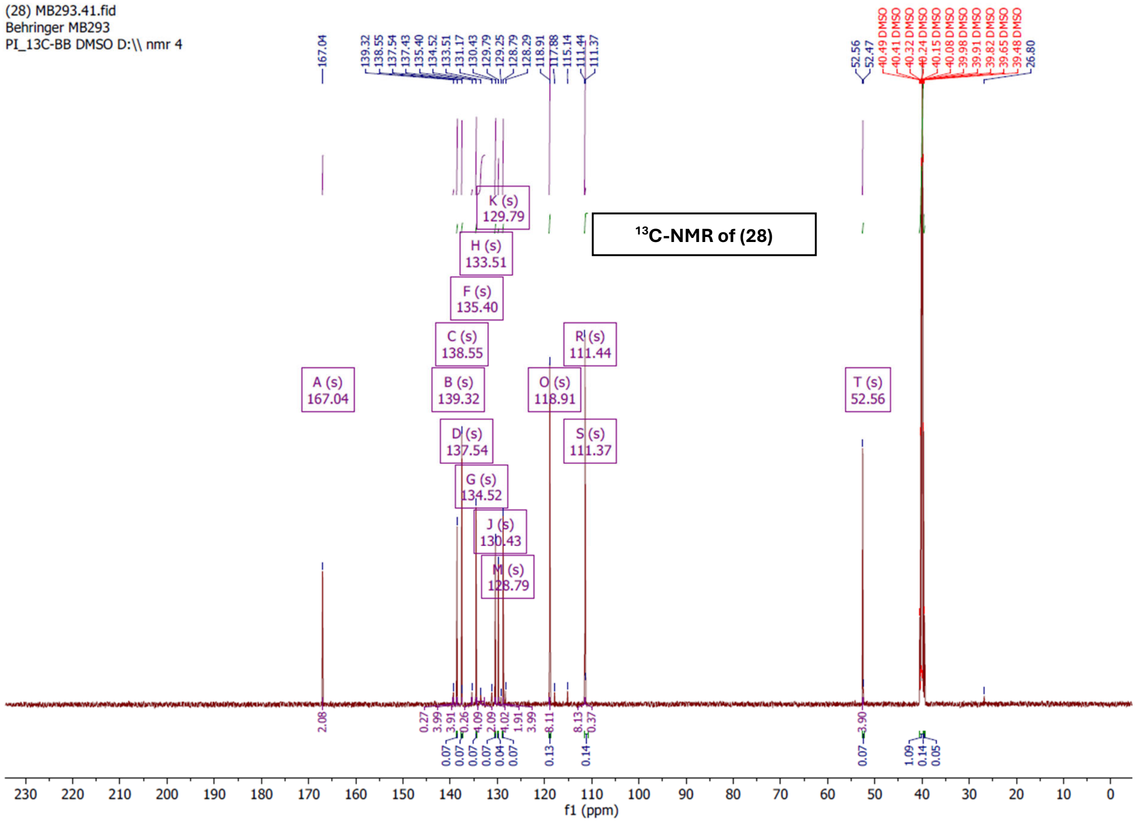
(28) MB293.41.fid
Behringer MB293
PI_13C-BB DMSO D:\\ nmr 4
^{13}C-NMR of (28)
A (s) 167.04
B (s) 139.32
C (s) 138.55
D (s) 137.54
F (s) 135.40
G (s) 134.52
H (s) 133.51
J (s) 130.43
K (s) 129.79
M (s) 128.79
O (s) 118.91
R (s) 111.44
S (s) 111.37
T (s) 52.56
f1 (ppm)

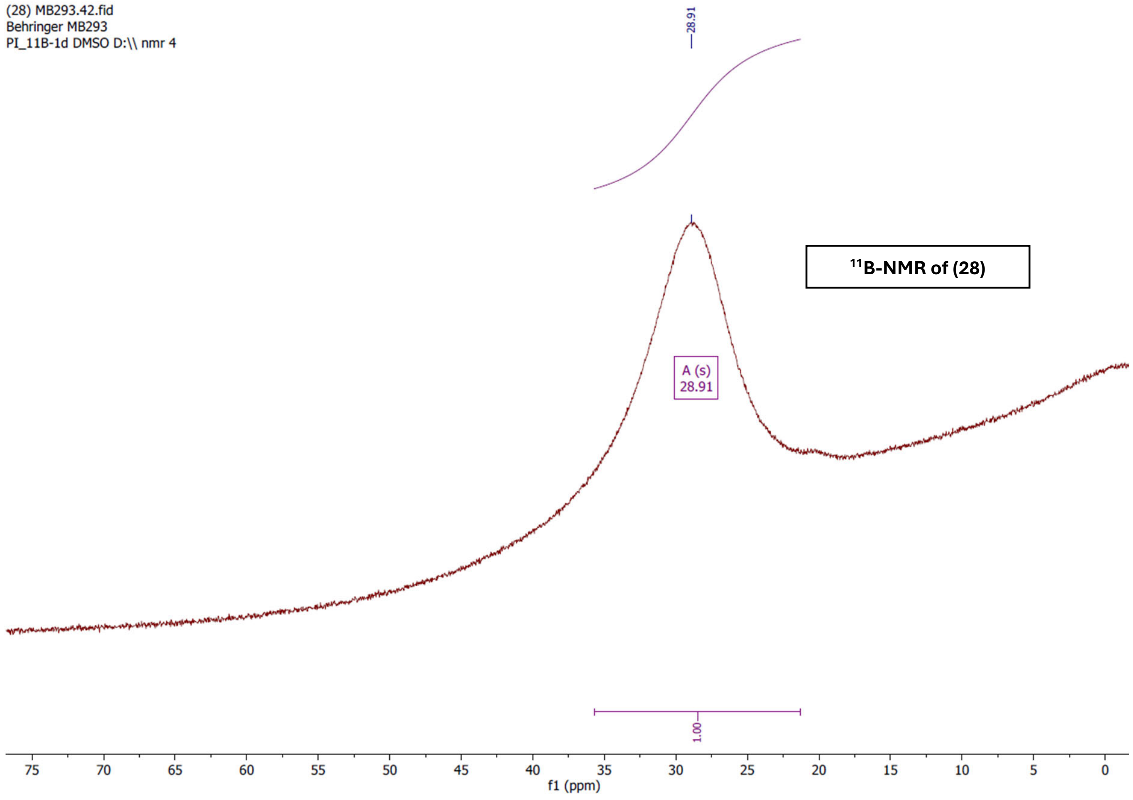
(28) MB293.42.fid
Behringer MB293
PI_11B-1d DMSO D:\\ nmr 4
^{11}B-NMR of (28)
A (s) 28.91
f1 (ppm)

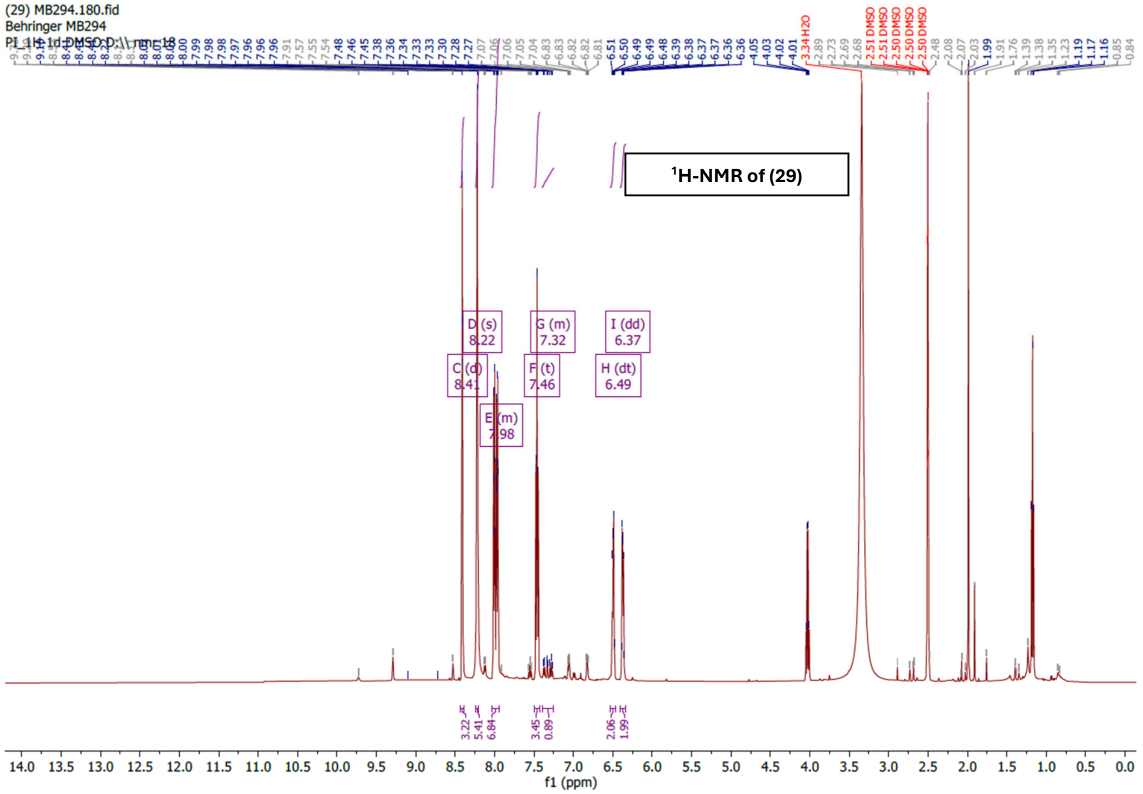
(29) MB294.180.fid
Behringer MB294
PI_1H 1d DMSO D:\\ nmr 18
¹H-NMR of (29)
D (s) 8.22
C (d) 8.41
E (m) 7.98
G (m) 7.32
F (t) 7.46
I (dd) 6.37
H (dt) 6.49
3.34 H2O
2.51 DMSO
2.50 DMSO
f1 (ppm)

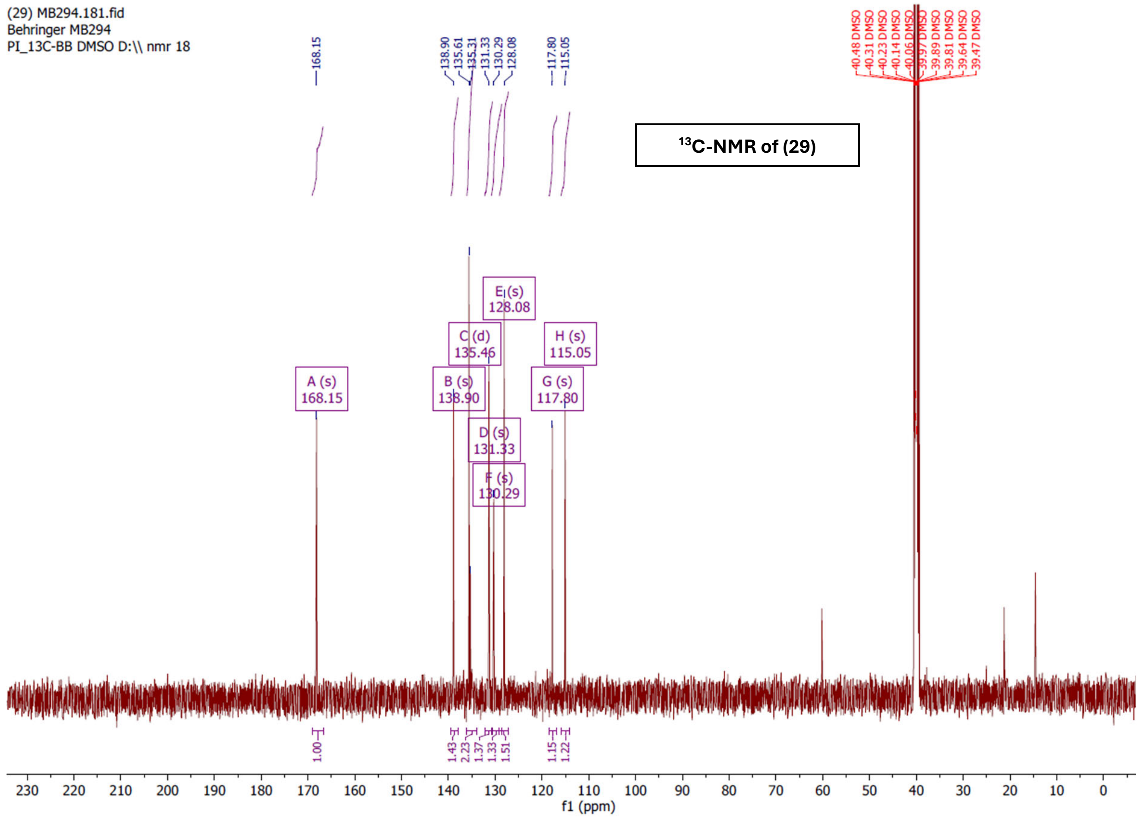
(29) MB294.181.fid
Behringer MB294
PI_13C-BB DMSO D:\\ nmr 18
¹³C-NMR of (29)
A (s) 168.15
B (s) 138.90
C (d) 135.46
D (s) 131.33
E (s) 128.08
F (s) 130.29
G (s) 117.80
H (s) 115.05
f1 (ppm)

^{1}H-NMR of (30)

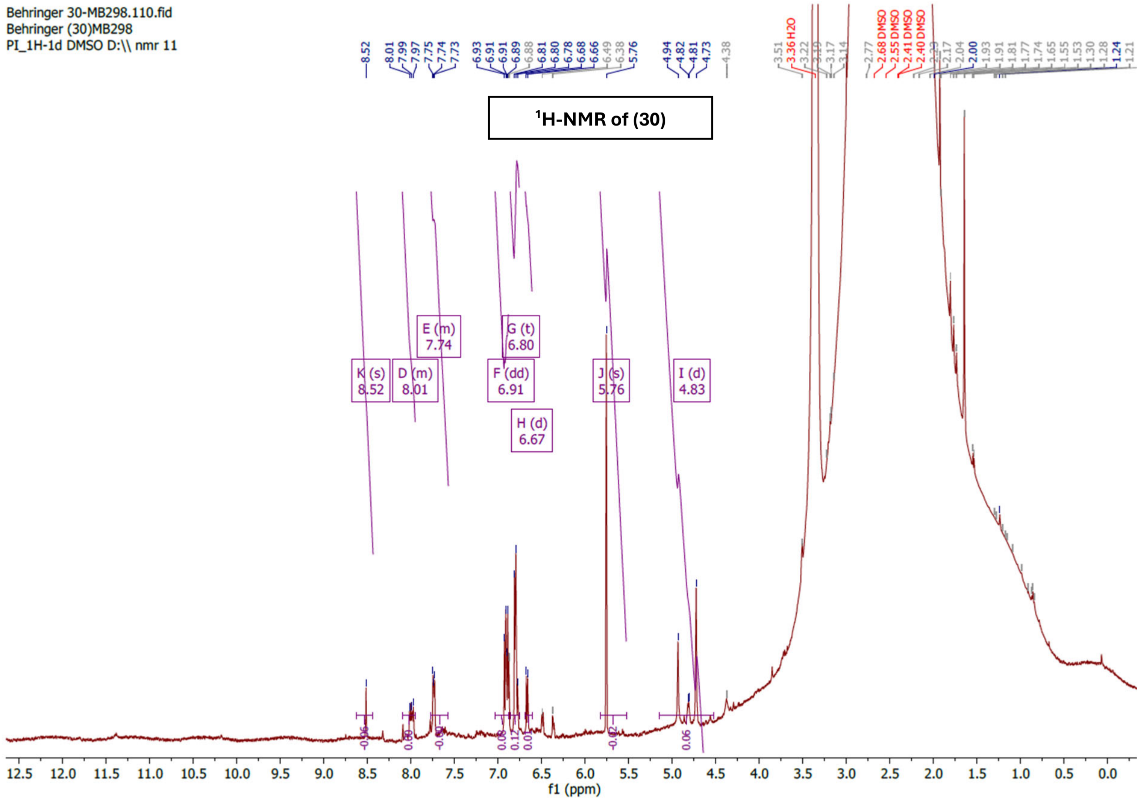

^{13}C-NMR of (30)

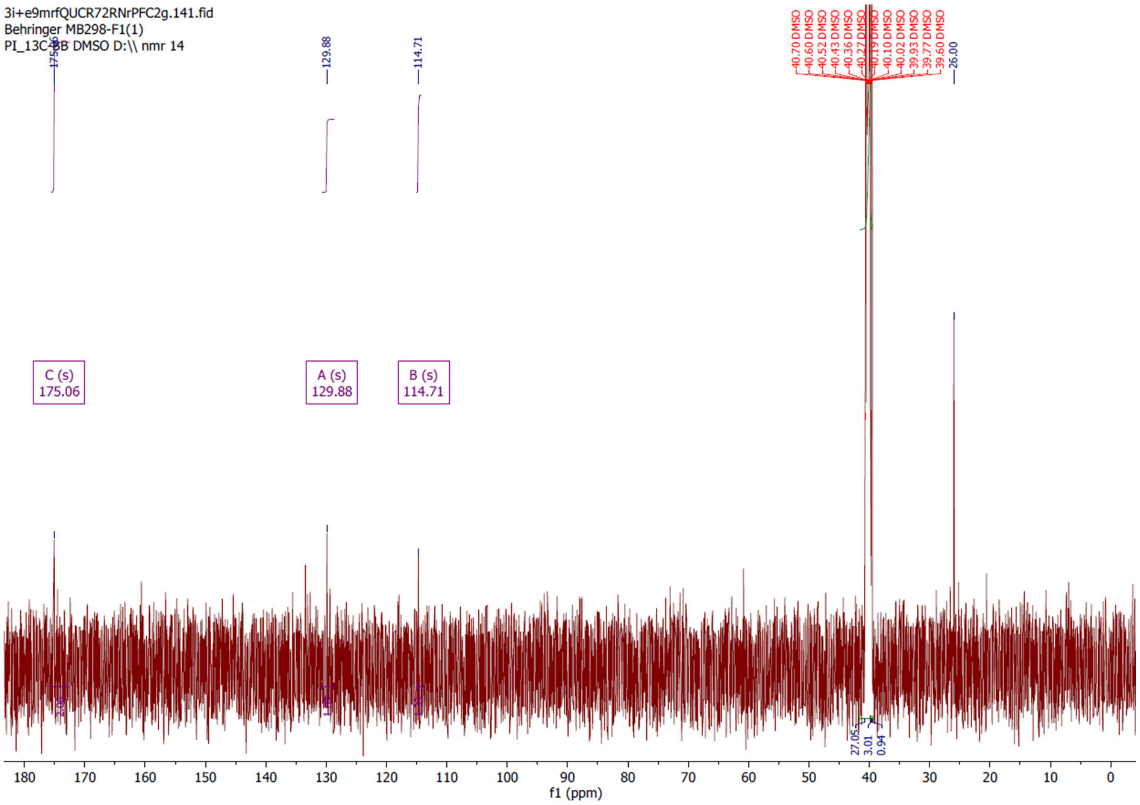
3i+e9mrfQUCR72RNrPFC2g.141.fid
Behringer MB298-F1(1)
PI_13C-BB DMSO D:\\ nmr 14
175.06
129.88
114.71
40.70 DMSO
40.60 DMSO
40.52 DMSO
40.43 DMSO
40.36 DMSO
40.27 DMSO
40.19 DMSO
40.10 DMSO
40.02 DMSO
39.93 DMSO
39.77 DMSO
39.60 DMSO
26.00
C (s)
175.06
A (s)
129.88
B (s)
114.71
27.05
3.01
0.94
180 170 160 150 140 130 120 110 100 90 80 70 60 50 40 30 20 10 0
f1 (ppm)

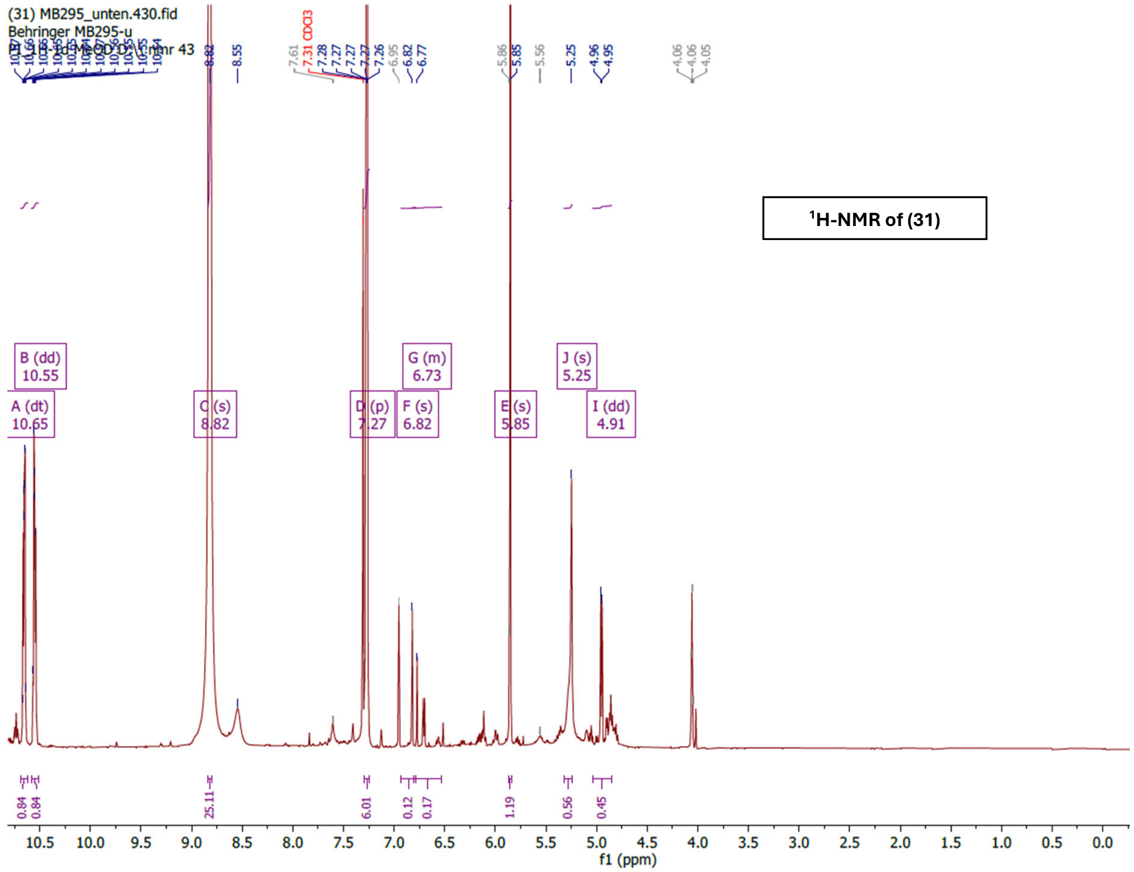
(31) MB295_unten.430.fid
Behringer MB295-u
¹H-NMR of (31)
A (dt) 10.65
B (dd) 10.55
C (s) 8.82
D (p) 7.27
F (s) 6.82
G (m) 6.73
E (s) 5.85
J (s) 5.25
I (dd) 4.91
f1 (ppm)

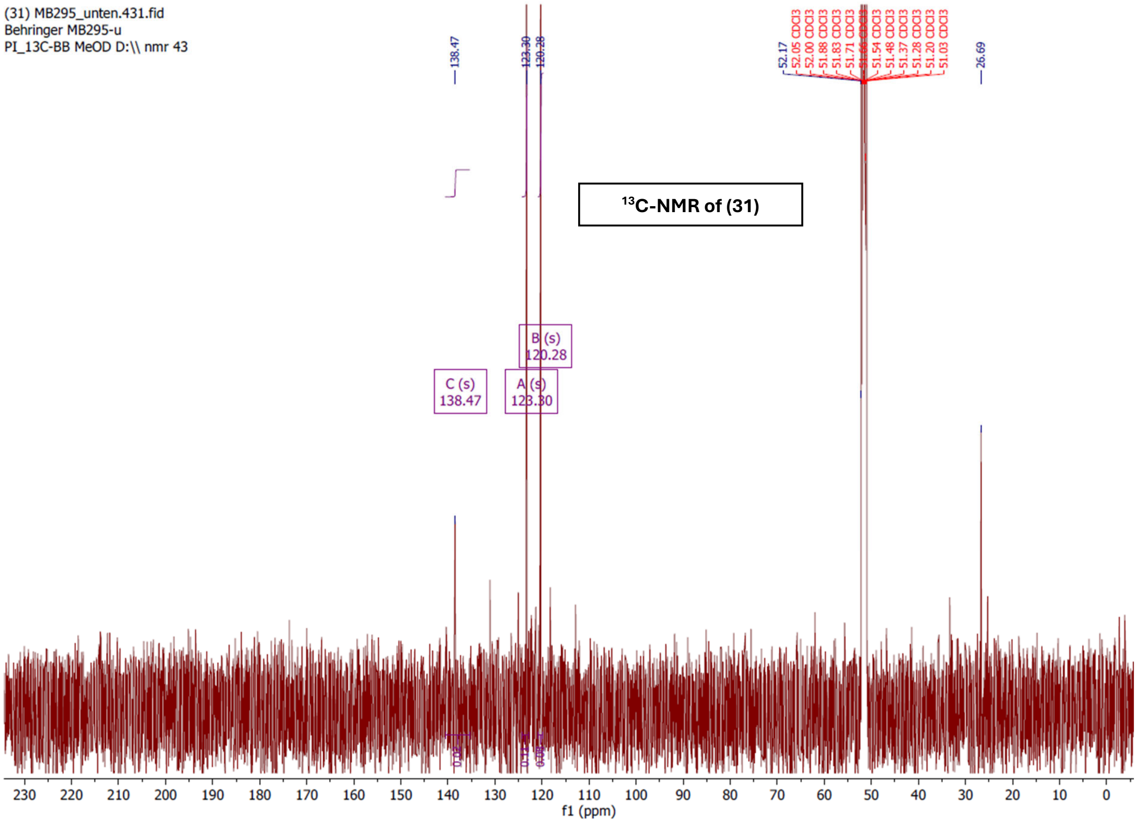
(31) MB295_unten.431.fid
Behringer MB295-u
PI_13C-BB MeOD D:\\ nmr 43
¹³C-NMR of (31)
C (s) 138.47
A (s) 123.30
B (s) 120.28
f1 (ppm)

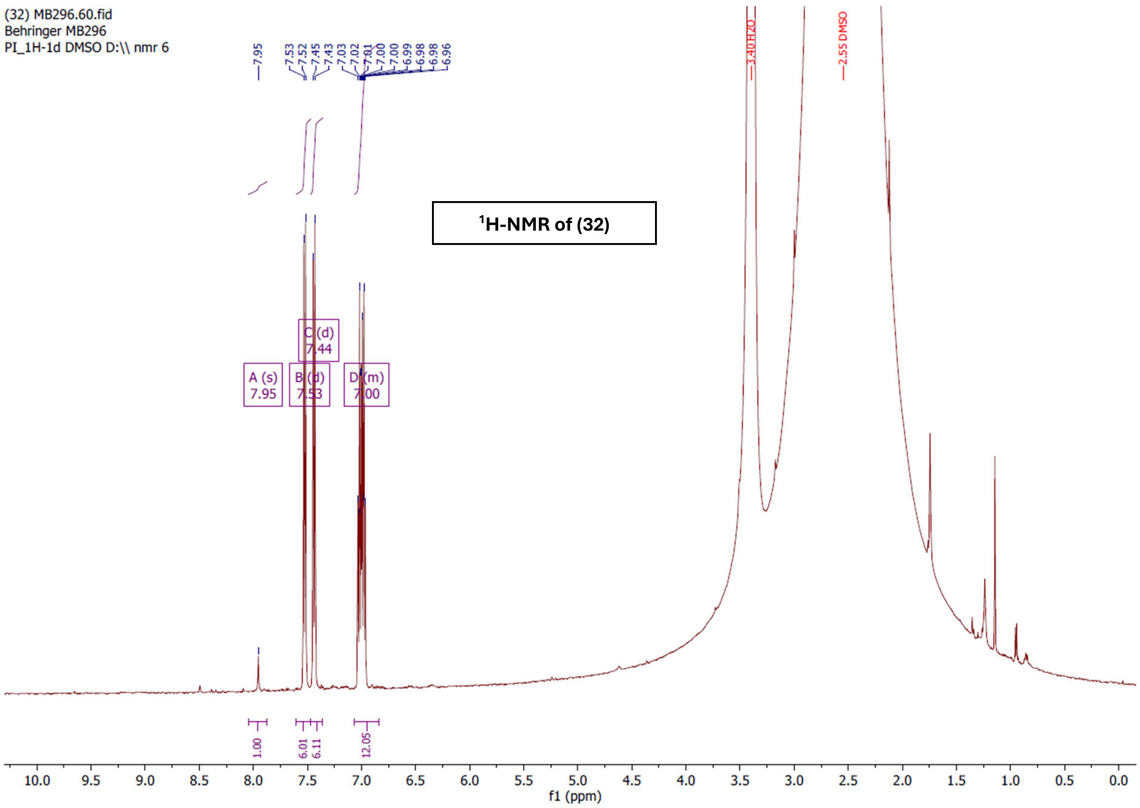
(32) MB296.60.fid
Behringer MB296
PI_1H-1d DMSO D:\\ nmr 6
1H-NMR of (32)
A (s) 7.95
B (d) 7.53
C (d) 7.44
D (m) 7.00
3.40 H2O
2.55 DMSO
f1 (ppm)

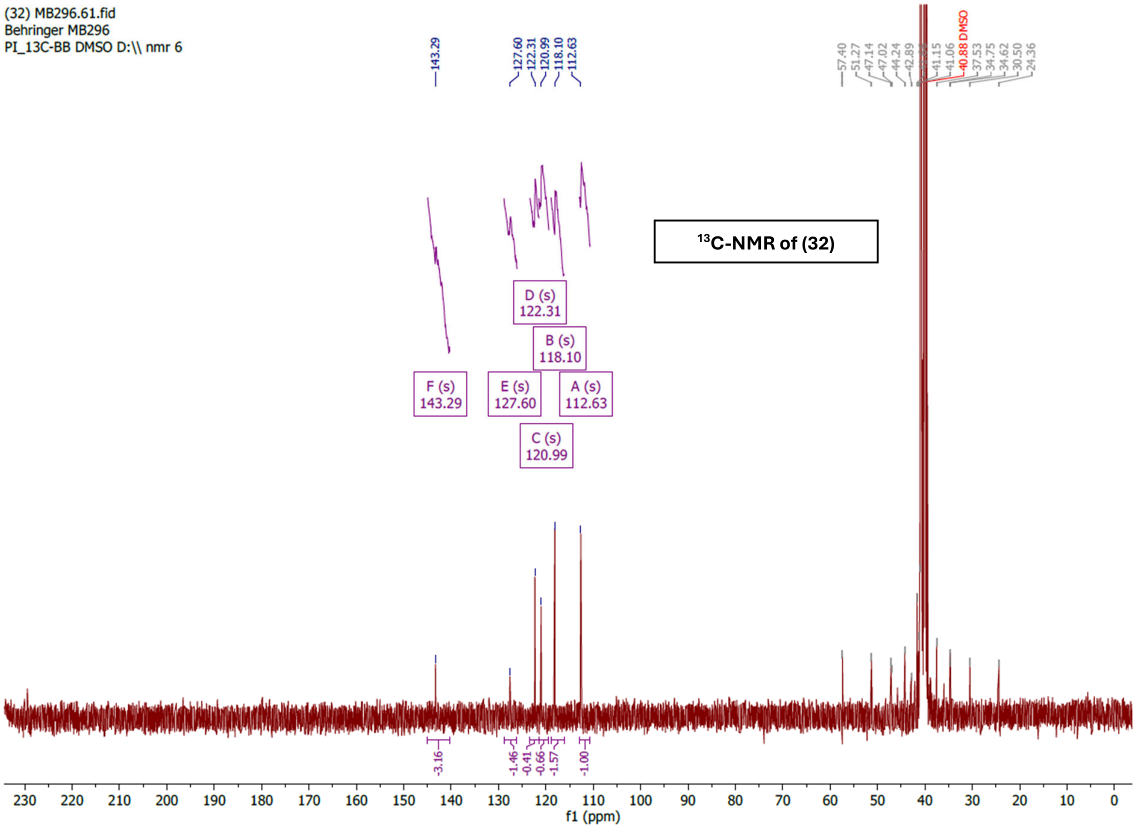
(32) MB296.61.fid
Behringer MB296
PI_13C-BB DMSO D:\\ nmr 6
13C-NMR of (32)
F (s) 143.29
E (s) 127.60
D (s) 122.31
C (s) 120.99
B (s) 118.10
A (s) 112.63
40.88 DMSO
f1 (ppm)

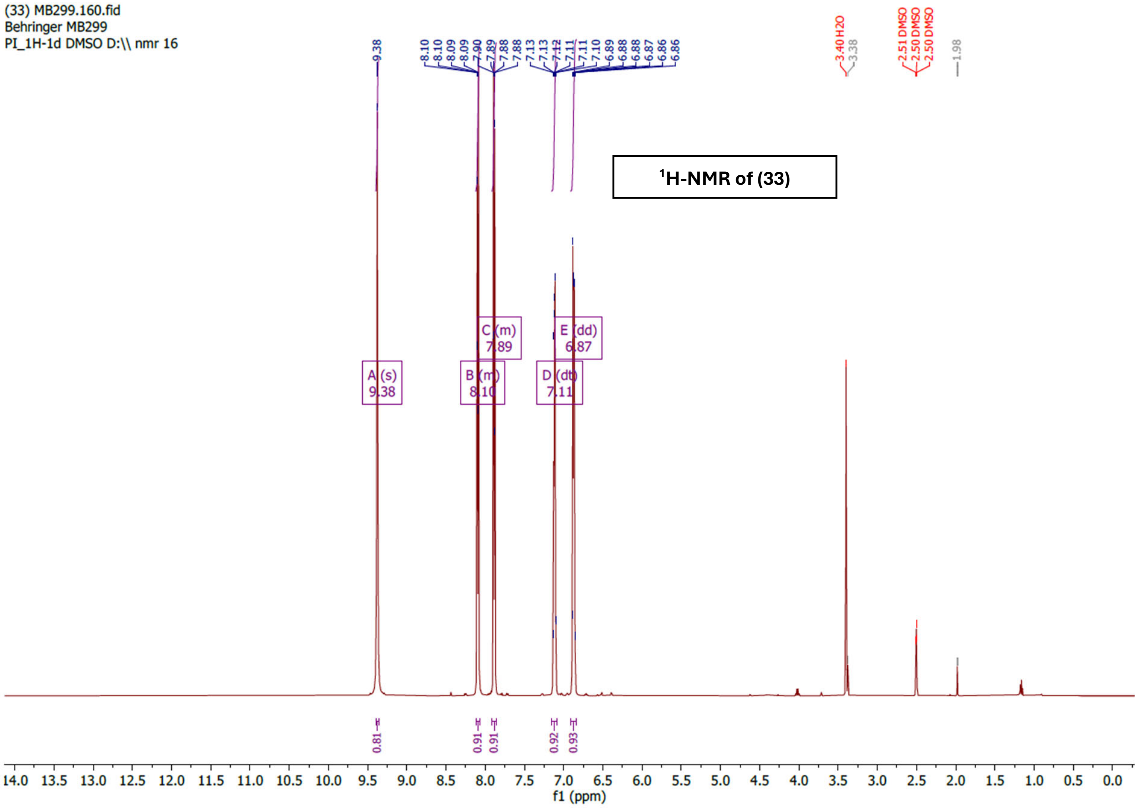
(33) MB299.160.fid
Behringer MB299
PI_1H-1d DMSO D:\\ nmr 16
1H-NMR of (33)
A (s) 9.38
B (m) 8.10
C (m) 7.89
D (dt) 7.11
E (dd) 6.87
f1 (ppm)

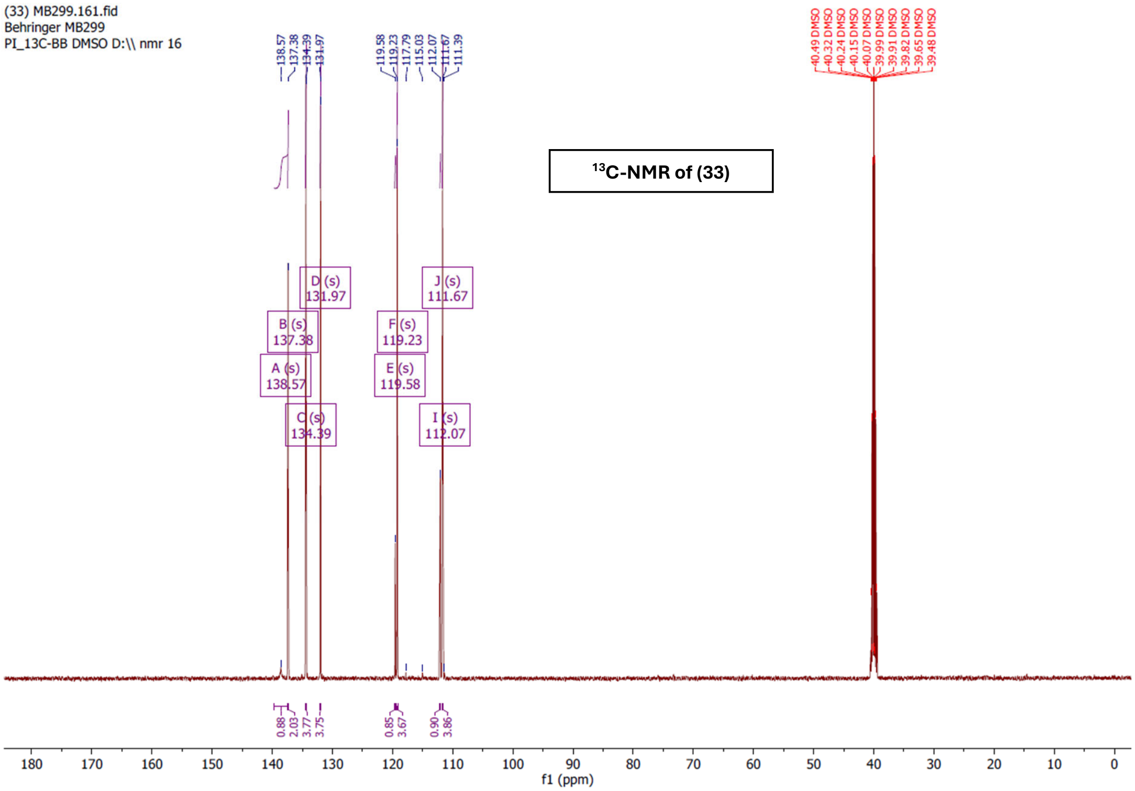
(33) MB299.161.fid
Behringer MB299
PI_13C-BB DMSO D:\\ nmr 16
13C-NMR of (33)
A (s) 138.57
B (s) 137.38
C (s) 134.39
D (s) 131.97
E (s) 119.58
F (s) 119.23
I (s) 112.07
J (s) 111.67
f1 (ppm)

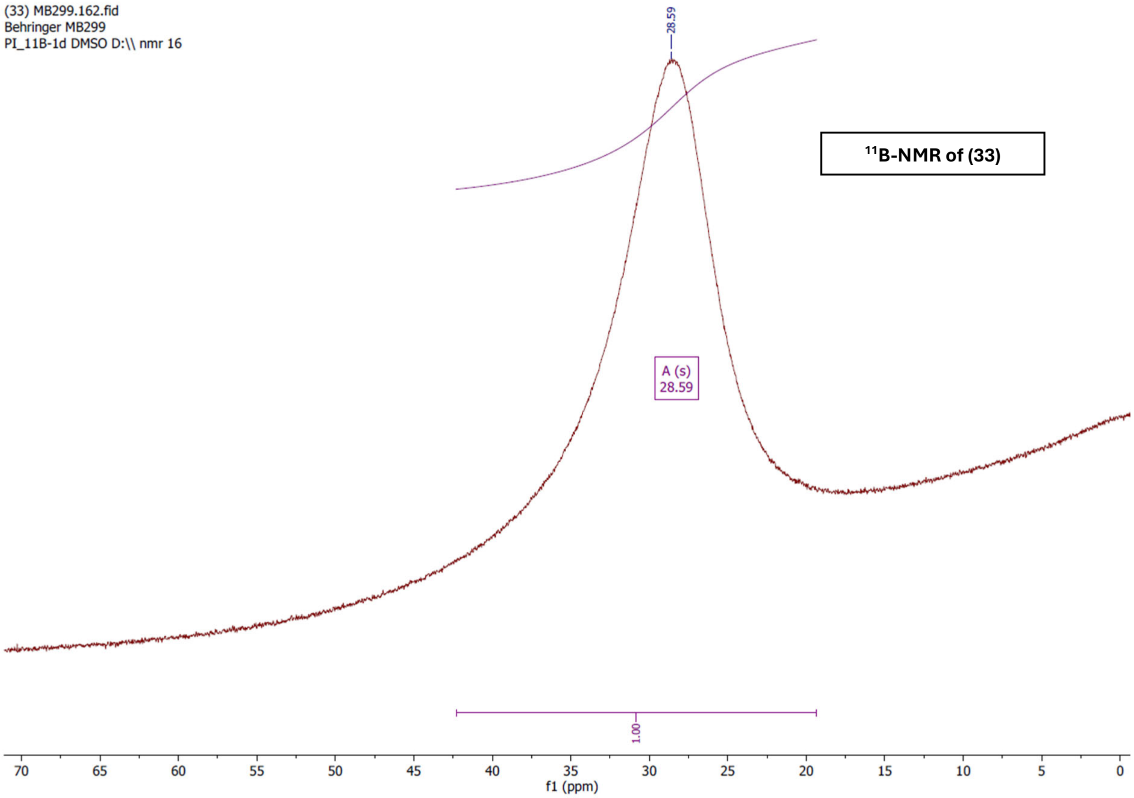
(33) MB299.162.fid
Behringer MB299
PI_11B-1d DMSO D:\\ nmr 16
11B-NMR of (33)
A (s)
28.59
1.00
f1 (ppm)

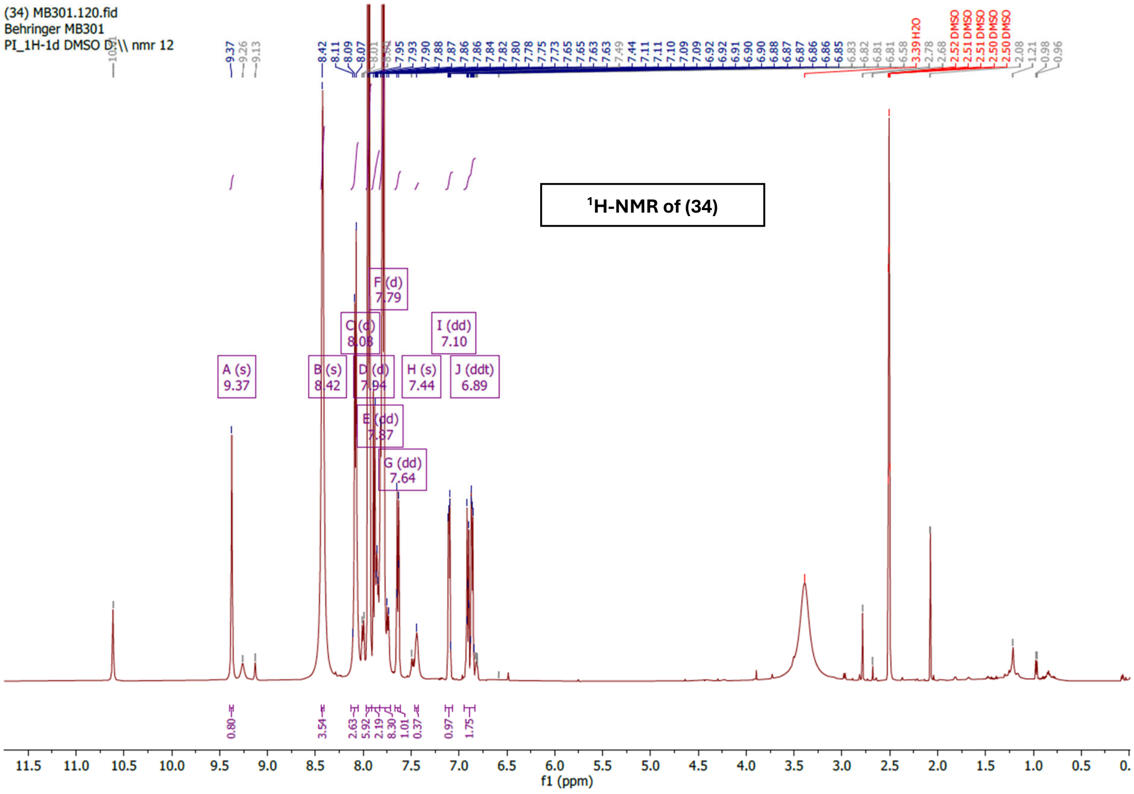
(34) MB301.120.fid
Behringer MB301
PI_1H-1d DMSO D:\\ nmr 12
1H-NMR of (34)
A (s) 9.37
B (s) 8.42
C (d) 8.08
D (d) 7.94
E (dd) 7.87
F (d) 7.79
G (dd) 7.64
H (s) 7.44
I (dd) 7.10
J (ddt) 6.89
3.39 H2O
2.52 DMSO
2.51 DMSO
2.51 DMSO
2.50 DMSO
2.50 DMSO
f1 (ppm)

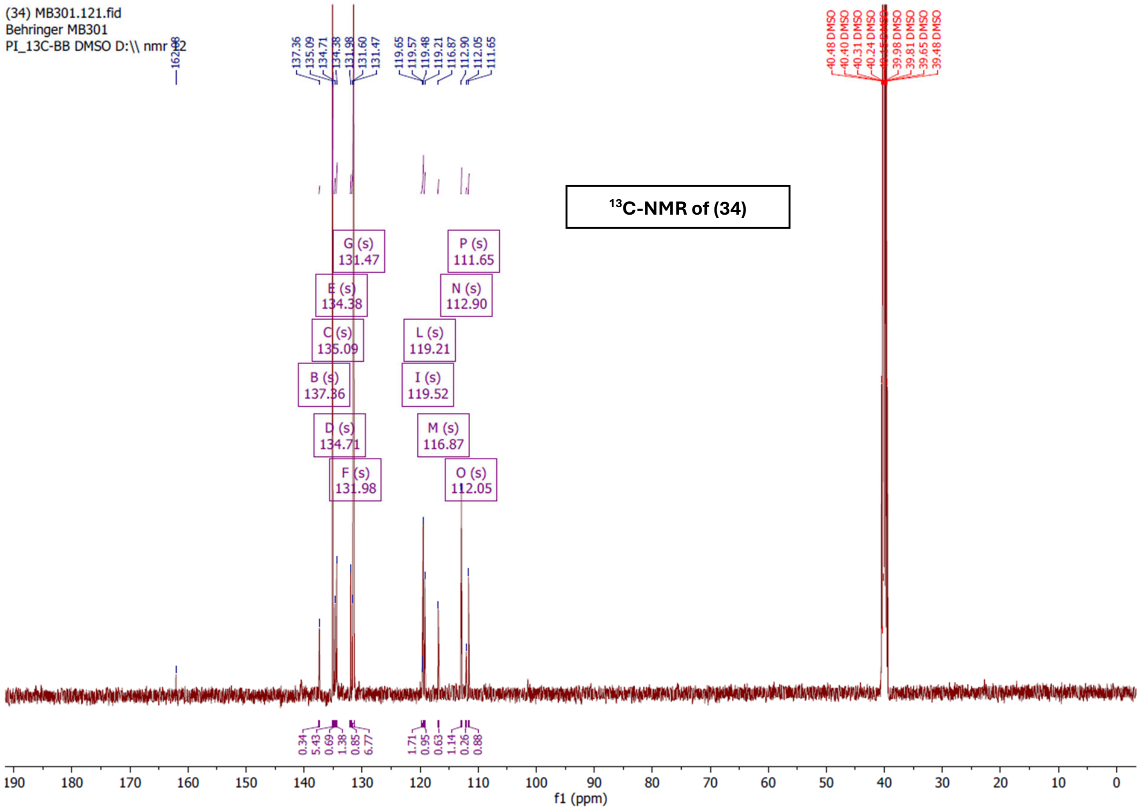
(34) MB301.121.fid
Behringer MB301
PI_13C-BB DMSO D:\\ nmr 12
13C-NMR of (34)
G (s) 131.47
P (s) 111.65
E (s) 134.38
N (s) 112.90
C (s) 135.09
L (s) 119.21
B (s) 137.36
I (s) 119.52
D (s) 134.71
M (s) 116.87
F (s) 131.98
O (s) 112.05
f1 (ppm)

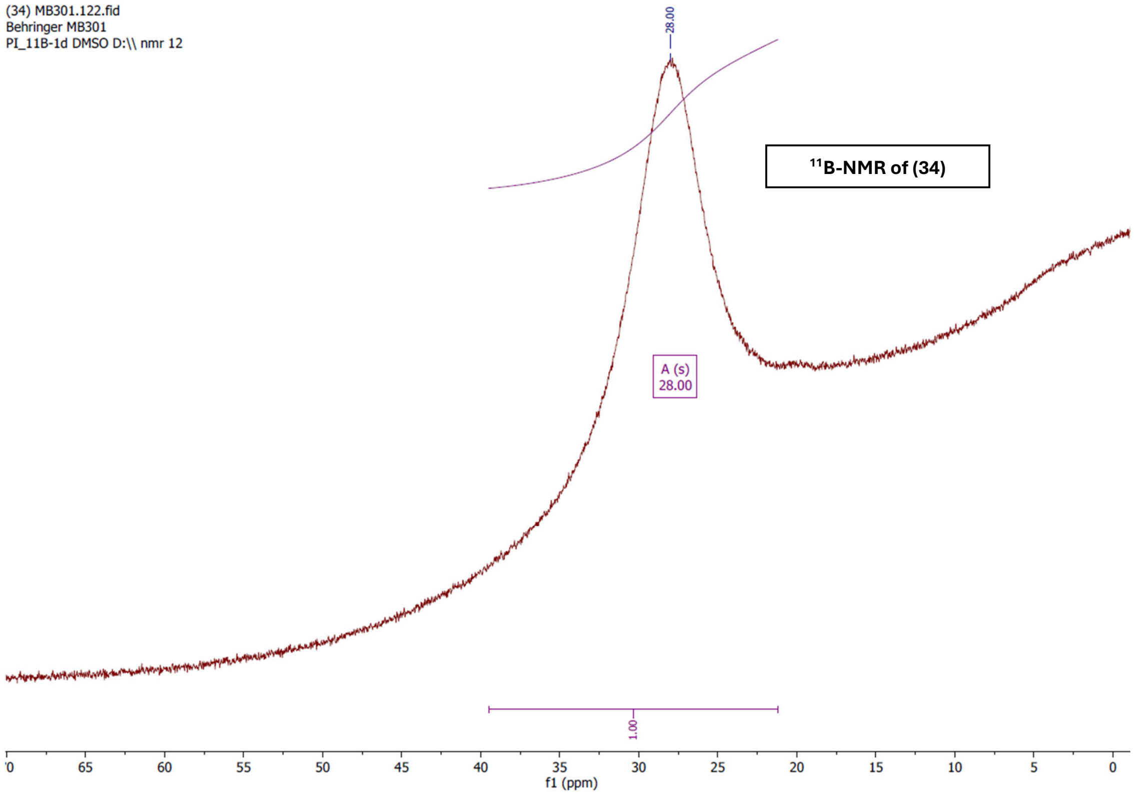
(34) MB301.122.fid
Behringer MB301
PI_11B-1d DMSO D:\\ nmr 12
28.00
11B-NMR of (34)
A (s) 28.00
1.00
f1 (ppm)

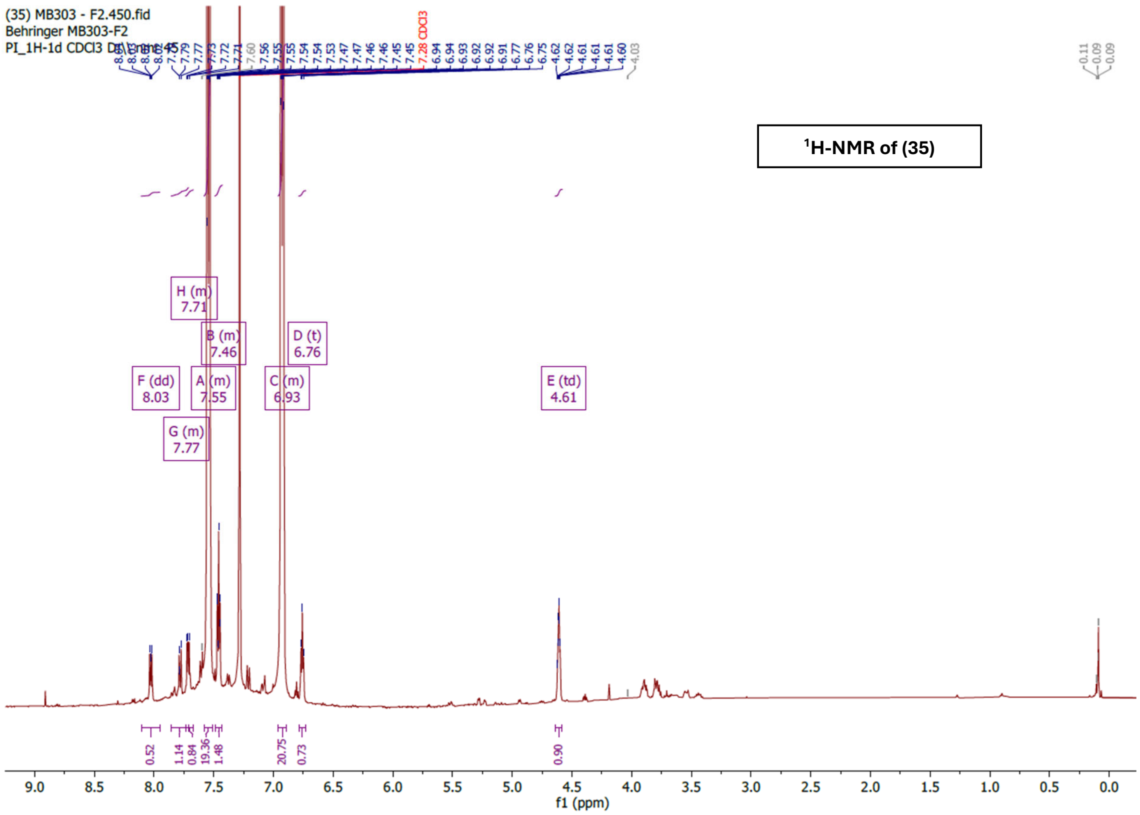

^{1}H-NMR of (35)

^{13}C-NMR of (35)

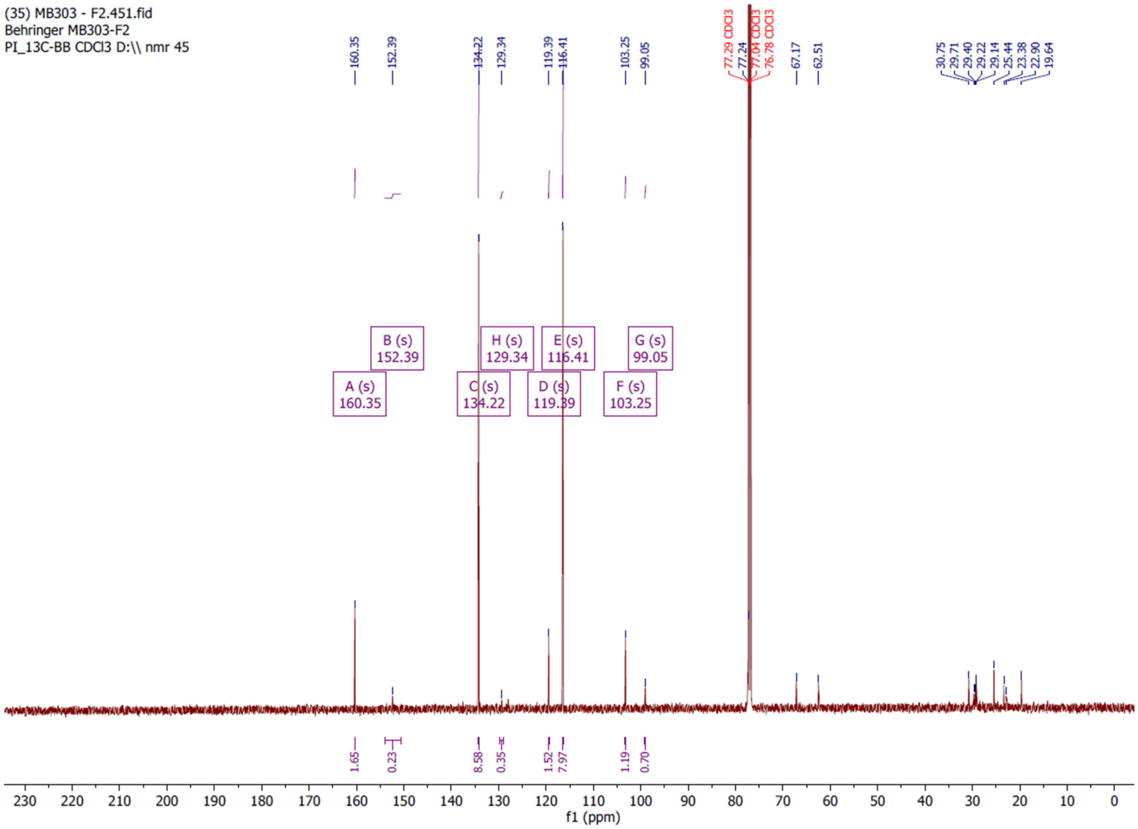

(35) MB303 - F2.451.fid
Behringer MB303-F2
PI_13C-BB CDCl3 D:\\ nmr 45
160.35
152.39
134.22
129.34
119.39
116.41
103.25
99.05
77.29 CDCl3
77.24
77.04 CDCl3
76.78 CDCl3
67.17
62.51
30.75
29.71
29.40
29.22
29.14
25.44
23.38
22.90
19.64
A (s) 160.35
B (s) 152.39
C (s) 134.22
H (s) 129.34
D (s) 119.39
E (s) 116.41
F (s) 103.25
G (s) 99.05
1.65
0.23
8.58
0.35
1.52
7.97
1.19
0.70
f1 (ppm)

3. Dose-response curves

The data represent means and standard deviations (N=3).

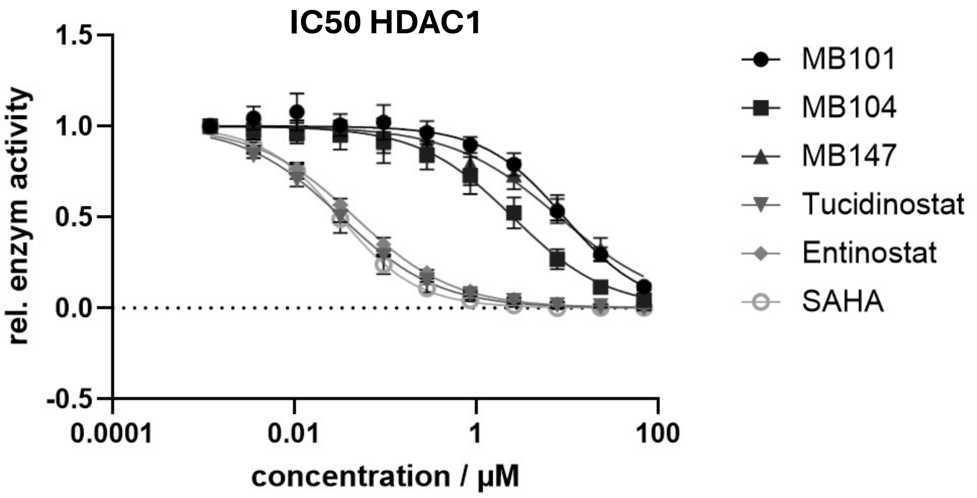

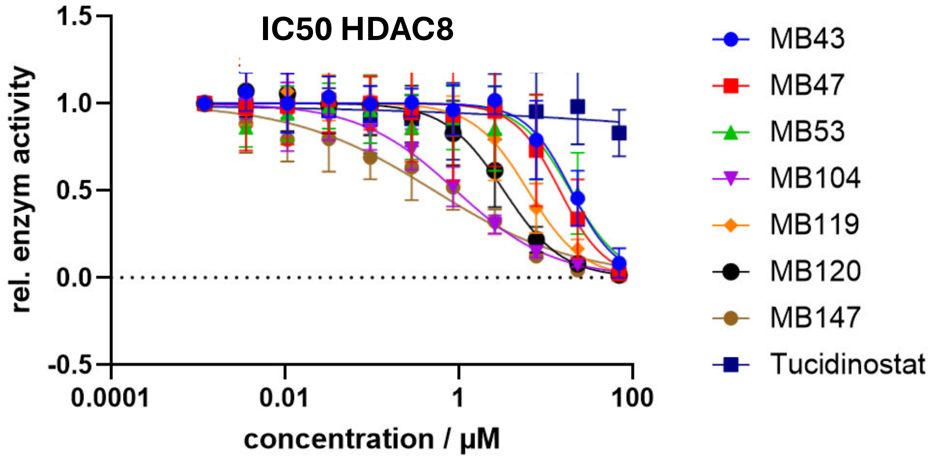

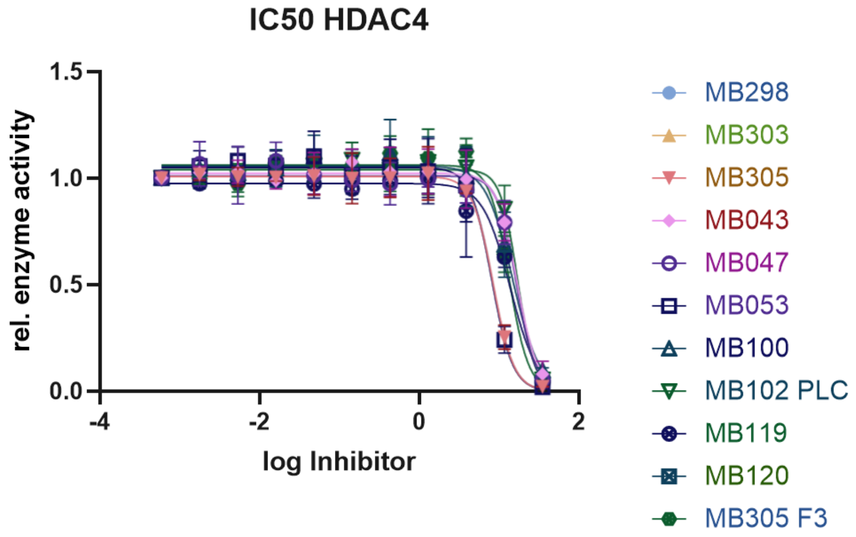
IC50 HDAC4
1.5
1.0
0.5
0.0
-4
-2
0
2
rel. enzyme activity
log Inhibitor
MB298
MB303
MB305
MB043
MB047
MB053
MB100
MB102 PLC
MB119
MB120
MB305 F3

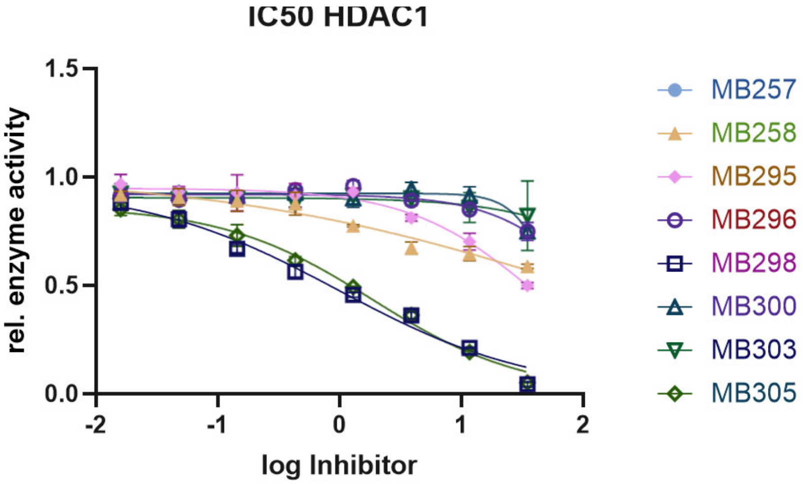
IC50 HDAC1
1.5
1.0
0.5
0.0
-2
-1
0
1
2
rel. enzyme activity
log Inhibitor
MB257
MB258
MB295
MB296
MB298
MB300
MB303
MB305

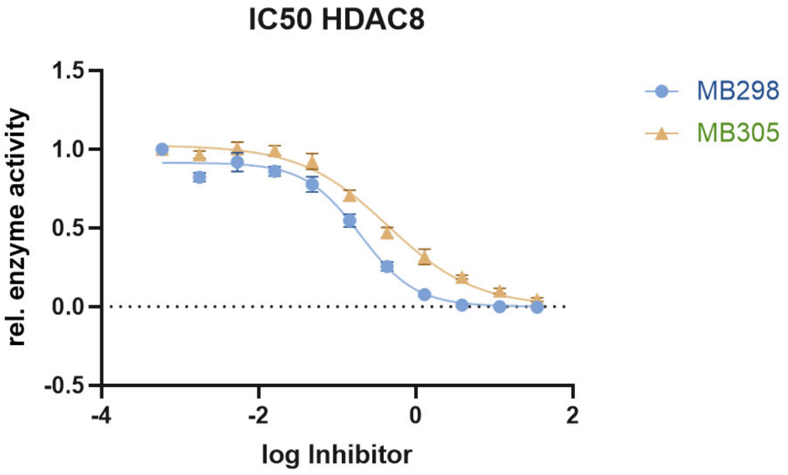
IC50 HDAC8
1.5
1.0
0.5
0.0
-0.5
-4
-2
0
2
rel. enzyme activity
log Inhibitor
MB298
MB305

Cpd-Label Dose-response curves	Cpd-Name in the manuscript
MB101	24
MB147	18
43	9
47	17
53	10
119	12
120	13
MB100	16d
MB257	20
MB258	21
MB295	31
MB296	32
MB298	30
MB300	22
MB303	35
MB305	23
MB102_PLC	25
